# Supplementary material for: Developing patient-centred, feasible alternative care for adult emergency department users with epilepsy: protocol for the mixed-methods observational ‘Collaborate’ project
Source: BMJ Open. 2019 Nov 2;9(11):e031696. doi: 10.1136/bmjopen-2019-031696 (PMC6830638; doi:10.1136/bmjopen-2019-031696)
Supplement: Supplementary data [file bmjopen-2019-031696supp001.pdf]

Supplementary File 1: Interview Topic Guide

Topics	Questions
Introduction	• Thank you very much for participating in this study. We are trying to find out more about how people with epilepsy would like to be cared for when they have a seizure and their views of the emergency services to contribute to the development of services. So I would very much like to hear about your own experiences.<br>• I wondered if you'd share with me your experiences with emergency services that may have led you to being interested in this study?
Carers	• Do you have a someone who helps with your epilepsy? (If yes, move on to support person questions. **If no, skip to next section**.)<br>• *(Prompts if needed: What is their relationship to you? i.e., family member, friend, paid carer? Do they live with you or somewhere else? How long have you known each other? How often would you usually see each other or have contact?)*<br>• How often have they been with you when you've had seizure in last 12 months?<br>• How confident do they feel in knowing what to do when a seizure happens?<br>• *Prompts if needed: Motives for contacting emergency services? Do you contact them every time after a seizure? What makes you feel more confident?*
Seizures	Perhaps first you could tell me about your seizures…<br>• What is your impression of how many seizures you have? *(Prompt: i.e., in the last year, month, year?)*<br>• How would you describe your seizures? *(Prompt: what kind do you usually have?)*<br>• How long have you been diagnosed with epilepsy?<br>• **Medication for seizures:** What do you take for your seizures? Are they helpful?<br>• How do you feel about managing your seizures? *(Prompt: do you have any strategies for managing your seizures?)*<br>• Are there any triggers or warnings before the onset of your seizures? *(Prompt: like an aura?)*<br>• How many times have emergency services been called in the past 12 months? How many of those times have you gone to A&E?
Decision Making	• I'm interested in your thoughts about the decisions that are made about your care following a seizure.<br>• Firstly, how often would you have seizures in **public** versus at **home**?<br>**Public place:**<br>• Can you think of a recent example and tell me where this was?<br>• Were you by yourself or with others (who?)

	• Was an ambulance called? What did they do? (i.e., take to A&E) Did you have any chance to discuss where you would like to go?<br>• Have you ever not been taken to A&E after seizure in a public place? *(Prompt: tell me more about what happened then. i.e., who was present, was this what you wanted?)*<br>• Do you have any emergency devices, or a medic alert jewellery?<br>**Home:**<br>• What do you do if a seizure happens when you are alone and no one else is around?<br>• Have **you** called an ambulance **for yourself** when you've had a seizure? If so, can you tell me a bit more about how you found this? How do you feel about the decision on whether or not to go to A&E? When **someone else** has called an ambulance after you've had a seizure, who typically makes the decision to call? Do you know if any alternatives to A&E were discussed? Who would typically make the decision for you to <u>go to A&E</u>?
**Emergency Services / A&E**	• Can you tell me more about what your general experiences of **<u>Emergency Services</u>** for your epilepsy?<br>◦ *(Prompts if needed: Where are you when the seizure(s) happen (situation and circumstances)? Do you go usually go to A&E? Who makes decision/organised E.g., carer/relative, other lay people, ambulance staff, police, etc. Is this usually what you would want?)*<br>• When was the last time you used emergency services and didn't use A&E? What happened?<br>◦ What kinds of things might change how you use emergency services? (i.e., such as location, seizure type)<br>• Can you tell me about the last time you went to **<u>A&E</u>** for your epilepsy?<br>◦ *Prompts if needed: Why did you go to A&E? Who made decision/organised E.g., carer/relative, other lay people, ambulance staff, police, etc.*<br>• Would you say this is typical for you?<br>• *Was this what you would have wanted?*
**Views of A&E**	If you had to use emergency services again, are there any changes that you would like to see in the way this service is provided? (Prompt if needed: and how about A&E?)<br>• What do you believe is most helpful about going to A&E? *E.g., Reassurance, confidence, treatments available, reduce burden on others. (write down)*<br>• Do you see there being anything negative about going to A&E? *E.g., Time, not necessary, unnecessary tests and examinations, disrupted rest, don't like hospitals/being patient, etc. (write down)*<br>• What has been your experience of the link-up/ communication between A&E and your usual care provider?<br>• Have your usual care providers been informed of A&E visits?<br>• Have you been contacted and offered any extra support because you had been to A&E?

<table>
<tr>
<td>Alternatives to A&E</td>
<td>

When you have had a seizure and contact has been made with emergency services (i.e., 999/paramedics/111), have you ever been offered an alternative to A&E? If so, what?
If you weren't taken to A&E, where would you like to be taken once your seizure had finished? What health professional would you like to see at the time? What kind of follow-up would you like to have?

Are you familiar with something called an Urgent Treatment Centre?
Extra Explanation, only if needed:
The term Urgent Treatment Centres is a relatively new one, but it has been suggested as a potential alternative to A&E. To give you a feel of what they are, the idea is that: -They will be open at least 12 hours a day, 7 days a week. Staffed by GPs and nurses. -Be able to issue prescriptions and have access to some common, but basic equipment, such as ECGs to test heart function and in some cases X-ray machines.

If yes, have you ever been to one before?
If no, you might be more familiar with terms like 'walk-in centres' and 'minor injury units'. These are to be relabelled urgent treatment centres. -How many there are located near you, and where they are, varies. Some are located next to GP practices, some are on hospital sites

What might be your expectations of such a centre be?
Would you have any concerns about this? If so, what kind?
Would anything change how you felt about this ACP? Ie., seizure type, recent changes in meds

Another option that is being considered instead of always taking people with epilepsy to A&E following a seizure, is for the person to be left at home or taken home if they were out. They would then be telephoned within say 24 hours by an epilepsy nurse specialist. The nurse would be phoning to see how the person was recovering and whether needed any additional support, such as a change in medication, advice or to be booked in to see a neurologist.
Extra Explanation, only if needed/not seen an ENS before:
-An epilepsy nurse is a registered nurse who typically has extra experience or qualifications in neurology. Their role varies but they can provide information regarding epilepsy and its management, they can help monitor and change medication, order tests and act as a first point of contact for patients and GPs. They advise people in a variety of settings, including in outpatient clinics and over the phone.

What might be your expectations be over this sort of alternative to being taken to A&E?
Would you have any concerns about this? If so, what kind?
Would anything change how you felt about this ACP? Ie., seizure type, recent changes in meds

</td>
</tr>
</table>

**Discrete Choice**	• Complete Discrete Choice / Ranking Exercise
**Characteristics**	Now I have some final short-answer questions to run through about you…<br>• What is your Date of birth?<br>• **Highest level of completed education?** (no formal qualification, 1-4GSCE, 5 or more GSCE, Apprenticeship, 2 or more A-levels, First of higher degree/professional qualification/equivalent higher education, vocational/work-related training).<br>• **Ethnicity?** (i.e., white, mixed, Asian/Asian British, Black or Black British, Chinese or other ethnic group).<br>• **Household:** What are your living arrangements? (living with others, living alone)<br>• **Other conditions:** Have you ever been diagnosed with a learning disability? Do you have any mobility difficulties?<br>**Healthcare Professionals:** Who would you contact if anything changes with your epilepsy? Have you seen them in the past 12 months?<br>• Have you seen any other people for help with your epilepsy within the last 12 months? E.g., neurologist, epilepsy nurse, GP with specialist interest in epilepsy, learning disability psychiatrist, paediatrician, neurosurgeon.
**Closing**	• Is there anything you'd like to mention about your experience of emergency services for seizure care that we haven't already discussed?<br>• Thank you very much for your time and for answering my questions. There will be a results report available at the end of the study – would you like to receive a copy?<br>• Do you have any final questions?

**Ranking exercise**	**We are keen to understand what type of care people would prefer when they have a seizure.**<br><br>**Here are some factors** that you have mentioned / or we think may be important …**<br><br>*Interviewer displays cards (Appendix 1) in a random order in front of the participant.*<br><br>Looking at these cards:<br><br>1. **Is there anything missing?** Are there any other factors what would affect your choice?<br><br>*Interviewer writes additional factors on blank cards and places them alongside the pre-defined cards in front of the participant.* ***Record any self-nominated factors on the interview record sheet (Appendix 2).***<br><br>2. **Which are the most important factors in your opinion?** Can you pick up all the cards that would most likely affect your decision?

	Explain that these factors can have been experienced, or not; and include any that were self-nominated in this exercise. Clear the remaining cards from the table.<br><br>**3. Now, can you rank these in order of most important to least important?**<br><br>*Check the order the interviewer places the cards is i.e. So, you think 'X' at the top is the most important? Encourage the participant to "think aloud" and explain their ranking. If participants find it hard to rank certain factors, or align them side by side, explore why.* ***Record the rank scores on the interview record sheet.***

Appendix 1: Show cards

Where the ambulance takes you

Where the ambulance takes you (e.g. A&E, Urgent treatment centre).

Care provider

The healthcare professional responsible for your care when you get there.

Waiting time

How long you have to wait before you see the healthcare professional.

Tests

The type of tests the health professional could carry out immediately if needed

Follow-up

Referral to see a health professional with specialist training in epilepsy

Appendix 2: Ranking exercise interview record sheet

Participant ID: _ _ /_ _ / _ _

Interviewer ID _ _

**Factor***	Rank Results (insert 1-5)<br>1 = most important<br>5 = least important<br>X = not selected
Anything Else? **Write here:**	
Anything Else? **Write here:**	
Anything Else? **Write here:**	
Anything Else? **Write here:**	
Anything Else? **Write here:**	
Anything Else? **Write here:**	
Where the ambulance takes you	
Care provider	
Waiting time	
Tests	
Follow-up	
