# Supplementary material for: Developing patient-centred, feasible alternative care for adult emergency department users with epilepsy: protocol for the mixed-methods observational ‘Collaborate’ project
Source: BMJ Open. 2019 Nov 2;9(11):e031696. doi: 10.1136/bmjopen-2019-031696 (PMC6830638; doi:10.1136/bmjopen-2019-031696)
Supplement: Supplementary data [file bmjopen-2019-031696supp002.pdf]

Supplementary File 2 Illustration of a possible vignette and Discrete Choice Experiment choice that could be used

Question 1
Imagine you have a seizure in a public place. It lasted no more than usual and stopped by itself. You are tired and need to sleep. Someone called an ambulance and the paramedic is with you. Which of the following options would you prefer to happen next?

	**Option A**	**Option B**
**Where**<br><br>*Where the ambulance takes you*	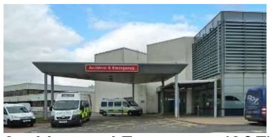<br>***Accident and Emergency (A&E)***	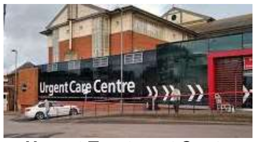<br>***Urgent Treatment Centre***
**Care provider**<br><br>*The health care professional responsible for your care when you get there*	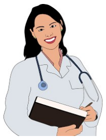<br>***Consultant doctor in emergency medicine***	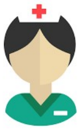<br>***Emergency nurse practitioner***
**Waiting time**<br><br>*How long you have to wait before you see the health care professional*	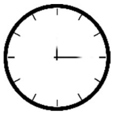<br>***4 hours***	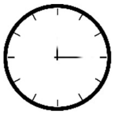<br>***1 hour***
**Tests**<br><br>*The type of tests the health professional could carry out immediately if needed*	***Advanced***<br>(examples...)	***Basic***<br>(examples...)
**Follow-up**<br><br>*The number of people referred to see a health professional with specialist training in epilepsy*	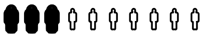<br>***3 in 10 people***<br>***referred to specialist epilepsy services***	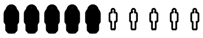<br>***5 in 10 people***<br>***referred to specialist epilepsy services***
**Which service would you prefer?**	☐	☐
