# Supplementary material for: Developing patient-centred, feasible alternative care for adult emergency department users with epilepsy: protocol for the mixed-methods observational ‘Collaborate’ project
Source: BMJ Open. 2019 Nov 2;9(11):e031696. doi: 10.1136/bmjopen-2019-031696 (PMC6830638; doi:10.1136/bmjopen-2019-031696)
Supplement: Author's manuscript [file bmjopen-2019-031696.draft_revisions.pdf]

BMJ Open is committed to open peer review. As part of this commitment we make the peer review history of every article we publish publicly available.

When an article is published we post the peer reviewers' comments and the authors' responses online. We also post the versions of the paper that were used during peer review. These are the versions that the peer review comments apply to.

The versions of the paper that follow are the versions that were submitted during the peer review process. They are not the versions of record or the final published versions. They should not be cited or distributed as the published version of this manuscript.

BMJ Open is an open access journal and the full, final, typeset and author-corrected version of record of the manuscript is available on our site with no access controls, subscription charges or pay-per-view fees (http://bmjopen.bmj.com).

If you have any questions on BMJ Open's open peer review process please email info.bmjopen@bmj.com

BMJ Open

Developing patient-centred, feasible alternative care for emergency department users with epilepsy: Protocol for the mixed-methods observational 'Collaborate' project

Journal:	*BMJ Open*
Manuscript ID	bmjopen-2019-031696
Article Type:	Protocol
Date Submitted by the Author:	16-May-2019
Complete List of Authors:	Noble, Adam; University of Liverpool, Department of Health Services Research<br>Mathieson, Amy; University of Liverpool, Department of Health Services Research<br>Ridsdale, Leone; King's College London, Department of Basic and Clinical Neuroscience, Institute of Psychiatry, Psychology & Neuroscience<br>Holmes, EA; Centre for Health Economics & Medicines Evaluation<br>Morgan, Myfanwy; King's College London, Institute of Pharmaceutical Science<br>Dickson, Jon; The University of Sheffield, Academic Unit of Primary Medical Care<br>Jackson, Mike; North West Ambulance Service NHS Trust<br>Hughes, Dyfrig; Bangor University, Centre for Health Economics and Medicines Evaluation<br>Goodacre, Steve; University of Sheffield, Medical Care Research Unit<br>Marson, Anthony; University of Liverpool, Department of Molecular and Clinical Pharmacology
Keywords:	Epilepsy < NEUROLOGY, ACCIDENT & EMERGENCY MEDICINE, Organisation of health services < HEALTH SERVICES ADMINISTRATION & MANAGEMENT, HEALTH ECONOMICS, QUALITATIVE RESEARCH

SCHOLARONE™
Manuscripts

TITLE: Developing patient-centred, feasible alternative care for emergency department users with epilepsy: Protocol for the mixed-methods observational 'Collaborate' project.

AUTHORS: Adam J. Noble,[1] Amy Mathieson,[1] Leone Ridsdale,[2] Emily Holmes,[3] Myfanwy Morgan,[4] Jon Dickson,[5] Mike Jackson,[6] Dyfrig Hughes,[3] Steve Goodacre,[7] Anthony G. Marson.[8]

AFFILIATIONS:

[1] Department of Health Services Research, University of Liverpool, UK.

[2] Department of Basic and Clinical Neuroscience, King's College London, UK.

[3] Centre for Health Economics & Medicines Evaluation, Bangor University, UK.

[4] Institute of Pharmaceutical Science, King's College London, UK.

[5] Academic Unit of Primary Medical Care, University of Sheffield, UK.

[6] North West Ambulance Service NHS Trust, Bolton, UK.

[7] School of Health and Related Research, University of Sheffield, UK.

[8] Department of Molecular and Clinical Pharmacology, University of Liverpool. UK.

FOR CORRESPONDENCE: Name: Adam Noble PhD; Address: University of Liverpool, Department of Health Services Research, Waterhouse Building, Liverpool, L69 3GB, United Kingdom.; Telephone: +44 151 794 5993; E-mail: adam.noble@liverpool.ac.uk

ABSTRACT

Introduction

Emergency department (ED) visits for epilepsy are common, costly, often clinically unnecessary and typically lead to little benefit for epilepsy management. An 'Alternative Care Pathway' (ACP) for epilepsy, which diverts people with epilepsy (PWE) away from ED when '999' is called and leads to care elsewhere, might generate savings and facilitate improved ambulatory care. It is unknown though what features it should incorporate to make it acceptable to persons from the particularly vulnerable target population. It also needs to be National Health Service (NHS) feasible. This project seeks to identify the optimal ACP configuration.

Methods and analysis

Mixed-methods project comprising 3-linked stages. In Stage 1, NHS bodies will be surveyed on ACPs they are considering and semi-structured interviews with PWE and their carers will explore attributes of care important to them and their concerns and expectations regarding ACPs. In Stage 2, Discrete Choice Experiments (DCE) will be completed with PWE and carers

For peer review only - http://bmjopen.bmj.com/site/about/guidelines.xhtml

to identify the relative importance placed on different care attributes under common seizure scenarios and the trade-offs people are willing to make. The uptake of different ACP configurations will be estimated. In Stage 3, two Knowledge Exchange workshops using a nominal group technique will be run. NHS managers, health professionals, commissioners, and patient and carer representatives will discuss DCE results and form a consensus on which ACP configuration best meets users' needs and is NHS feasible.

Ethics and dissemination

Ethical approval: NRES Committee (19/WM/0012) and King's College London ethics Committee (LRS-18/19-10353). Primary output will be identification of optimal ACP configuration which should be prioritised for implementation and evaluation. A pro-active dissemination strategy will make those considering developing or supporting an epilepsy ACP aware of the project and opportunities to take part in it. It will also ensure they are informed of its findings .

Project registration: researchregistry4723.

Strengths and limitations of this study (5 bullet points; one sentence each, relating to the methods)

- Project seeks to identify the optimal configuration of an 'Alternative Care Pathway' for epilepsy which could minimise clinically unnecessary and/or avoidable unplanned health service use and facilitate improved outcomes.
- The novel co-production approach employed – which will involve stakeholders at each project stage and use Discrete Choice Experiments – will allow service users to inform service design in a scientifically robust and non-tokenistic way.
- This study could provide a template for service design more generally within the National Health Service (NHS).
- The ACP configuration identified will be developed for use in the NHS and so may not be immediately applicable to other health systems. It will also require evaluation via a subsequent project.
- The project's success will depend, in part, on service users from what is a particularly vulnerable population agreeing to participate and representative samples being generated.

Key words: Epilepsy; Accident & Emergency Medicine; Organisation of health services; Heath economics; Qualitative Research.

For peer review only - http://bmjopen.bmj.com/site/about/guidelines.xhtml

4,235 words.

For peer review only

For peer review only - http://bmjopen.bmj.com/site/about/guidelines.xhtml

INTRODUCTION

Context and drive for health service innovations

The National Health Service (NHS), like other health systems, is being asked to make cost savings, whilst improving care experience, outcomes, and reducing health inequalities [1 2]. Emergency department (ED) visits and admissions for the ambulatory care sensitive condition epilepsy provide an opportunity where service innovations could help achieve such aims. We describe a mixed-methods project to maximise the likelihood of such innovations being beneficial to service users and society.

Epilepsy and unplanned service use

With a prevalence of ~1%,[3] epilepsy is the UK's second most common serious neurological disorder. Annually, ≤20% of people with epilepsy (PWE) visit hospital emergency departments (ED).[4-6] They report more anxiety, seizures and perceived epilepsy stigma than those in the wider epilepsy population and live in more deprived areas [7-12]; ~20% have an intellectual disability [13 14].

The annual cost to the NHS of these visits in England alone is ~£70-90M.[15-17] Costs are high because half of the visits result in hospital admission.[6 18-21] An unusually high re-attendance rate also inflates cost;[22 23] ≤60% of PWE re-attend ED within 12 months. [7]

Most PWE visiting ED do not attend with a life threatening or emergency presentation, (e.g., status epilepticus, first seizure). Rather, projects like our National Audits of Seizure Management in Hospitals (NASH) [13 14] show that most have known, rather than new epilepsy, and present with states not requiring ED's full facilities. Leading presentations include someone who has experienced: i) an uncomplicated seizure in line with their usual presentation; ii) a seizure in public and cannot be 'left at scene'); and iii) a self-terminating seizure, different to their usual presentation.[20]

Unmet need PWE visiting ED

Whilst the acute episodes leading PWE to visit ED do not typically require emergency care, attendees do often require improvements in ambulatory care to prevent re-attendance.

NASH [24], for example, found most (~65%) PWE visiting ED are not known to specialist epilepsy services and many are prescribed suboptimal treatment. NICE [2] recommend when seizures are not controlled, a patient should be referred to specialist services since this can improve outcomes, including rendering some seizure free.[25 26]

Some ED attendees also need enhanced self-management support, like that provided by epilepsy nurses, so they and their family can manage seizures.[7 27-29] There is no routine course PWE can go on to learn about epilepsy. Consequently, knowledge can be low, particularly in those with lower education levels.[30 31]

For peer review only - http://bmjopen.bmj.com/site/about/guidelines.xhtml

Going to ED does not currently lead to PWE typically receiving ambulatory care improvements. Most (80%) are not seen by a specialist during their attendance, usual care providers may not be informed of the attendance [13] and most (60%) PWE are not referred to a specialist for follow-up. PWE living in the most deprived areas are least likely to be referred [32].

Ensuring PWE in need of specialist care receive it has been a longstanding challenge. Possible reasons include the UK's small number of neurologists [33] and the lack of incentives for the identification of such PWE. An indication of the challenge is that trial evidence indicates ~70% of PWE can become seizure free, but only ~50% of PWE in the UK currently are [34]. PWE in socially deprived areas again fare worst [35].

An 'Alternative Care Pathway'

NHS policies [1 2 36] and publicity given to NASH's findings [15 37] created momentum to reduce visits for seizures and enhance patient outcomes. NICE [38] found no evidence on how to do this. However, one idea gaining traction is for ambulance services to assume a greater role.[39] Most (~90%) people visiting ED for seizures have been transported there by an emergency ambulance.[13 14 21]

Data from some regional ambulance services on conveyance rates for seizures has been published. It indicates ambulances are currently conveying nearly every person they attend for a suspected seizure to ED,[40-42] despite most not demonstrating a clinical need (e.g., seizures have self-terminated before ambulance arrival in ~90% of cases),[41] One reason for this is paramedics lack access to alternatives.[43-45] There is a vision therefore of what could help: ambulance service access to some form of 'Alternative Care Pathway' (ACP) whereby those seeking help for an epileptic seizures judged not to require ED are cared for within less costly, alternative environments.

The exact nature of the ACP is not clear. Different regions and services are considering different configurations and doing so in an uncoordinated way. ACPs being considered include paramedics transporting patients' home or to an urgent treatment centre rather than ED. Others involve paramedics leaving patients at scene with the offer of a telephone call from a nurse or general practitioner with a specialist interest in epilepsy within 24, 48 or 72 hours. A possible benefit of this latter configuration is health inequalities could be reduced by PWE 'in need' being brought to specialists' attention.

ACPs are not new [46] and paramedics have not been obliged to transport patients to ED since ~1997. The ambitions of ACPs have though increased and there is significant support within the UK policy realm to expand the number of conditions covered by them as a means of managing demands on hospital services (e.g. [47-49]).

For peer review only - http://bmjopen.bmj.com/site/about/guidelines.xhtml

Evidence on the utility of ACPs is generally positive.[46 50-52] In their review of potential revisions to the urgent and emergency care system, the Nuffield Trust identified greater ambulance/paramedic triage in the community as having the most the positive evidence of effectiveness.[39] Paramedics appear willing and safely able to use ACPs when trained and for some ACPs there is evidence of cost-savings and greater patient satisfaction.[50 51]

Approach to ACP development concerning

An ACP for epilepsy holds potential. As a team of health care professionals, researchers and service user representatives with expertise in epilepsy and urgent care our ultimate goal is therefore to evaluate the most promising ACP and use the evidence to transform service organization nationally. However, we cannot currently do this because the way in which ACPs are being developed means it is not known which of the many ACPs has the potential to be most effective and could be justified for selection.

Specifically, ACPs are being developed largely 'top-down', with the patient voice being absent. The nature and content of ACPs may not therefore align with what patients/carers would consider to meet their needs. Evidence suggests decisions to access healthcare services can be informed by how a patient/carer perceives their situation [53-55] and there can be a mismatch between patients/carers and health professionals' views, including of what constitutes an 'emergency'.[56-59] The upshot is the acceptability – a fundamental criterion an intervention needs to satisfy to be positioned to achieve its intended outcome [60] – of the different ACPs to patients and carers is unknown.

To date, one epilepsy ACP has been reported on.[61] Less than 10% of eligible PWE attended to by paramedics were put onto it. The reasons for the low uptake rate were not explored. A possible explanation is the ACP was not acceptable to PWE.

It has been assumed the target population *does not* want to be conveyed to ED and will readily accept an ACP. It may not be that straightforward. Some PWE certainly express dissatisfaction with being taken to ED [62]. Others though express a need for immediate access to urgent care. They have explained how they and their family (to whom care decisions are often delegated when the patient is unconscious or lacks capacity) are often fearful of seizures, including the possibility of brain damage.[28 29]

CURRENT PROJECT

This 28-month project seeks identify the ACP that should be prioritized for testing/ implementation. It seeks to shape the change that results from the identified momentum so the likelihood of patients and the NHS benefiting are maximised and finite health resources used in an informed, rational way. It will bring patients/carers from the target population, health care professionals and commissioners together to identify which ACP configuration best

For peer review only - http://bmjopen.bmj.com/site/about/guidelines.xhtml

encapsulates the features important to patients and carers *and* is feasible within the NHS context.

The project has 3 linked stages and will use survey techniques, qualitative methods, consensus meetings and Discrete Choice Experiments (DCEs) to achieve its aims (Figure 1).[63 64] DCEs are an attribute-based survey methodology, underpinned by the theory that any 'good' (including a health service) can be described by its constituent characteristics (attributes), and that the extent to which an individual prefers a good depends on the levels these attributes take.[65] We considered DCEs to provide an efficient, scientifically defensible, and non-tokenistic way of bringing the patient voice into ACP design since they allow a person's stated preferences and priorities to be elicited by presenting them with a hypothetical scenario (e.g., having a seizure at home) and asking them to choose which of two (or more) care options described by a series of attributes they prefer. The process is then repeated with alternative care choices being presented. By varying attribute levels and observing how participants change responses, the importance of attributes and the extent to which they drive preference can be inferred.

Aims

a) Systematically identify ACPs being considered by the NHS for epilepsy and describe extent to which service users have been involved in their design.
b) Understand patient and carers' decision-making processes for seeking or not seeking ED care, and their concerns and expectations regarding ACPs.
c) Identify the attributes of post-seizure emergency care that PWE and their carers consider important and identify if this differs by the context in which the seizure occurs.
d) Determine patient and carer preferences for post-seizure emergency care using DCEs and identify any subgroup differences.
e) Estimate uptake of different ACPs configurations by patients and subgroup differences.
f) Hold expert workshops to develop an optimal ACP following seizures, to be prioritised for implementation and evaluation via a subsequent project.

METHODS

Stage 1

Stage 1a: Pathway survey

Purpose: Systematically identify ACPs being considered. Will allow ACPs presented within DCEs to include main ones being considered.

For peer review only - http://bmjopen.bmj.com/site/about/guidelines.xhtml

Design: Cross-sectional survey of NHS service providers asking respondents whether their service has considered (or implemented) any changes to the management of those with epilepsy/suspected seizures to minimise clinically unnecessary/avoidable ED visits. Respondents will report how change is anticipated to minimise visits and whether service users informed change.

The survey will take ~10 minutes, be piloted and completed online. Survey respondents will be able to indicate if they want to be informed of subsequent project parts.

Recruitment: Invitations to participate, followed by two reminders, will be sent to clinical leads/ directors of England's regional ambulance trusts (n=11), regional neuroscience (~n=25) [66] and neurology centres (~n=16) [67 68], and a random sample of 25% of its 'Type 1' EDs [69] (n=35), stratified by geographical area and size (attendances).

Analysis: Characteristics of responding and non-responding organisations will be compared. To determine the types of ACPs being considered and proportion of organisations considering each, a subset of surveys will be selected. A qualitative researcher, supported by the wider team, will read these to identify recurrent ACP types and collate them into a thematic coding framework. The framework will be applied to the full data set and modified to ensure all ACPs types are captured.

Stage 1b: Qualitative interviews

Purpose: Understand patient and carer decision-making, the important attributes of post-seizure care, and concerns and expectations regarding ACPs. Will permit attribute development to adhere to good practice.[70 71]

Design: Face-to-face semi-structured interviews will be conducted with PWE, and where possible an informal carer, and shaped by a piloted topic guide.

Participants will be asked for contextual information about their epilepsy and ED visits. Evaluative information will then be sought by respondents describing positive and negative perceptions of emergency care and any ACPs experienced. The researcher will explore the decision-making surrounding patients and carers seeking or not seeking ED care following recent seizures. Potential attributes shall be obtained by describing two known ACPs (i.e., urgent care centres and being taken home) and asking participants for their expectations and concerns.

To help reduce the number of care attributes and improve face validity, participants will rank what they consider to be the five most important care attributes. Interviewer will help recall those mentioned and write them onto show cards.

For peer review only - http://bmjopen.bmj.com/site/about/guidelines.xhtml

Interviews will be audio-recorded, transcribed verbatim and conducted and reported according to COREQ.[72]

Recruitment: Aim is to capture a comprehensive range of perspectives, and sample size will be determined by data saturation. We anticipate [70 73] recruiting a purposive sample of ~30 PWE, with or without an informal carer. Table 1 lists the inclusion and exclusion criteria.

Eligible PWE will be identified by two means. Firstly, persons with uncontrolled epilepsy who participated in a recent NIHR funded 'Self-Management Education for adults with Epilepsy' (SMILE) trial in the South-East of England and expressed interest in future projects will sent notification of the study.[74] Secondly, adverts will be circulated to persons affiliated with epilepsy user groups, including Epilepsy Action (EA). Persons interested in taking part will be asked to contact the research team. To identify carer participants, patients will be asked to choose a significant other/informal carer to take part. Participants will receive a £20 shopping voucher.

Analysis: Data collection and analyses will proceed in an iterative manner. An qualitative researcher, supported by the wider team, will analyse the data thematically [75] to generate an exhaustive list and description of the salient attributes of care (and levels) identified by participants.

Transcripts will be read and re-read, and coded line-by-line. Analysis will pay attention to the similarity and differences in salient attributes according to where the seizure occurred (home, public), who was there, who made the 999 call, whether the patient had seen a specialist in the prior 12 months, and whether they self-reported an intellectual impairment. NVivo 11 software will store data and support coding.[76]

The attributes nominated during the ranking exercise will be categorised according to the thematic framework, then analysed quantitatively by EH using standardised rank scores.

Stage 1c: Knowledge exchange event

Purpose: It is important the DCE elicits views on ACPs that could plausibly be delivered within the NHS. This event will therefore, early in the project, determine feasibility of the attributes and associated levels that have been identified by service users in Stage 1b.

Design: One-day event at which health care professional representatives will be shown draft attributes, levels, and choice sets and asked to deliberate each choice. For instance, should Stage 1b indicate service users consistently say they prefer to be transported home by the ambulance service regardless of distance, then representatives would be asked to what constitutes a feasible distance. Our team has previously used this approach.[77]

For peer review only - http://bmjopen.bmj.com/site/about/guidelines.xhtml

Recruitment: Around n=15 ambulance staff and neurology service representatives. Persons will be recruited from organisations participating in Stage 1a with the help of a sampling framework to ensure representatives come from geographically diverse areas. Representatives will be reimbursed for travel. Those not able to attend will be able to provide feedback remotely.

Analysis: With attendees' consent, the session will be audio-recorded and field notes taken. Feedback will be used to optimise the attribute levels within the DCEs.

For peer review only

Stage 2

Stage 2a: Developing the DCE

Purpose: Generate DCE questionnaire to elicit patient and carer preferences for post-seizure care and which meets best practice guidance.[70 78]

Design: A multidisciplinary panel, chaired by DH, will compose a draft DCE questionnaire using Stage 1 evidence and refine it on the basis of a pilot. The panel will include expertise in DCEs (EH), emergency medicine (SG), neurology (AM), paramedical science (MJ), general practice (JD), and psychology (AN), as well as having service user representation.

The panel will form a consensus on which restricted number of ACPs best represent the range being considered and are to be valued using the DCE. Using the list of candidate attributes generated from Stage 1b the panel will select 4-6 attributes by which to describe the packages. They will favour attributes prioritised by service users, which are modifiable, capable of being traded and best describe the ACPs.

DCEs will then be generated for the three most common presentations to ED by PWE. For each, participants will be presented with a seizure scenario vignette and via standardised instructions complete a series of forced, pairwise comparisons to indicate which care packages they would prefer. Vignettes will be developed by the panel based on clinical and lived-experience. Supplementary File 1 provides an illustration of what a DCE *might* look like.

Plausible attribute levels will be specified on the basis of findings from Stage 1, expert opinion and relevant literature. A strength of DCEs is the possibility of varying attribute levels to also model preferences within future contexts. Using its expertise and knowledge, the panel will seek to assign levels to attributes to account for major anticipated changes – such as longer ED waiting times [79].

A fractional factorial design will identify a purposeful subset of options for each DCE, to allow an estimate of the main effect of each attribute independently and selected two-factor interactions, while minimizing the number of paired comparisons participants are asked to

For peer review only - http://bmjopen.bmj.com/site/about/guidelines.xhtml

make. The profiles will be obtained from a published design catalogue.[80] Binary choices will then be created using the fold-over method, which replaces each attribute level systematically.[81]

Once drafted, the DCE will undergo iterative individual pilot testing with ~15 PWEs and carers from the project's Patient and Public Involvement (PPI) group to check clarity and duration. Pilotees will 'think aloud' [82] when making choices, and asked to consider their preferred presentation of attributes (e.g. text, pictograms). Sessions will be audio recorded, notes made and refinements made.

Stage 2b: Formal DCE

Purpose: Determine patient and carer preferences for post-seizure emergency care, estimate uptake of different ACPs configurations, and subgroup differences.

Design: A representative sample of PWE and informal carers who have visited ED in the last 12 months, as well as those at risk, will be sought to complete the finalised DCE questionnaire. PWE who have uncontrolled epilepsy, but who have not visited ED in the last 12 months will be asked to visit an online survey page and complete the DCE questionnaire using their own internet enabled devices. Due to potentially greater social disadvantage, patients who *have* visited ED in the prior 12 months will have the additional option of completing the DCE during a face-to-face appointment with a researcher who will provide an internet enabled device.

To limit burden, participants will complete DCEs relating to just two seizure scenarios (allocated at random). As well as the DCEs, respondents will report on their demographics, epilepsy, knowledge and fear of seizures, and service use. Each participant will receive a £20 shopping voucher.

Recruitment: General Practices in North-West England will identify potential patient participants by searching their epilepsy registers.[83] Practices will be recruited with the help of the NIHR Clinical Research Network North West Coast. Practices will send ostensibly eligible patients invitation packs via 'Docmail' and a repeat invite 2 weeks later to those not responding. Patients not wishing to participate will be encouraged to notify the team and detail their reasons. Eligibility criteria are in Table 1.

A definitive sample size calculation depends on the finalized design of the DCE.[84] To permit decisions regarding recruitment to be made in the meantime we anticipated the design and calculated the required sample size by using Orme's[85] formula. Full details are provided in Table 2. This indicated 348 PWE (or associated informal carers) would be required, comprising data 174 patients (or associated carers) who have visited ED in the prior 12 months and 174 patients (or associated carers) who have not.

For peer review only - http://bmjopen.bmj.com/site/about/guidelines.xhtml

Based on responses to prior health DCEs [86] and studies with PWE,[16 27] we anticipate 30-60% of those invited will agree to participate. An average English GP practice [155] will have 24 PWE aged ≥18 on their register who have experienced ≥1 seizure in the prior 12 months,[9] with 10 having visited an ED during that period. [12-14] Governed by the need to recruit a sub-group of 174 PWE who have visited ED in the prior 12 months, 29-58 general practices will be required.

Analysis: Data for the different seizure vignettes will be analysed separately using random effects logit regression models. This will determine the importance of the different care attributes for preference and direction of effect. Analyses will be completed with and without respondents failing a 'rationality' test.

The influence of participants' characteristics on preference, including whether they had visited an ED in the prior 12 months, whether they were a patient or carer, social deprivation, and presence of an intellectual impairment, will be examined as a secondary analysis.

Marginal rates of substitution (the rate at which respondents are willing to give up a unit change in one attribute in exchange for a unit change in another while maintaining the same level of utility) will be calculated. 'Utility scores' for different ACP configurations will be calculated and ranked accordingly. Their uptake under different seizure scenarios will be estimated.

Stage 3

Stage 3a Knowledge Exchange workshops

Purpose: Disseminate Stage 1 and 2 findings to those positioned to develop, fund, support and run ACPs and utilise their expertise to identify which ACP/s favoured by patients and carers is most NHS feasible and should be prioritised for implementation/ evaluation.

Design: One Knowledge Exchange event in South England, one in the North. They will start with presentation of project's findings, including ACP configurations ranked according to service user preference. A Nominal Group Technique, described by the James Lind Alliance [87], will then be used to hear delegates' views of the ACPs and rank them according to the extent to which they meet users' preferences and are NHS feasible. Facilitators will encourage delegates to consider barriers to change, supply constraints, acceptability of the service to staff, possible cost, and potential to redress health care inequalities.

Voting will be completed using electronic devices. With attendees' consent, sessions will be audio-recorded and a qualitative researcher will observe and record field notes.

For peer review only - http://bmjopen.bmj.com/site/about/guidelines.xhtml

Recruitment: Aim to have 40-50 health service managers, commissioners, healthcare professionals, and service user representatives at each event. Persons will be recruited mainly from the institutions participating in Stage 1a. Additional attendees will come from commissioning groups and the epilepsy patient groups. The project's PPI group will also be invited. A sampling matrix will be formed to help ensure broadly equal representation of persons from health professional, commissioning and managerial roles.

Analysis: The rank order of the ACPs that results from delegates voting will be described. Depending on findings from the previous stages, the ACPs might have been presented to delegates according to seizure scenario. If so, rank orders for the ACPs for the different scenarios will be determined and compared. As a secondary objective, rank orders for the different representative subgroups will be determined. Transcripts from the events will be thematically analysed to capture delegates' views and justifications for their preferences.

DISCUSSION

Patient and public involvement

This research was driven by a need to consider the views of PWE when designing an ACP for epilepsy. EA, the largest user organisation in the UK, contributed to the design of the research, and facilitated a PPI event with 23 service users to discuss project design and conduct. A PPI group will be established, including PWE and carers. They will be supported by EA who have an active PPI scheme and reimbursed for travel and time according to INVOLVE.[88] The group will contribute as research peers by: providing feedback on research materials; interpretation of findings; piloting the DCE; and being delegates in workshops. The group will be encouraged to help with dissemination, including presenting the findings at the Stage 3 events and teaching.

Ethics and oversight

Monitoring by an independent Study Steering Committee (SSC) will help to ensure the rights, safety and well-being of participants are the most important considerations. Only persons providing informed consent will participate. The SSC will be composed according to our funder guidelines.[89] Compliance with the principles of Good Clinical Practice and scientific integrity will be managed by the study management team through regular and ad hoc meetings.

Dissemination

The project will identify what, if any, ACP configuration/s is most acceptable to service users and has the backing of those expected to deliver and support it. To ensure maximum impact those considering an ACP need to be aware of the project, have confidence in its outputs and

For peer review only - http://bmjopen.bmj.com/site/about/guidelines.xhtml

use its findings. To help ensure this: 1) Representatives from all of the relevant service providers in the UK will be informed of the project (including notifying the leads of England's Sustainability and Transformation Partnerships and Urgent and Emergency Care Vanguards); 2) professional bodies will announce its funding (Acknowledgements); 3) the research team includes appropriate expertise and opinion leaders; and 4) representatives from relevant NHS services will be invited to take part in Stage 1a and 3a.

The project's findings will be disseminated via international conferences and at least four journal manuscripts. The manuscripts will be published open access and non-technical reports will be circulated via NHS network newsletters and Epilepsy Action. Participants will be provided with a copy of the results.

Having identified the ACP, funding will stop but the team would, via a subsequent project seek to implement and evaluate the chosen ACP. The nature of that evaluation will be informed by the state of the NHS closer to the time. The Medical Research Council [90] highlight intervention evaluation takes place in a wide range of settings and many factors can constrain choice of evaluation methods. One trade-off that may need consideration is time until results become available/ demand versus methodological rigour.

For peer review only

For peer review only - http://bmjopen.bmj.com/site/about/guidelines.xhtml

Acknowledgements: The authors would like to thank Epilepsy Action (Angie Pullen, Amanda Stoneman) for the role that they will have in this project. They would like to express appreciation for the contributions from people with epilepsy and their family members and friends who will participate in this study and thank members of the Study Steering Committee (Tom Quinn [chair], Verity Watson, Trevor Baldwin, Nigel Rees, Khalid Hamandi, Daniel Horner, Juliet Bransgrove, and Jayne and Sam Burton). We also acknowledge the International League Against Epilepsy (British Branch), Royal College of Emergency Medicine and the Epilepsy Nurses Association for informing members of the project. The study sponsor is the University of Liverpool (reference: UoL4258; sponsor@liv.ac.uk).

Author Contributions: AJN conceived of the study and designed it together with EH, DH, LR, MM, MJ, SG and AGM. EH planned the statistical analysis for the DCE. AJN and AM wrote the manuscript, with revisions being made by EH, DH, LR, MM, MJ, SG and AGM. All authors read and approved the final manuscript.

Funding statement: This project is funded by the National Institute for Health Research's Health Services and Delivery Research Programme (HS&DR Programme) (project number 17/05/62). The views and opinions expressed herein are those of the authors and do not necessarily reflect those of the University of Liverpool, the HS&DR programme, the NIHR, the NHS, or the Department of Health and Social Care.

Competing interests: None declared.

Patient consent for publication: Not required.

Data statement: This is a protocol. There is no data associated with it.

Ethics approval: This study has been received Health Research Authority approval and favourable ethical opinions from NRES Committee West Midlands - Solihull (19/WM/0012) and King's College London's Psychiatry, Nursing and Midwifery Ethics Committee (LRS-18/19-10353).

For peer review only

For peer review only - http://bmjopen.bmj.com/site/about/guidelines.xhtml

Table 1 Participant inclusion and exclusion criteria

Study part	Inclusion criteria	Exclusion criteria
**Stage 1b – Qualitative interviews**		
	▪ Established diagnosis of epilepsy, or an informal carer for someone with epilepsy<br>▪ Age ≥18 years (no upper age limit)<br>▪ Have visited ED in the past 12 months for epilepsy (as reported by the patient)*<br>▪ Able to provide informed consent and communicate in English	▪ Severe current psychiatric disorders (e.g. acute psychosis)<br>▪ Life-threatening medical illness
**Stage 2 – Discrete Choice Experiments**		
**Group 1**		
**Patients**	▪ Clinically confirmed diagnosis of epilepsy (for any duration)<br>▪ Any epilepsy syndrome and any types of focal or generalised seizures<br>▪ Currently being prescribed antiepileptic medication<br>▪ Age ≥18 years (no upper age limit)<br>▪ Have visited ED in the past 12 months for epilepsy<br>▪ Able to provide informed consent and independently complete a questionnaire in English<br>▪ Lives in the North West of England	▪ Severe current psychiatric disorders (e.g. acute psychosis)<br>▪ Life-threatening medical illness<br>▪ Resides within a care or nursing home or of no fixed abode
**Carers**	▪ A significant other to a person with epilepsy (e.g. family member,	▪ Severe current psychiatric disorders (e.g. acute psychosis)

For peer review only - http://bmjopen.bmj.com/site/about/guidelines.xhtml

	friend) who the patient identifies as providing informal support or self-identifies themselves if the patient has a substantial intellectual disability<br>▪ The person with epilepsy they care for has visited ED in the past 12 months<br>▪ Age ≥16 years (no upper age limit)<br>▪ Able to provide informed consent and independently complete a questionnaire in English<br>▪ Lives in the North West of England	▪ Life-threatening medical illness
**Group 2**		
**Patients**	▪ Clinically confirmed diagnosis of epilepsy (for any duration)<br>▪ Any epilepsy syndrome and any types of focal or generalised seizures<br>▪ Currently being prescribed antiepileptic medication<br>▪ Age ≥18 years (no upper age limit)<br>▪ Has *not* visited ED in the past 12 months, but has had a seizure with loss of awareness in this period<br>▪ Able to provide informed consent and independently complete a questionnaire in English	▪ Severe current psychiatric disorders (e.g. acute psychosis)<br>▪ Life-threatening medical illness<br>▪ Resides within a care or nursing home or has no fixed abode
**Carers**	▪ Lives in the North West of England<br>▪ A significant other to a person with epilepsy (e.g. family member, friend) who the patient identifies as providing informal support or self-identifies themselves if the patient	▪ Severe current psychiatric disorders (e.g. acute psychosis<br>▪ Life-threatening medical illness

For peer review only - http://bmjopen.bmj.com/site/about/guidelines.xhtml

has a substantial intellectual disability

- The person with epilepsy they care for has *not* visited ED in the past 12 months, but had a seizure with loss of awareness in this period
- Age ≥16 years (no upper age limit)
- Able to provide informed consent and independently complete a questionnaire in English
- Lives in the North West of England

Notes: ED, emergency department; *Should recruitment prove slower than anticipated, the recruitment criteria will be relaxed to allow people who have had ED or ambulance contact within the last 2 years.

For peer review only

For peer review only - http://bmjopen.bmj.com/site/about/guidelines.xhtml

Table 2 Sample size calculation for Discrete Choice Experiments

**Formula used and factors considered in estimating likely sample size required**
• Orme's formulae: (*n***t***a*)/ *c* must be ≥500, where *n* is number of respondents, *t* number of tasks, *a* number of alternatives per task, and *c* number of analysis cells (for main effects, the largest number of levels for any one attribute).<br>• Anticipated Discrete Choice Experiments (DCEs) to involve participants being asked to make (*t*) 9 separate choices between (*a*) 2 care packages and (*c*) 3 would be maximum attribute levels. Thus, Orme's formula indicated complete data from n=84 needed.<br>• Intend to complete DCEs for three different seizure scenario, but participants would only complete two, therefore 126 participants required.<br>• To allow for variation in the finalized number of choice tasks and levels we increased this to 150 and further inflated it to 174 to accommodate for ~15% of missing data experienced by prior epilepsy DCEs.<br>• As an objective was to describe and compare care preferences of two subgroups (patients (and associated carers) who have visited an ED in the prior 12 months vs. patients (and associated carers) who have had a seizure in the prior 12 months, but have not visited an ED), 174 patients (and associated carers) who have visited ED in the prior 12 months and 174 patients (and associated carers) who have not, will need to be recruited.<br>• Total sample size, thus n=348.

For peer review only

Figure Legend

Figure 1: Research project process diagram.

Notes to Figure 1: HRA, Health Research Authority; EDs, Emergency Departments; PWE, People with Epilepsy; DCEs, Discrete Choice Experiments; ACP/s, Alternative Care pathway/s

For peer review only

For peer review only - http://bmjopen.bmj.com/site/about/guidelines.xhtml

REFERENCES

1. NHS England. Five Year Forward View 2014 [Available from: https://www.england.nhs.uk/wp-content/uploads/2014/10/5yfv-web.pdf.
2. NHS England. Next Step on the NHS Five Year Forward View 2017 [Available from: england.nhs.uk/wp-content/uploads/2017/03/NEXT-STEPS-ON-THE-NHS-FIVE-YEARFORWARD-VIEW.pdf.
3. Ridsdale L, Charlton J, Ashworth M, et al. Epilepsy mortality and risk factors for death in epilepsy: a population-based study. *Br J Gen Pract* 2011;61(586):e271-e78.
4. Hart YM, Shorvon SD. The nature of epilepsy in the general population. II. Medical care. *Epilepsy research* 1995;21(1):51-58.
5. Jacoby A, Buck D, Baker G, et al. Uptake and costs of care for epilepsy: findings from a UK regional study. *Epilepsia* 1998;39(7):776-86.
6. Kitson A, Shorvon S. Services for patients with epilepsy: Report of a CSAG committee: Department of Health, 2000.
7. Noble AJ, Goldstein LH, Seed P, et al. Characteristics of people with epilepsy who attend emergency departments: Prospective study of metropolitan hospital attendees. *Epilepsia* 2012;53(10):1820–28.
8. Bautista RED, Glen ET, Wludyka PS, et al. Factors associated with utilization of healthcare resources among epilepsy patients. *Epilepsy research* 2008;79(2-3):120-29.
9. Allard J, Shankar R, Henley W, et al. Frequency and factors associated with emergency department attendance for people with epilepsy in a rural UK population. *Epilepsy & Behavior* 2017;68:192-95.
10. Public Health England. The NHS Atlas of Variation in Healthcare: Reducing unwarranted variation to increase value and improve quality. 2016 [Available from: fingertips.phe.org.uk/documents/Atlas_2015%20Compendium.pdf.
11. QIPP/Right Care. The NHS atlas of variation in healthcare 2010 [Available from: fingertips.phe.org.uk/documents/Atlas_2010%20Compendium.pdf.
12. Right Care. The NHS Atlas of Variation in Healthcare: Reducing unwarranted variation to increase value and improve quality. 2011
13. Pearson M, Marson A, Dixon P, et al. National Audit of Seizure Management in Hospitals: St. Elsewhere's Hospital Clinical Report 2014 [Available from: http://www.nashstudy.org.uk/Newsletters/St%20Elsewhere's%20Clinical%20Report%20NASH%202.pdf.
14. Pearson M, Marson A, Dixon P, et al. National Audit of Seizure Management in Hospitals St Elsewhere's Clinical Report 2012 [Available from: http://nashstudy.org.uk/Newsletters/St%20Elsewhere%27s%20NASH%20Report.pdf.
15. House of Commons Committee of Public Accounts. Services to people with neurological conditions: progress review London House of Commons; 2015 [Available from: https://publications.parliament.uk/pa/cm201516/cmselect/cmpubacc/502/502.pdf accessed 4th April 2019.
16. Ridsdale L, McCrone P, Morgan M, et al. Can an epilepsy nurse specialist-led self-management intervention reduce attendance at emergency departments and promote well-being for people with severe epilepsy? A non-randomised trial with a nested qualitative phase. *Health Services and Delivery Research* 2013;1(9)
17. Dickson JM, Jacques R, Reuber M, et al. Emergency hospital care for adults with suspected seizures in the NHS in England 2007–2013: a cross-sectional study. *BMJ open* 2018;8(10):e023352.

For peer review only - http://bmjopen.bmj.com/site/about/guidelines.xhtml

18. Bardsley M, Blunt I, Davies S, et al. Is secondary preventive care improving? Observational study of 10-year trends in emergency admissions for conditions amenable to ambulatory care. *BMJ open* 2013;3(1):e002007.
19. Peter A Dixon, Jamie J Kirkham, Anthony G Marson, et al. National Audit of Seizure management in Hospitals (NASH): results of the national audit of adult epilepsy in the UK *BMJ Open* 2015;5(3):e007325.
20. Reuber M, Hattingh L, Goulding PJ. Epileptological emergencies in Accident and Emergency: a survey at St James's University Hospital, Leeds. *Seizure* 2000;9(3):216-20.
21. Ryan J, Nash S, Lyndon J. Epilepsy in the accident and emergency department--developing a code of safe practice for adult patients. South East and South West Thames Accident and Emergency Specialty Sub-committees. . *Emergency Medicine Journal* 1998;15
22. Whiston S, Coyle B, Chappel D. Health needs assessment for long term neurological conditions in North East England. Stockton on Tees: North East Public Health Observatory 2009.
23. NICE. Services for the diagnosis and management of the epilepsies in adults, children and young people: commissioning guide 2013 [Available from: nice.org.uk/guidance/qs26/resources/services-for-thediagnosis-and-management-of-the-epilepsies-in-adults-children-and-young-people-commissioning-guide-317820637/chapter/3-Assessingservice-levels-for-the-diagnosis-and-management-of-the-epilepsies#ftn.footnote_19.
24. Dixon PA, Kirkham JJ, Marson AG, et al. National Audit of Seizure management in Hospitals (NASH): results of the national audit of adult epilepsy in the UK. *BMJ Open* 2015;5:e007325.
25. Thornton S. Beyond rhetoric: we need a strategy for patient involvement in the health service. *Bmj* 2014;348:g4072.
26. House of Commons Committee of Public Accounts. Financial sustainability of the NHS: 43 2016–17, HC 887 2017 2017 [Available from: publications.parliament.uk/pa/cm201617/cmselect/cmpubacc/887/887.pdf.
27. Noble AJ, Marson AG, Tudur-Smith C, et al. 'Seizure First Aid Training' for people with epilepsy who attend emergency departments, and their family and friends: study protocol for intervention development and a pilot randomised controlled trial. *BMJ Open* 2015;5:e009040.
28. Noble AJ, Morgan M, Virdi C, et al. A nurse-led self-management intervention for people who attend emergency departments with epilepsy: the patients' view. *Journal of Neurology* 2013;260(4):1022–30.
29. Ridsdale L, Virdi C, Noble AJ, et al. Explanations given by people with epilepsy for using emergency medical services: a qualitative study. *Epilepsy Behaviour* 2012;25:529–33.
30. Ridsdale L, Kwan I, Cryer C. The effect of a special nurse on patients' knowledge of epilepsy and their emotional state. Epilepsy Evaluation Care Group. *Br J Gen Pract* 1999;49(441):285-89.
31. Ridsdale L, Kwan I, Cryer C, et al. Newly diagnosed epilepsy: Can nurse specialists help? A randomized controlled trial. *Epilepsia* 2000;41(8):1014-19.
32. Grainger R, Pearson M, Dixon P, et al. Referral patterns after a seizure admission in an English region: an opportunity for effective intervention? An observational study of routine hospital data. *BMJ open* 2016;6(1):e010100.
33. Morrish PK. Inadequate neurology services undermine patient care in the UK. *Bmj* 2015;350:h3284.
34. Moran N, Poole K, Bell G, et al. Epilepsy in the United Kingdom: seizure frequency and severity, anti-epileptic drug utilization and impact on life in 1652 people with epilepsy. *Seizure* 2004;13(6):425-33.
35. Ashworth M, Seed P, Armstrong D, et al. The relationship between social deprivation and the quality of primary care: a national survey using indicators from the UK Quality and Outcomes Framework. *Br J Gen Pract* 2007;57(539):441-48.

For peer review only - http://bmjopen.bmj.com/site/about/guidelines.xhtml

36. Alderwick H, Dunn P, McKenna H, et al. Sustainability and transformation plans in the NHS: How are they being developed in practice? : The King's Fund; 2016 [Available from: https://www.kingsfund.org.uk/sites/default/files/field/field_publication_file/STPs_in_NHS_Kings_Fund_Nov_2016.pdf.
37. NICE. Epilepsy in adults: Briefing Paper: NICE; 2012 [Available from: nice.org.uk/guidance/qs26/documents/epilepsy-in-adults-briefing-paper2.
38. National Clinical Guideline Centre. Pharmacological Update of Clinical Guideline 20. The Epilepsies: The Diagnosis and Management of the Epilepsies in Adults and Children in Primary and Secondary Care London: National Clinical Guideline Centre (UK); 2012 [Available from: ncbi.nlm.nih.gov/pubmedhealth/PMH0068980.
39. Imison C, Curry N, Holder H, et al. Shifting the balance of care: great expectations. Research Report: Nuffield Trust; 2017 [Available from: https://www.nuffieldtrust.org.uk/files/2017-02/shifting-the-balance-of-care-report-web-final.pdf.
40. Day J. Care Given to Patients Treated for Epileptic Seizure by the London Ambulance Service. London: London Ambulance Service Clinical Audit and Research Unit 2011.
41. Dickson JM, Taylor LH, Shewan J, et al. Cross-sectional study of the prehospital management of adult patients with a suspected seizure (EPIC1). *BMJ Open* 2016;6:e010573.
42. Marks P, Daniel T, Afolabi O, et al. Emergency (999) calls to the ambulance service that do not result in the patient being transported to hospital: an epidemiological study. *Emergency Medicine Journal* 2002;19:449–52.
43. Burrell L, Noble A, Ridsdale L. Decision-making by ambulance clinicians in London when managing patients with epilepsy: a qualitative study. *Emergency Medicine Journal* 2013;30:236–40.
44. Noble A, Snape D, Goodacre S, et al. Qualitative study of paramedics' experiences of managing seizures: a national perspective from England. *BMJ Open* 2016;6:e014022.
45. Sherratt F, Snape D, Goodacre S, et al. Paramedics' views on their seizure management learning needs: a qualitative study in England. *BMJ Open* 2017;7:e014024.
46. Snooks H, Kearsley N, Dale J, et al. Towards primary care for non-serious 999 callers: results of a controlled study of "Treat and Refer" protocols for ambulance crews. *Quality and Safefy in Health Care* 2004;13:435–43.
47. House of Commons Health Committee. Winter pressure in accident and emergency departments, 2016 HC 277 2016 [Available from: publications.parliament.uk/pa/cm201617/cmselect/cmhealth/277/277.pdf.
48. NHS. The NHS Long Term Plan 2019 [Available from: https://www.longtermplan.nhs.uk/ accessed 14 May 2019.
49. NHS England. Transforming urgent and emergency care services in England: Clinical models for ambulance services. Urgent and Emergency Care Review Programme Team. 2015 [Available from: nhs.uk/NHSEngland/keogh-review/Documents/UECR-ambulance-guidance-FV.PDF.
50. Fisher JD, Freeman K, Clarke A, et al. Patient safety in ambulance services: a scoping review. 2015
51. Turner J, Coster J, Chambers D, et al. What evidence is there on the effectiveness of different models of delivering urgent care? A rapid review. 2015
52. Health & Social Care Information Centre. Ambulance Services, England 2014-15. 2015 [Available from: digital.nhs.uk/catalogue/PUB17722.
53. Frostholm L, Fink P, Christensen K, et al. The patients' illness perceptions and the use of primary health care. *Psychosomatic Medicine* 2005;67(6):997-1005.
54. Lowe R, Porter A, Snooks H, et al. The association between illness representation profiles and use of unscheduled urgent and emergency health care services. *British Journal of Health Psychology* 2011;16(4):862-79.
55. Martin R, Leventhal H. Symptom perception and health care-seeking behavior. . In: Raczynski J, Leviton L, eds. Handbook of clinical health psychology: Vol 2 Disorders of behavior and health. Washington, DC, US: American Psychological Association. 2004:299-328.

For peer review only - http://bmjopen.bmj.com/site/about/guidelines.xhtml

56. Ross J, Stefan H, Schäuble B, et al. European survey of the level of satisfaction of patients and physicians in the management of epilepsy in general practice. *Epilepsy & Behaviour* 2010;19(1):36-42.
57. Booker M, Purdy S, Shaw A. Seeking ambulance treatment for 'primary care' problems: a qualitative systematic review of patient, carer and professional perspectives. *BMJ Open* 2017;7:e016832.
58. Whitehead K, Kandler R, Reuber M. Patients' and neurologists' perception of epilepsy and psychogenic nonepileptic seizures. *Epilepsia* 2013;54(4):708–17.
59. Thompson R, Linehan C, Glynn M, et al. A qualitative study of carers' and professionals' views on the management of people with intellectual disability and epilepsy: A neglected population. *Epilepsy & Behaviour* 2013;28(3):379-85.
60. Michie S, West R, Campbell R, et al. ABC of Behaviour Change Theories. UK: Silverback Publishing 2014.
61. Dickson JM, Rawlings GH, Grünewald RA, et al. An alternative care pathway for suspected seizures in pre-hospital care: a service evaluation. *British Paramedic Journal* 2017;2(2):22-28.
62. Peterson CL, Walker C, Coleman H. 'I hate wasting the hospital's time': Experiences of emergency department admissions of Australian people with epilepsy. *Epilepsy & Behavior* 2019;90:228-32.
63. de Bekker-Grob EW, Ryan M, Gerard K. Discrete choice experiments in health economics: a review of the literature. *Health economics* 2012;21(2):145-72.
64. Clark MD, Determann D, Petrou S, et al. Discrete choice experiments in health economics: a review of the literature. *Pharmacoeconomics* 2014;32(9):883-902.
65. Lancaster KJ. A new approach to consumer theory. *Journal of political economy* 1966;74(2):132-57.
66. NHS England. D04 Neurosciences 2017 [Available from: https://www.england.nhs.uk/wp-content/uploads/2013/06/d04-neurosci-spec-neuro.pdf.
67. Association of British Neurologists. Acute Neurology services survey 2017. 2017 [Available from: https://www.theabn.org/media/Documents/Acute%20Neurology/ABN%20acute%20neurology%20survey%20final%2013%20March%202017.pdf
68. Brain and Spine Foundation. List of Neurocentres in the UK. [Available from: https://www.brainandspine.org.uk/wp-content/uploads/2018/03/BSF_List-of-Neurocentres-in-the-UK.pdf accessed 25th April 2019.
69. Health and Social Care Information Centre. Summary Report 7, Hospital Accident and Emergency Activity, 2016-17 2017 [Available from: digital.nhs.uk/media/33252/Hospital-Accident-and-Emergency-Activity-2016-17-Tables/default/acci-emer-atte-eng-2016-17-data.
70. Coast J, Al-Janabi H, Sutton EJ, et al. Using qualitative methods for attribute development for discrete choice experiments: issues and recommendations. *Health economics* 2012;21(6):730-41.
71. Vass C, Rigby D, Payne K. The role of qualitative research methods in discrete choice experiments: a systematic review and survey of authors. *Medical Decision Making* 2017;37(3):298-313.
72. Tong A, Sainsbury P, Craig J. Consolidated criteria for reporting qualitative research (COREQ): A 32-item checklist for interviews and focus groups. *International Journal for Quality in Health Care* 2007;19(6):349-57.
73. Powell G, Holmes EA, Plumpton CO, et al. Pharmacogenetic testing prior to carbamazepine treatment of epilepsy: patients' and physicians' preferences for testing and service delivery. *British journal of clinical pharmacology* 2015;80(5):1149-59.
74. Kralj-Hans I, Goldstein LH, Noble AJ, et al. Self-Management education for adults with poorly controlled epILEpsy (SMILE (UK)): a randomised controlled trial protocol. *BMC neurology* 2014;14(1):69.

For peer review only - http://bmjopen.bmj.com/site/about/guidelines.xhtml

75. Braun V, Clarke V. Using thematic analysis in psychology. *Qualitative research in psychology* 2006;3(2):77-101.
76. Spencer L, Ritchie J, O'Connor W, et al. Analysis in Practice. . In: Ritchie J, Lewis J, McNaughton Nicholls C, et al., eds. Qualitative Research Practice: A Guide for Social Science Students & Researchers. 2nd Edition ed. London: Sage 2014.
77. Epilepsy Action. A Critical Time for epilepsy in England. A study of epilepsy service provision in England Leeds: British Epilepsy Association 2013.
78. Bridges JF, Hauber AB, Marshall D, et al. Conjoint analysis applications in health—a checklist: a report of the ISPOR Good Research Practices for Conjoint Analysis Task Force. *Value in health* 2011;14(4):403-13.
79. House of Commons Hansard. Mental Health and NHS Performance, 9 January 2017, 619. 2017 [Available from: hansard.parliament.uk/commons/2017-01-09/debates/A0C1CB51-3E77-4FD0-87D9AD36C2C11CE5/MentalHealthAndNHSPerformance.
80. Hahn GJ, Shapiro SS. A cataloging and computer program for the design and analysis of orthogonal symmetric and asymmetric fractional factorial experiments. Schenectady, NY: General Electric Research and Development Center 1966: Report No. 66-C-165.
81. Street DJ, Burgess L. The construction of optimal stated choice experiments: Theory and methods. London: John Wiley & Sons 2001.
82. Ericsson KA, Simon HA. Protocol analysis: Verbal reports as data: the MIT Press 1984.
83. Health and Social Care Information Centre. Quality and Outcomes Framework – Prevalence, Achievements and Exceptions Report, England, 2016-17. 2017 [Available from: https://files.digital.nhs.uk/publication/c/r/qof-1617-rep.pdf accessed 9th April 2019.
84. Lancsar E, Louviere J. Conducting discrete choice experiments to inform healthcare decision making: a user's guide. *Pharmacoeconomics* 2008;26(8):661-77.
85. Orme B. Interpreting the results of conjoint analysis, getting started with conjoint analysis: strategies for product design and pricing research. Madison, Wis: Research Publishers LLC 2010.
86. Watson V, Becker F, de Bekker-Grob E. Discrete choice experiment response rates: A meta-analysis. *Health economics* 2017;26(6):810-17.
87. James Lind Alliance. JLA Guidebook 2019 [Available from: http://www.jla.nihr.ac.uk/jla-guidebook/ accessed 25th April 2019.
88. INVOLVE. Briefing notes for researchers: involving the public in NHS, public health and social care research Eastleigh: INVOLVE; 2012 [Available from: http://www.invo.org.uk/wp-content/uploads/2012/04/INVOLVEBriefingNotesApr2012.pdf
89. NIHR. TSC SSC Guidance 2016 [Available from: https://www.nihr.ac.uk/funding-and-support/documents/funding-for-research-studies/how-to-apply/TSC-SSC-Guidance-April-2016.pdf.
90. Craig P, Dieppe P, Macintyre S, et al. Developing and evaluating complex interventions: the new Medical Research Council guidance. *Bmj* 2008;337:a1655.

For peer review only

For peer review only - http://bmjopen.bmj.com/site/about/guidelines.xhtml

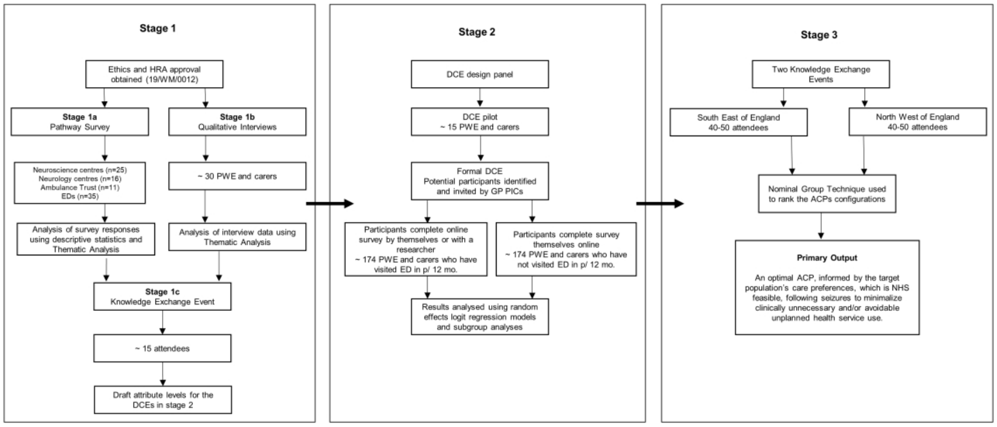

Figure 1 Research project process diagram

Notes: HRA, Health Research Authority; EDs, Emergency Departments; PWE, People with Epilepsy; DCEs, Discrete Choice Experiments; ACP/s, Alternative Care pathway/s

338x190mm (300 x 300 DPI)

BMJ Open

Developing patient-centred, feasible alternative care for adult emergency department users with epilepsy: Protocol for the mixed-methods observational 'Collaborate' project.

Journal:	*BMJ Open*
Manuscript ID	bmjopen-2019-031696.R1
Article Type:	Protocol
Date Submitted by the Author:	25-Jul-2019
Complete List of Authors:	Noble, Adam; University of Liverpool, Department of Health Services Research<br>Mathieson, Amy; University of Liverpool, Department of Health Services Research<br>Ridsdale, Leone; King's College London, Department of Basic and Clinical Neuroscience, Institute of Psychiatry, Psychology & Neuroscience<br>Holmes, EA; Centre for Health Economics & Medicines Evaluation<br>Morgan, Myfanwy; King's College London, Institute of Pharmaceutical Science<br>McKinlay, Alison; King's College London, Basic & Clinical Neuroscience<br>Dickson, Jon; The University of Sheffield, Academic Unit of Primary Medical Care<br>Jackson, Mike; North West Ambulance Service NHS Trust<br>Hughes, Dyfrig; Bangor University, Centre for Health Economics and Medicines Evaluation<br>Goodacre, Steve; University of Sheffield, Medical Care Research Unit<br>Marson, Anthony; University of Liverpool, Department of Molecular and Clinical Pharmacology
**Primary Subject Heading**:	Neurology
Secondary Subject Heading:	Emergency medicine, Health economics, Health services research, Qualitative research
Keywords:	Epilepsy < NEUROLOGY, ACCIDENT & EMERGENCY MEDICINE, Organisation of health services < HEALTH SERVICES ADMINISTRATION & MANAGEMENT, HEALTH ECONOMICS, QUALITATIVE RESEARCH

SCHOLARONE™
Manuscripts

TITLE: Developing patient-centred, feasible alternative care for adult emergency department users with epilepsy: Protocol for the mixed-methods observational 'Collaborate' project.

AUTHORS: Adam J. Noble,[1] Amy Mathieson,[1] Leone Ridsdale,[2] Emily Holmes,[3] Myfanwy Morgan,[4] Alison McKinlay,[2] Jon Dickson,[5] Mike Jackson,[6] Dyfrig Hughes,[3] Steve Goodacre,[7] Anthony G. Marson.[8]

AFFILIATIONS:

[1] Department of Health Services Research, University of Liverpool, UK.

[2] Department of Basic and Clinical Neuroscience, King's College London, UK.

[3] Centre for Health Economics & Medicines Evaluation, Bangor University, UK.

[4] Institute of Pharmaceutical Science, King's College London, UK.

[5] Academic Unit of Primary Medical Care, University of Sheffield, UK.

[6] North West Ambulance Service NHS Trust, Bolton, UK.

[7] School of Health and Related Research, University of Sheffield, UK.

[8] Department of Molecular and Clinical Pharmacology, University of Liverpool. UK.

FOR CORRESPONDENCE: Name: Adam Noble PhD; Address: University of Liverpool, Department of Health Services Research, Waterhouse Building, Liverpool, L69 3GB, United Kingdom.; Telephone: +44 151 794 5993; E-mail: adam.noble@liverpool.ac.uk

ABSTRACT

Introduction

Emergency department (ED) visits for epilepsy are common, costly, often clinically unnecessary and typically lead to little benefit for epilepsy management. An 'Alternative Care Pathway' (ACP) for epilepsy, which diverts people with epilepsy (PWE) away from ED when '999' is called and leads to care elsewhere, might generate savings and facilitate improved ambulatory care. It is unknown though what features it should incorporate to make it acceptable to persons from this particularly vulnerable target population. It also needs to be National Health Service (NHS) feasible. This project seeks to identify the optimal ACP configuration.

Methods and analysis

Mixed-methods project comprising 3-linked stages. In Stage 1, NHS bodies will be surveyed on ACPs they are considering and semi-structured interviews with PWE and their carers will explore attributes of care important to them and their concerns and expectations regarding ACPs. In Stage 2, Discrete Choice Experiments (DCE) will be completed with PWE and carers

For peer review only - http://bmjopen.bmj.com/site/about/guidelines.xhtml

to identify the relative importance placed on different care attributes under common seizure scenarios and the trade-offs people are willing to make. The uptake of different ACP configurations will be estimated. In Stage 3, two Knowledge Exchange workshops using a nominal group technique will be run. NHS managers, health professionals, commissioners, and patient and carer representatives will discuss DCE results and form a consensus on which ACP configuration best meets users' needs and is NHS feasible.

Ethics and dissemination

Ethical approval: NRES Committee (19/WM/0012) and King's College London ethics Committee (LRS-18/19-10353). Primary output will be identification of optimal ACP configuration which should be prioritised for implementation and evaluation. A pro-active dissemination strategy will make those considering developing or supporting an epilepsy ACP aware of the project and opportunities to take part in it. It will also ensure they are informed of its findings .

Project registration: researchregistry4723.

Strengths and limitations of this study (5 bullet points; one sentence each, relating to the methods)

- Project seeks to identify the optimal configuration of an 'Alternative Care Pathway' for epilepsy which could minimise clinically unnecessary and/or avoidable unplanned health service use and facilitate improved outcomes.
- The novel co-production approach employed – which will involve stakeholders at each project stage and use Discrete Choice Experiments (DCE) – will allow service users to inform service design in a scientifically robust, non-tokenistic way and could provide a template for service design more generally.
- Whilst most of the work packages will involve stakeholders from across England, patient and carers for the DCE are being recruited from one region. This might limit generalisability.
- The ACP configuration identified will be developed for use in the NHS and so may not be immediately applicable to other health systems. It will also require evaluation via a subsequent project.
- The project's success will depend, in part, on service users from what is a particularly vulnerable population agreeing to participate and representative samples being generated.

For peer review only - http://bmjopen.bmj.com/site/about/guidelines.xhtml

Key words: Epilepsy; Accident & Emergency Medicine; Organisation of health services; Heath economics; Qualitative Research.

List of Key Abbreviations

ACP(s)	Alternative Care Pathway(s)
DCE(s)	Discrete Choice Experiment(s)
ED	Emergency Department
GP	General Practitioner
NASH	National Audit of Seizure Management in Hospitals
NHS	National Health Service
NICE	The National Institute for Health and Care Excellence
NIHR	National Institute for Health Research
PPI	Patient and Public Involvement
PWE	People with Epilepsy
SMILE	Self-management Education for Adults with Epilepsy (Trial)
SSC	Study Steering Committee
UK	United Kingdom

5,269 words.

For peer review only

For peer review only - http://bmjopen.bmj.com/site/about/guidelines.xhtml

INTRODUCTION

Context and drive for health service innovations

The National Health Service (NHS), like other health systems, is being asked to make cost savings, whilst improving care experience, outcomes, and reducing health inequalities.[1, 2] Emergency department (ED) visits and admissions for the ambulatory care sensitive condition epilepsy provide an opportunity where service innovations could help achieve such aims. We describe a mixed-methods project to maximise the likelihood of such innovations being beneficial to service users and society.

Epilepsy and unplanned service use

With a prevalence of ~1%,[3] epilepsy is the UK's second most common serious neurological disorder. Annually, ≤20% of people with epilepsy (PWE) visit hospital emergency departments (ED).[4-6] They report more anxiety, seizures and perceived epilepsy stigma than those in the wider epilepsy population and live in more deprived areas; [7-12] ~20% have an intellectual disability.[13, 14]

The annual cost to the NHS of these visits in England alone is ~£70-90M.[15-17] Costs are high because half of the visits result in hospital admission.[6, 18-21] An unusually high re-attendance rate also inflates cost; [22, 23] ≤60% of PWE re-attend ED within 12 months.[7]

Most PWE visiting ED do not attend with a life threatening or emergency presentation, (e.g., status epilepticus, first seizure). Rather, projects like our National Audits of Seizure Management in Hospitals (NASH) [13, 14] show that most have known, rather than new epilepsy, and present with states not requiring ED's full facilities. Leading presentations include someone who has experienced: i) an uncomplicated seizure in line with their usual presentation; ii) a seizure in public and cannot be 'left at scene'); and iii) a self-terminating seizure, different to their usual presentation.[20]

Unmet needs of PWE visiting ED

Whilst the acute episodes leading PWE to visit ED do not typically require emergency care, the visits can be expressions of the person having received suboptimal ambulatory care and unmet needs. NASH [24], for instance, found that most (~65%) PWE visiting ED are not known to specialist epilepsy services and many were via their usual care provider, seemingly receiving outdated care. For instance, despite focal epilepsy being the most common epilepsy type, most likely to be refractory and often not best treated with the medication sodium valproate, NASH found it to be the most prescribed medication amongst ED attendees.

Coping with life in the context of epilepsy also requires an individual to accept their diagnosis and learn and adopt a range of self-management behaviours to prevent seizures and manage consequences. PWE visiting ED though and their significant others appear to

For peer review only - http://bmjopen.bmj.com/site/about/guidelines.xhtml

have less knowledge about epilepsy and its management, including seizure first aid.[7, 25-27] They might therefore benefit from enhanced self-management support, like that provided by epilepsy nurses. The reason that knowledge might be low in ED attendees is because there remains no routine course that all PWE can go on to learn about epilepsy (as there are for some conditions). People who have lower education levels appear to fare worst from this situation with them having been found to have the least epilepsy knowledge.[28, 29]

That some PWE in the UK are receiving suboptimal care is well known, with there being longstanding challenges in ensuring that the PWE most in need of specialist care receive it. Whilst trial evidence indicates ~70% of PWE can become seizure free, only ~50% of PWE in the UK currently are,[30] with PWE in socially deprived areas faring the worst.[31] Factors contributing to the challenge are that the UK has a comparatively small specialist workforce [32] and that there is no national, incentivised system to identify those within the epilepsy population that might need stepped-up care. To further complicate matters, GPs, who care for most PWE, have expressed low confidence in managing the condition.

ED visits by PWE can be considered opportunities to intervene. Unfortunately, under current arrangements, going to ED does not typically lead to PWE receiving ambulatory care improvements. Bodies, such as NICE[2], recommend that when seizures are not controlled, a patient should be referred to specialist services (within 4 weeks) since this may improve outcomes, including rendering some seizure free.[33, 34] Most (80%) PWE visiting ED are though not seen by a specialist during their attendance, their usual care providers may not be informed of the attendance [13] and most (60%) are not referred to a specialist for follow-up.

PWE living in the most deprived areas, as well as the elderly, are amongst the least likely to be referred on from ED.[35] The low referral rate may be due to some clinicians holding an incorrect nihilistic view that intractability is inevitable if seizure control is not obtained within a few years of therapy onset. Assumptions about the willingness of certain patient groups to attend clinics may also be being made.[36, 37]

An 'Alternative Care Pathway'

NHS policies [1, 2, 38] and publicity given to NASH's findings [15, 39] created momentum to reduce visits for seizures and enhance patient outcomes. NICE [40] found no evidence on how to do this. However, one idea gaining traction is for ambulance services to assume a greater role.[41] Most (~90%) people visiting ED for seizures have been transported there by an emergency ambulance.[13, 14, 21]

Data from some regional ambulance services on conveyance rates for seizures has been published. It indicates ambulance staff are recommending conveyance of nearly every person they attend for a suspected seizure to ED,[42-44] despite most not demonstrating a clinical need (e.g., seizures have self-terminated before ambulance arrival in ~90% of

For peer review only - http://bmjopen.bmj.com/site/about/guidelines.xhtml

cases),[43] One reason for this is paramedics lack access to alternatives.[45-47] There is a vision therefore of what could help: ambulance service access to some form of 'Alternative Care Pathway' (ACP) whereby those seeking help for an epileptic seizures judged not to require ED are cared for within less costly, alternative environments.

The exact nature of the ACP is not clear. Different regions and services are considering different configurations and doing so in an uncoordinated way. ACPs being considered include paramedics transporting patients' home or to an urgent treatment centre rather than ED. Others involve paramedics leaving patients at scene with the offer of a telephone call from a nurse or general practitioner with a specialist interest in epilepsy within 24, 48 or 72 hours. A possible benefit of this latter configuration is health inequalities could be reduced by introducing a mechanism by which all PWE 'in need' are brought to specialists' attention.

ACPs are not new [48] and paramedics have not been obliged to transport patients to ED since ~1997. The ambitions of ACPs have though increased and there is significant support within the UK policy realm to expand the number of conditions covered by them as a means of managing demands on hospital services (e.g. [49-51]).

ACPs within the ambulance have largely come about due to the 'ISLAGIATT' ('it seemed like a good idea at the time') principle,[52] rather than with reference to a behaviour change theory. Evidence on the utility of ACPs is though generally positive.[48, 53-55] In their review of potential revisions to the urgent and emergency care system, the Nuffield Trust identified greater ambulance/paramedic triage in the community as having the most the positive evidence of effectiveness.[41] Paramedics appear willing and safely able to use ACPs when trained and for some ACPs there is evidence of cost-savings and greater patient satisfaction.[53, 54]

In the case of epilepsy, qualitative research [45-47] provides the beginnings of a theoretical basis for the use of an ACP in epilepsy with the mechanisms by which it could make a difference being that it may: increase awareness and likelihood that paramedics will consider non-conveyance and referral pathways as an option in appropriate cases; increase paramedics' clinical knowledge of how to make appropriate non-conveyance decisions; increase paramedics' knowledge of alternative care providers that are acceptable to service users; and increase paramedics' confidence about making a non-conveyance decision and reducing anxiety about risk.

Developing an ACP for epilepsy

An ACP for epilepsy holds potential. As a team of health care professionals, researchers and service user representatives with expertise in epilepsy and urgent care, our ultimate goal is therefore to evaluate the most promising ACP and use the evidence to transform service organization nationally. However, we cannot currently do this because the way in which ACPs

For peer review only - http://bmjopen.bmj.com/site/about/guidelines.xhtml

are being developed means it is not known which of the many ACPs has the potential to be most effective and could be justified for selection.

Specifically, ACPs are being developed largely ‘top-down’, with the patient voice being absent. The nature and content of ACPs may not therefore align with what patients/carers would consider to meet their needs. Evidence suggests decisions to access healthcare services can be informed by how a patient/carer perceives their situation [56-58] and there can be a mismatch between patients/carers and health professionals’ views, including of what constitutes an ‘emergency’.[59-62] The upshot is the acceptability – a fundamental criterion an intervention needs to satisfy to be positioned to achieve its intended outcome [52] – of the different ACPs to patients and carers is unknown.

To date, only one epilepsy ACP has been reported on.[63] Despite the evaluation revealing positive outcomes, less than 10% of eligible PWE attended to by paramedics were put onto the ACP. The reasons for this low uptake rate were not explored. A possible explanation is that the ACP was not acceptable to PWE.

It has been assumed the target population *does not* want to be conveyed to ED and will readily accept an ACP. It may not be that straightforward. Some PWE certainly express dissatisfaction with being taken to ED.[64] Others though express a need for immediate access to urgent care; with some PWE and their family and friends (to whom care decisions are often delegated to when the patient is unconscious or lacks capacity) being fearful of seizures, including the possibility of brain damage.[26, 27]

CURRENT PROJECT

This 28-month project seeks identify the ACP that should be prioritized for testing/ implementation. It seeks to shape the change that results from the identified momentum so the likelihood of patients and the NHS benefiting are maximised and finite health resources used in an informed, rational way. It will bring patients/carers from the target population, health care professionals and commissioners together to identify which ACP configuration best encapsulates the features important to patients and carers *and* is feasible within the NHS context. The focus will be on the care of adults with epilepsy, rather than children/young people, since discussion to date relating to the use of ACPs and ED care has largely focused on the former (e.g., [24, 63]). As per NICE guidelines for epilepsy,[40] adults are defined as those aged 18 years and older.

The project has 3 linked stages and will use survey techniques, qualitative methods, consensus meetings and Discrete Choice Experiments (DCEs) to achieve its aims (Figure 1).[65, 66] DCEs are an attribute-based survey methodology, underpinned by the theory that any ‘good’ (including a health service) can be described by its constituent characteristics (attributes), and that the extent to which an individual prefers a ‘good’ depends on the levels

For peer review only - http://bmjopen.bmj.com/site/about/guidelines.xhtml

these attributes take.[67] We considered DCEs to provide an efficient, scientifically defensible, and non-tokenistic way of bringing the patient voice into ACP design since they allow a person's stated preferences and priorities to be elicited by presenting them with a hypothetical scenario (e.g., having a seizure at home) and asking them to choose which of two (or more) care options described by a series of attributes they prefer. The process is then repeated with alternative care choices being presented. By varying attribute levels and observing how participants change responses, the importance of attributes and the extent to which they drive preference can be inferred.

Aims

a) Systematically identify ACPs being considered by the NHS for epilepsy and describe extent to which service users have been involved in their design (Stage 1a).
b) Understand patient and carers' decision-making processes for seeking or not seeking ED care, and their concerns and expectations regarding ACPs (Stage 1b).
c) Identify the attributes of post-seizure emergency care that PWE and their carers consider important and identify if this differs by the context in which the seizure occurs (Stage 1b).
d) Determine patient and carer preferences for post-seizure emergency care using DCEs and identify any subgroup differences (Stage 2b).
e) Estimate uptake of different ACPs configurations by patients and subgroup differences (Stage 2b).
f) Hold expert workshops to develop an optimal ACP following seizures, to be prioritised for implementation and evaluation via a subsequent project (Stage 3).

METHODS AND ANALYSIS

This project will use qualitative and quantitative methods (Figure 1). According to Greene et al.'s [68] conceptual framework, the purpose of using a mixed methods approach is 'development' (seeking to use the results from one method to help develop or inform the other method, where development is broadly construed to include sampling and implementation, as well as measurement decisions; Stages 1-2b) and 'expansion' (seeking to extend the breadth and range of inquiry by using different methods for different inquiry components; Stages 2b and 3).

Data collection and analysis for Stages 1a and 1b will be completed concurrently and independent from one another. From Stage 1c onwards, the design will be sequential and dependent. During Stage 2a, the findings from the earlier stages will be integrated to develop the DCE. Following completion of analysis for Stage 3, findings from all the stages will be

For peer review only

For peer review only - http://bmjopen.bmj.com/site/about/guidelines.xhtml

integrated in a narrative form when interpreting and reporting on the project's findings. Particular attention will be given to how the findings from Stage 3 converge and diverge from the qualitative and quantitative findings from the earlier stages.

Stage 1

Stage 1a: Pathway survey

Purpose: Systematically identify ACPs being considered. Will allow ACPs presented within DCEs to include main ones being considered.

Design: Cross-sectional survey of NHS service providers asking respondents whether their service has considered (or implemented) any changes to the management of those with epilepsy/suspected seizures to minimise clinically unnecessary/avoidable ED visits. Respondents will report how change is anticipated to minimise visits and whether service users informed change.

The survey will take ~10 minutes, be piloted and completed online. Survey respondents will be able to indicate if they want to be informed of subsequent project parts.

Recruitment: Invitations to participate, followed by two reminders, will be sent to clinical leads/ directors of England's regional ambulance trusts (n=11), regional neuroscience (~n=25) [69] and neurology centres (~n=16),[70, 71] and a random sample of 25% of its 'Type 1' EDs [72] (n=35), stratified by geographical area and size (attendances).

Analysis: Characteristics of responding and non-responding organisations will be compared. To determine the types of ACPs being considered and proportion of organisations considering each, a subset of surveys will be selected. A qualitative researcher, supported by the wider team, will read these to identify recurrent ACP types and collate them into a thematic coding framework. The framework will be applied to the full data set and modified to ensure all ACPs types are captured.

Stage 1b: Qualitative interviews

Purpose: Understand patient and carer decision-making, the important attributes of post-seizure care, and concerns and expectations regarding ACPs. Will permit attribute development to adhere to good practice.[73, 74]

Design: Face-to-face semi-structured interviews will be conducted with PWE, and where possible an informal carer, and shaped by a piloted topic guide (Supplementary File 1).

For peer review only - http://bmjopen.bmj.com/site/about/guidelines.xhtml

Participants will be asked for contextual information about their epilepsy and ED visits. The researcher will explore patients and carers confidence in managing seizures and the decision to call the ambulance services. The influences on ambulance use and ED use when seizures occur in public places will also be examined. Evaluative information will then be sought regarding positive and negative perceptions of the ambulance service and casualty as well as any ACPs experienced. Potential attributes will be identified by describing two known ACPs (i.e., urgent care centres and being taken home) and asking participants for their expectations and concerns.

To help reduce the number of care attributes and improve face validity, participants will rank what they consider to be the five most important care attributes. Interviewer will help recall those mentioned and write them onto show cards.

To promote consistency, interviews will be conducted by one experienced qualitative researcher [AMK]. AMK (PhD), is a university-based qualitative researcher with an interest in health services research but no specialist knowledge of, or involvement in, the ambulance or emergency medical services. Before agreeing to take part, participants will be given a participant information sheet detailing the research. Written informed consent will be obtained [by AMK] before the interviews. No non-participants will be present during the interviews. Interviews will take place at a time and place convenient to the participant. It is anticipated they will last 60-90 minutes.

To promote transparency, meticulous records of the interviews will be kept, interviews audio-taped, transcribed verbatim [75] and reported according to COREQ.[76]

Recruitment: Aim is to capture a comprehensive range of perspectives, and sample size will be determined by data saturation. We anticipate [73, 77] recruiting a purposive sample of ~30 PWE, with or without an informal carer. Table 1 lists the inclusion and exclusion criteria.

Eligible PWE will be identified by two means. Firstly, persons with uncontrolled epilepsy who participated in a recent NIHR funded 'Self-Management Education for adults with Epilepsy' (SMILE) trial in the South-East of England and expressed interest in future projects will sent notification of the study.[78] Secondly, adverts will be circulated to persons affiliated with epilepsy user groups, including Epilepsy Action.. Persons interested in taking part will be asked to contact the research team. To identify carer participants, patients will be asked to choose a significant other/informal carer to take part. Participants will receive a £20 shopping voucher.

Analysis: Analysis will proceed alongside data collection and be based on a Framework approach.[79] This approach ensures each case is fully accounted for and is regarded as particularly helpful for addressing policy questions. The primary aim of analysis will be to

For peer review only - http://bmjopen.bmj.com/site/about/guidelines.xhtml

generate an exhaustive list and description of the salient attributes of care (and levels) identified by participants.

The analytic approach will involve the identification of initial themes that are then grouped into main themes and subthemes. Transcripts will be read and re-read, and coded. At least two members of the research team (AMK, MM, LR) will participate in all data analysis to reduce bias in the identification and interpretation of themes.

A thematic 'chart' will be created to summarise information for each theme to allow cross-case and within-case analysis through a process of constant comparison, with particular attention being paid to deviant cases. Analysis will pay attention to the similarity and differences in salient attributes according to where the seizure occurred (home, public), who was there, who made the 999 call, whether the patient had seen a specialist in the prior 12 months, and whether they self-reported an intellectual impairment. Participant quotations (with minor editing where necessary to preserve anonymity) will presented to illustrate themes and help verify interpretation. QSR International's NVivo 11 qualitative data analysis software (80) will be used as a management tool throughout the process.

The attributes nominated during the ranking exercise will be categorised according to the thematic framework, then analysed quantitatively [by EH] using standardised rank scores.

Stage 1c: Knowledge exchange event

Purpose: It is important the DCE elicits views on ACPs that could plausibly be delivered within the NHS. This event will therefore, early in the project, determine feasibility of the attributes and associated levels that have been identified by service users in Stage 1b.

Design: One-day event at which health care professional representatives will be shown draft attributes, levels, and choice sets and asked to deliberate each choice. For instance, should Stage 1b indicate service users consistently say they prefer to be transported home by the ambulance service regardless of distance, then representatives would be asked to what constitutes a feasible distance. Our team has previously used this approach.(81)

Recruitment: Around n=15 ambulance staff and neurology service representatives. Persons will be recruited from organisations participating in Stage 1a with the help of a sampling framework to ensure representatives come from geographically diverse areas. Representatives will be reimbursed for travel. Those not able to attend will be able to provide feedback remotely.

Analysis: With attendees' consent, the session will be audio-recorded and field notes taken. Feedback will be used to optimise the attribute levels within the DCEs.

For peer review only - http://bmjopen.bmj.com/site/about/guidelines.xhtml

Stage 2

Stage 2a: Developing the DCE

Purpose: Generate DCE questionnaire to elicit patient and carer preferences for post-seizure care and which meets best practice guidance.[73, 82]

Design: A multidisciplinary panel, chaired by DH, will compose a draft DCE questionnaire using Stage 1 evidence and refine it on the basis of a pilot. The panel will include expertise in DCEs [EH], emergency medicine [SG], neurology [AGM], paramedical science [MJ], general practice [JD], and psychology [AN], as well as having service user representation.

The panel will form a consensus on which restricted number of ACPs best represent the range being considered and are to be valued using the DCE. Using the list of candidate attributes generated from Stage 1b the panel will select 4-6 attributes by which to describe the packages.

In selecting attributes, the panel will favour those prioritised by service users, which are modifiable, capable of being traded and best describe the ACPs.[73] Attributes deemed to be too close to the latent construct of the utility derived from the ACP (e.g., 'satisfaction') will not be selected.

DCEs will be generated for the three most common presentations to ED by PWE. For each, participants will be presented with a seizure scenario vignette and via standardised instructions complete a series of forced, pairwise comparisons to indicate which care packages they would prefer. Vignettes will be developed by the panel based on clinical and lived experience. Supplementary File 2 provides an illustration of what a DCE *might* look like.

Plausible attribute levels will be specified on the basis of findings from Stage 1, expert opinion and relevant literature. A strength of DCEs is the possibility of varying attribute levels to also model preferences within future contexts. Using its expertise and knowledge, the panel will seek to assign levels to attributes to account for major anticipated changes – such as longer ED waiting times.[83]

A fractional factorial design will identify a purposeful subset of options for each DCE, to allow an estimate of the main effect of each attribute independently and selected two-factor interactions, while minimizing the number of paired comparisons participants are asked to make. The profiles will be obtained from a published design catalogue.[84] Binary choices will then be created using the fold-over method, which replaces each attribute level systematically.[85]

Once drafted, the DCE will undergo iterative individual pilot testing with ~15 PWEs and carers from the project's Patient and Public Involvement (PPI) group to check clarity and duration. Pilotees will 'think aloud' [86] when making choices, and asked to consider their

For peer review only - http://bmjopen.bmj.com/site/about/guidelines.xhtml

preferred presentation of attributes (e.g. text, pictograms). Sessions will be audio recorded, notes made and refinements made.

Stage 2b: Formal DCE

Purpose: Determine patient and carer preferences for post-seizure emergency care, estimate uptake of different ACPs configurations, and subgroup differences.

Design: A representative sample of PWE and informal carers who have visited ED in the last 12 months, as well as those at risk, will be sought to complete the finalised DCE questionnaire. PWE who have uncontrolled epilepsy, but who have not visited ED in the last 12 months will be asked to visit an online survey page and complete the DCE questionnaire using their own internet enabled devices. Due to potentially greater social disadvantage, patients who *have* visited ED in the prior 12 months will have the additional option of completing the DCE during a face-to-face appointment with a researcher who will provide an internet enabled device.

To limit burden, participants will complete DCEs relating to just two seizure scenarios (allocated at random). As well as the DCEs, respondents will report on their demographics, epilepsy, knowledge and fear of seizures, and service use. Each participant will receive a £20 shopping voucher.

Recruitment: General Practices in North-West England will identify potential patient participants by searching their epilepsy registers.[(87)] Practices will be recruited with the help of the NIHR Clinical Research Network North West Coast. Practices will send ostensibly eligible patients invitation packs via 'Docmail' and a repeat invite 2 weeks later to those not responding. Patients not wishing to participate will be encouraged to notify the team and detail their reasons. Eligibility criteria are in Table 1.

A definitive sample size calculation depends on the finalized design of the DCE.(88) To permit decisions regarding recruitment to be made in the meantime we anticipated the design and calculated the required sample size by using Orme's[(89)] formula. Full details are provided in Table 2. This indicated 348 PWE (or associated informal carers) would be required, comprising data 174 patients (or associated carers) who have visited ED in the prior 12 months and 174 patients (or associated carers) who have not.

Based on responses to prior health DCEs [(90)] and studies with PWE,[(16, 25)] we anticipate 30-60% of those invited will agree to participate. An average English GP practice [155] will have 24 PWE aged ≥18 on their register who have experienced ≥1 seizure in the prior 12 months,[9] with 10 having visited an ED during that period.[12-14] Governed by the need to recruit a sub-group of 174 PWE who have visited ED in the prior 12 months, 29-58 general practices will be required.

For peer review only - http://bmjopen.bmj.com/site/about/guidelines.xhtml

Analysis: Data for the different seizure vignettes will be analysed separately using random effects logit regression that allows for multiple observations from the same respondent. This will determine the importance of the different care attributes for preference and direction of effect (as indicated by the significance of the coefficients and their size). Comparison between vignettes will depend on attribute and level specification of each experiment. Analyses will be completed with and without respondents failing a 'rationality' test.

Marginal rates of substitution (the rate at which respondents are willing to give up a unit change in one attribute in exchange for a unit change in another while maintaining the same level of utility) will be calculated using each quantitative attribute as the value attribute with bootstrapped confidence intervals (1000 reps).

Heterogeneity in preference will be assessed, using subgroup analysis and log likelihood ratio testing, and the model specified accordingly.[(91)] A-priori subgroups of interest are: (1) emergency care for epilepsy in the past 12-months (paramedic and/or ED visit), (2) social deprivation (with participants grouped according to how socially deprived the area is within which they live); (3) whether or not they have seen an epilepsy specialist within the prior 12 months (which they should have according to NICE guidelines [89] given they will all have uncontrolled epilepsy); and (4) whether they or the person they know in the case of carers, self-reports an intellectual disability.

'Utility scores' for different ACP configurations and significantly different populations will be calculated and ranked accordingly. Their uptake under different seizure scenarios will be estimated.

All analyses will be conducted in STATA 11 (StataCorp, College Station, TX, USA).

Stage 3: *Knowledge Exchange workshops*

Purpose: Disseminate Stage 1 and 2 findings to those positioned to develop, fund, support and run ACPs and utilise their expertise to identify which ACP/s favoured by patients and carers is most NHS feasible and should be prioritised for implementation/ evaluation.

Design: One Knowledge Exchange event in South England, one in the North. They will start with presentation of project's findings, including ACP configurations ranked according to service user preference. A Nominal Group Technique, described by the James Lind Alliance, [(92)] will then be used to hear delegates' views of the ACPs and rank them according to the extent to which they meet users' preferences and are NHS feasible. Facilitators will encourage delegates to consider barriers to change, supply constraints, acceptability of the service to staff, possible cost, and potential to redress health care inequalities.

For peer review only - http://bmjopen.bmj.com/site/about/guidelines.xhtml

Voting will be completed using electronic devices. With attendees' consent, sessions will be audio-recorded, and a qualitative researcher will observe and record field notes.

Recruitment: Aim to have 40-50 health service managers, commissioners, healthcare professionals, and service user representatives at each event. Persons will be recruited mainly from the institutions participating in Stage 1a. Additional attendees will come from commissioning groups and the epilepsy patient groups. The project's PPI group will also be invited. A sampling matrix will be formed to help ensure broadly equal representation of persons from health professional, commissioning and managerial roles.

Analysis: The rank order of the ACPs that results from delegates voting will be described. Depending on findings from the previous stages, the ACPs might have been presented to delegates according to seizure scenario. If so, rank orders for the ACPs for the different scenarios will be determined and compared. As a secondary objective, rank orders for the different representative subgroups will be determined. Transcripts from the events will be thematically analysed to capture delegates' views and justifications for their preferences.

DISCUSSION

Patient and public involvement

This research was driven by a need to consider the views of PWE when designing an ACP for epilepsy. Epilepsy Action, the largest user organisation in the UK, contributed to the design of the research, and facilitated a PPI event with 23 service users to discuss project design and conduct. A PPI group will be established, including PWE and carers. They will be supported by Epilepsy Action who have an active PPI scheme and reimbursed for travel and time according to INVOLVE.[93] The group will contribute as research peers by: providing feedback on research materials; interpretation of findings; piloting the DCE; and being delegates in workshops. The group will be encouraged to help with dissemination, including presenting the findings at the Stage 3 events and teaching.

Ethics and dissemination

Monitoring by an independent Study Steering Committee (SSC) will help to ensure the rights, safety and well-being of participants are the most important considerations. Only persons providing informed consent will participate. The SSC will be composed according to our funder guidelines.[94] Compliance with the principles of Good Clinical Practice and scientific integrity will be managed by the study management team through regular and ad hoc meetings.

For peer review only - http://bmjopen.bmj.com/site/about/guidelines.xhtml

The project will identify what, if any, ACP configuration/s is most acceptable to service users and has the backing of those expected to deliver and support it. To ensure maximum impact those considering an ACP need to be aware of the project, have confidence in its outputs and use its findings. To help ensure this: 1) Representatives from all of the relevant service providers in the UK will be informed of the project (including notifying the leads of England's Sustainability and Transformation Partnerships and Urgent and Emergency Care Vanguards); 2) professional bodies will announce its funding (Acknowledgements); 3) the research team includes appropriate expertise and opinion leaders; and 4) representatives from relevant NHS services will be invited to take part in Stage 1a and 3a.

The project's findings will be disseminated via international conferences and at least four journal manuscripts. The manuscripts will be published open access and non-technical reports will be circulated via NHS network newsletters and Epilepsy Action. Participants will be provided with a copy of the results.

Having identified the ACP, funding will stop but the team would, via a subsequent project seek to implement and evaluate the chosen ACP. The nature of that evaluation will be informed by the state of the NHS closer to the time. The Medical Research Council [95] highlight intervention evaluation takes place in a wide range of settings and many factors can constrain choice of evaluation methods. One trade-off that may need consideration is time until results become available/ demand versus methodological rigour.

For peer review only - http://bmjopen.bmj.com/site/about/guidelines.xhtml

Acknowledgements: The authors would like to thank Epilepsy Action (Angie Pullen, Amanda Stoneman) for the role that they will have in this project. They would like to express appreciation for the contributions from people with epilepsy and their family members and friends who will participate in this study and thank members of the Study Steering Committee (Tom Quinn [chair], Verity Watson, Trevor Baldwin, Nigel Rees, Khalid Hamandi, Daniel Horner, Juliet Bransgrove, and Jayne and Sam Burton). We also acknowledge the International League Against Epilepsy (British Branch), Royal College of Emergency Medicine and the Epilepsy Nurses Association for informing members of the project. The study sponsor is the University of Liverpool (reference: UoL4258; sponsor@liv.ac.uk).

Author Contributions: AJN conceived of the study and designed it together with EH, DH, LR, MM, MJ, JD, SG and AGM. EH planned the statistical analysis for the DCE. AJN and AM wrote the manuscript, with revisions being made by EH, AM, DH, LR, MM, MJ, JD, SG and AGM. All authors read and approved the final manuscript.

Funding statement: This project is funded by the National Institute for Health Research's Health Services and Delivery Research Programme (HS&DR Programme) (project number 17/05/62). The views and opinions expressed herein are those of the authors and do not necessarily reflect those of the University of Liverpool, the HS&DR programme, the NIHR, the NHS, or the Department of Health and Social Care.

Competing interests: None declared.

Patient consent for publication: Not required.

Data statement: This is a protocol. There is no data associated with it.

Ethics approval: This study has been received Health Research Authority approval and favourable ethical opinions from NRES Committee West Midlands - Solihull (19/WM/0012) and King's College London's Psychiatry, Nursing and Midwifery Ethics Committee (LRS-18/19-10353).

For peer review only

Table 1 Participant inclusion and exclusion criteria

Study part	Inclusion criteria	Exclusion criteria
**Stage 1b – Qualitative interviews**		
	▪ Established diagnosis of epilepsy, or an informal carer for someone with epilepsy<br>▪ Age ≥18 years (no upper age limit)<br>▪ Have visited ED in the past 12 months for epilepsy (as reported by the patient)*<br>▪ Able to provide informed consent and communicate in English	▪ Severe current psychiatric disorders (e.g. acute psychosis)<br>▪ Life-threatening medical illness
**Stage 2 – Discrete Choice Experiments**		
**Group 1**		
**Patients**	▪ Clinically confirmed diagnosis of epilepsy (for any duration)<br>▪ Any epilepsy syndrome and any types of focal or generalised seizures<br>▪ Currently being prescribed antiepileptic medication<br>▪ Age ≥18 years (no upper age limit)<br>▪ Have visited ED in the past 12 months for epilepsy<br>▪ Able to provide informed consent and independently complete a questionnaire in English<br>▪ Lives in the North West of England	▪ Severe current psychiatric disorders (e.g. acute psychosis)<br>▪ Life-threatening medical illness<br>▪ Resides within a care or nursing home or of no fixed abode
**Carers**	▪ A significant other to a person with epilepsy (e.g. family member,	▪ Severe current psychiatric disorders (e.g. acute psychosis)

For peer review only

For peer review only - http://bmjopen.bmj.com/site/about/guidelines.xhtml

	friend) who the patient identifies as providing informal support or self-identifies themselves if the patient has a substantial intellectual disability<br>▪ The person with epilepsy they care for has visited ED in the past 12 months<br>▪ Age ≥16 years (no upper age limit)<br>▪ Able to provide informed consent and independently complete a questionnaire in English<br>▪ Lives in the North West of England	▪ Life-threatening medical illness
**Group 2**		
**Patients**	▪ Clinically confirmed diagnosis of epilepsy (for any duration)<br>▪ Any epilepsy syndrome and any types of focal or generalised seizures<br>▪ Currently being prescribed antiepileptic medication<br>▪ Age ≥18 years (no upper age limit)<br>▪ Has *not* visited ED in the past 12 months, but has had a seizure with loss of awareness in this period<br>▪ Able to provide informed consent and independently complete a questionnaire in English<br>▪ Lives in the North West of England	▪ Severe current psychiatric disorders (e.g. acute psychosis)<br>▪ Life-threatening medical illness<br>▪ Resides within a care or nursing home or has no fixed abode
**Carers**	▪ A significant other to a person with epilepsy (e.g. family member, friend) who the patient identifies as providing informal support or self-identifies themselves if the patient	▪ Severe current psychiatric disorders (e.g. acute psychosis<br>▪ Life-threatening medical illness

For peer review only

has a substantial intellectual disability

- The person with epilepsy they care for has *not* visited ED in the past 12 months, but had a seizure with loss of awareness in this period
- Age ≥16 years (no upper age limit)
- Able to provide informed consent and independently complete a questionnaire in English
- Lives in the North West of England

Notes: ED, emergency department; *Should recruitment prove slower than anticipated, the recruitment criteria will be relaxed to allow people who have had ED or ambulance contact within the last 2 years.

For peer review only

For peer review only - http://bmjopen.bmj.com/site/about/guidelines.xhtml

Table 2 Sample size calculation for Discrete Choice Experiments

**Formula used and factors considered in estimating likely sample size required**
• Orme's formulae: (*n***t***a*)/ *c* must be ≥500, where *n* is number of respondents, *t* number of tasks, *a* number of alternatives per task, and *c* number of analysis cells (for main effects, the largest number of levels for any one attribute). • Anticipated Discrete Choice Experiments (DCEs) to involve participants being asked to make (*t*) 9 separate choices between (*a*) 2 care packages and (*c*) 3 would be maximum attribute levels. Thus, Orme's formula indicated complete data from n=84 needed. • Intend to complete DCEs for three different seizure scenarios, but participants would only complete two, therefore 126 participants required. • To allow for variation in the finalized number of choice tasks and levels we increased this to 150 and further inflated it to 174 to accommodate for ~15% of missing data experienced by prior epilepsy DCEs. • As an objective was to describe and compare care preferences of two subgroups (patients (and associated carers) who have visited an ED in the prior 12 months vs. patients (and associated carers) who have had a seizure in the prior 12 months, but have not visited an ED), 174 patients (and associated carers) who have visited ED in the prior 12 months and 174 patients (and associated carers) who have not, will need to be recruited. • Total sample size, thus n=348.

For peer review only - http://bmjopen.bmj.com/site/about/guidelines.xhtml

Figure Legend

Figure 1: Research project process diagram.

Notes to Figure 1: HRA, Health Research Authority; EDs, Emergency Departments; PWE, People with Epilepsy; DCEs, Discrete Choice Experiments; ACP/s, Alternative Care pathway/s

For peer review only

For peer review only - http://bmjopen.bmj.com/site/about/guidelines.xhtml

REFERENCES

1. NHS England. Five Year Forward View 2014 [Available from: https://www.england.nhs.uk/wp-content/uploads/2014/10/5yfv-web.pdf.
2. NHS England. Next Step on the NHS Five Year Forward View 2017 [Available from: england.nhs.uk/wp-content/uploads/2017/03/NEXT-STEPS-ON-THE-NHS-FIVE-YEARFORWARD-VIEW.pdf.
3. Ridsdale L, Charlton J, Ashworth M, Richardson MP, Gulliford MC. Epilepsy mortality and risk factors for death in epilepsy: a population-based study. Br J Gen Pract. 2011;61(586):e271-e8.
4. Hart YM, Shorvon SD. The nature of epilepsy in the general population. II. Medical care. Epilepsy research. 1995;21(1):51-8.
5. Jacoby A, Buck D, Baker G, McNamee P, Graham-Jones S, Chadwick D. Uptake and costs of care for epilepsy: findings from a UK regional study. Epilepsia. 1998;39(7):776-86.
6. Kitson A, Shorvon S. Services for patients with epilepsy: Report of a CSAG committee. Department of Health; 2000.
7. Noble AJ, Goldstein LH, Seed P, Glucksman E, Ridsdale L. Characteristics of people with epilepsy who attend emergency departments: Prospective study of metropolitan hospital attendees. Epilepsia. 2012;53(10):1820–8.
8. Bautista RED, Glen ET, Wludyka PS, Shetty NK. Factors associated with utilization of healthcare resources among epilepsy patients. Epilepsy research. 2008;79(2-3):120-9.
9. Allard J, Shankar R, Henley W, Brown A, McLean B, Jadav M, et al. Frequency and factors associated with emergency department attendance for people with epilepsy in a rural UK population. Epilepsy & Behavior. 2017;68:192-5.
10. Public Health England. The NHS Atlas of Variation in Healthcare: Reducing unwarranted variation to increase value and improve quality. 2016 [Available from: fingertips.phe.org.uk/documents/Atlas_2015%20Compendium.pdf.
11. QIPP/Right Care. The NHS atlas of variation in healthcare 2010 [Available from: fingertips.phe.org.uk/documents/Atlas_2010%20Compendium.pdf.
12. Right Care. The NHS Atlas of Variation in Healthcare: Reducing unwarranted variation to increase value and improve quality. 2011.
13. Pearson M, Marson A, Dixon P, Scott K. National Audit of Seizure Management in Hospitals: St. Elsewhere's Hospital Clinical Report 2014 [Available from: http://www.nashstudy.org.uk/Newsletters/St%20Elsewhere's%20Clinical%20Report%20NASH%202.pdf.
14. Pearson M, Marson A, Dixon P, Billington K. National Audit of Seizure Management in Hospitals St Elsewhere's Clinical Report 2012 [Available from: http://nashstudy.org.uk/Newsletters/St%20Elsewhere%27s%20NASH%20Report.pdf.
15. House of Commons Committee of Public Accounts. Services to people with neurological conditions: progress review London House of Commons; 2015 [Available from: https://publications.parliament.uk/pa/cm201516/cmselect/cmpubacc/502/502.pdf.
16. Ridsdale L, McCrone P, Morgan M, Goldstein L, Seed P, Noble A. Can an epilepsy nurse specialist-led self-management intervention reduce attendance at emergency departments and promote well-being for people with severe epilepsy? A non-randomised trial with a nested qualitative phase. Health Services and Delivery Research 2013;1(9).
17. Dickson JM, Jacques R, Reuber M, Hick J, Campbell MJ, Morley R, et al. Emergency hospital care for adults with suspected seizures in the NHS in England 2007–2013: a cross-sectional study. BMJ open. 2018;8(10):e023352.
18. Bardsley M, Blunt I, Davies S, Dixon J. Is secondary preventive care improving? Observational study of 10-year trends in emergency admissions for conditions amenable to ambulatory care. BMJ open. 2013;3(1):e002007.

For peer review only - http://bmjopen.bmj.com/site/about/guidelines.xhtml

19. Peter A Dixon, Jamie J Kirkham, Anthony G Marson, Pearson MG. National Audit of Seizure management in Hospitals (NASH): results of the national audit of adult epilepsy in the UK BMJ Open 2015;5(3):e007325.
20. Reuber M, Hattingh L, Goulding PJ. Epileptological emergencies in Accident and Emergency: a survey at St James's University Hospital, Leeds. Seizure. 2000;9(3):216-20.
21. Ryan J, Nash S, Lyndon J. Epilepsy in the accident and emergency department--developing a code of safe practice for adult patients. South East and South West Thames Accident and Emergency Specialty Sub-committees. . Emergency Medicine Journal 1998;15.
22. Whiston S, Coyle B, Chappel D. Health needs assessment for long term neurological conditions in North East England. Stockton on Tees: North East Public Health Observatory; 2009.
23. NICE. Services for the diagnosis and management of the epilepsies in adults, children and young people: commissioning guide 2013 [Available from: nice.org.uk/guidance/qs26/resources/services-for-thediagnosis-and-management-of-the-epilepsies-in-adults-children-and-young-people-commissioning-guide-317820637/chapter/3-Assessingservice-levels-for-the-diagnosis-and-management-of-the-epilepsies#ftn.footnote_19.
24. Dixon PA, Kirkham JJ, Marson AG, Pearson MG. National Audit of Seizure management in Hospitals (NASH): results of the national audit of adult epilepsy in the UK. BMJ Open. 2015;5:e007325.
25. Noble AJ, Marson AG, Tudur-Smith C, Morgan M, Hughes DA, Goodacre S, et al. 'Seizure First Aid Training' for people with epilepsy who attend emergency departments, and their family and friends: study protocol for intervention development and a pilot randomised controlled trial. BMJ Open. 2015;5:e009040.
26. Noble AJ, Morgan M, Virdi C, Ridsdale L. A nurse-led self-management intervention for people who attend emergency departments with epilepsy: the patients' view. Journal of Neurology. 2013;260(4):1022–30.
27. Ridsdale L, Virdi C, Noble AJ, Morgan M. Explanations given by people with epilepsy for using emergency medical services: a qualitative study. Epilepsy Behaviour. 2012;25:529–33.
28. Ridsdale L, Kwan I, Cryer C. The effect of a special nurse on patients' knowledge of epilepsy and their emotional state. Epilepsy Evaluation Care Group. Br J Gen Pract. 1999;49(441):285-9.
29. Ridsdale L, Kwan I, Cryer C, Group ECE. Newly diagnosed epilepsy: Can nurse specialists help? A randomized controlled trial. Epilepsia. 2000;41(8):1014-9.
30. Moran N, Poole K, Bell G, Solomon J, Kendall S, McCarthy M, et al. Epilepsy in the United Kingdom: seizure frequency and severity, anti-epileptic drug utilization and impact on life in 1652 people with epilepsy. Seizure. 2004;13(6):425-33.
31. Ashworth M, Seed P, Armstrong D, Durbaba S, Jones R. The relationship between social deprivation and the quality of primary care: a national survey using indicators from the UK Quality and Outcomes Framework. Br J Gen Pract. 2007;57(539):441-8.
32. Morrish PK. Inadequate neurology services undermine patient care in the UK. Bmj. 2015;350:h3284.
33. Thornton S. Beyond rhetoric: we need a strategy for patient involvement in the health service. Bmj. 2014;348:g4072.
34. House of Commons Committee of Public Accounts. Financial sustainability of the NHS: 43 2016–17, HC 887 2017 2017 [Available from: publications.parliament.uk/pa/cm201617/cmselect/cmpubacc/887/887.pdf.
35. Grainger R, Pearson M, Dixon P, Devonport E, Timoney M, Bodger K, et al. Referral patterns after a seizure admission in an English region: an opportunity for effective intervention? An observational study of routine hospital data. BMJ open. 2016;6(1):e010100.
36. Blank L, Baxter S, Baird W, Reuber M. Understanding referral patterns to an epilepsy clinic: professional perceptions of factors influencing the referral of older adults. Seizure. 2013 22(9):698-702.

For peer review only

For peer review only - http://bmjopen.bmj.com/site/about/guidelines.xhtml

37. Ziso B, Dixon PA, Marson AG. Epilepsy management in older people: Lessons from National Audit of Seizure management in Hospitals (NASH). Seizure. 2017 50:33-7.
38. Alderwick H, Dunn P, McKenna H, Walsh N, Ham C. Sustainability and transformation plans in the NHS: How are they being developed in practice? : The King's Fund; 2016 [Available from: https://www.kingsfund.org.uk/sites/default/files/field/field_publication_file/STPs_in_NHS_Kings_Fund_Nov_2016.pdf.
39. NICE. Epilepsy in adults: Briefing Paper: NICE; 2012 [Available from: nice.org.uk/guidance/qs26/documents/epilepsy-in-adults-briefing-paper2.
40. National Clinical Guideline Centre. Pharmacological Update of Clinical Guideline 20. The Epilepsies: The Diagnosis and Management of the Epilepsies in Adults and Children in Primary and Secondary Care London: National Clinical Guideline Centre (UK); 2012 [Available from: ncbi.nlm.nih.gov/pubmedhealth/PMH0068980.
41. Imison C, Curry N, Holder H, Castle-Clarke S, Nimmons D, Appleby J, et al. Shifting the balance of care: great expectations. Research Report: Nuffield Trust; 2017 [Available from: https://www.nuffieldtrust.org.uk/files/2017-02/shifting-the-balance-of-care-report-web-final.pdf.
42. Day J. Care Given to Patients Treated for Epileptic Seizure by the London Ambulance Service. London: London Ambulance Service Clinical Audit and Research Unit; 2011.
43. Dickson JM, Taylor LH, Shewan J, Baldwin T, Grünewald R, Reuber M. Cross-sectional study of the prehospital management of adult patients with a suspected seizure (EPIC1). BMJ Open. 2016;6:e010573.
44. Marks P, Daniel T, Afolabi O, Spiers G, Nguyen-Van-Tam J. Emergency (999) calls to the ambulance service that do not result in the patient being transported to hospital: an epidemiological study. Emergency Medicine Journal. 2002;19:449–52.
45. Burrell L, Noble A, Ridsdale L. Decision-making by ambulance clinicians in London when managing patients with epilepsy: a qualitative study. Emergency Medicine Journal. 2013;30:236–40.
46. Noble A, Snape D, Goodacre S, Jackson M, Sherratt F, Pearson M, et al. Qualitative study of paramedics' experiences of managing seizures: a national perspective from England. BMJ Open. 2016;6:e014022.
47. Sherratt F, Snape D, Goodacre S, Jackson M, Pearson M, Marson A, et al. Paramedics' views on their seizure management learning needs: a qualitative study in England. BMJ Open. 2017;7:e014024.
48. Snooks H, Kearsley N, Dale J, Halter M, Redhead J, Cheung W. Towards primary care for non-serious 999 callers: results of a controlled study of "Treat and Refer" protocols for ambulance crews. Quality and Safefy in Health Care. 2004;13:435–43.
49. House of Commons Health Committee. Winter pressure in accident and emergency departments, 2016 HC 277 2016 [Available from: publications.parliament.uk/pa/cm201617/cmselect/cmhealth/277/277.pdf.
50. NHS. The NHS Long Term Plan 2019 [Available from: https://www.longtermplan.nhs.uk/.
51. NHS England. Transforming urgent and emergency care services in England: Clinical models for ambulance services. Urgent and Emergency Care Review Programme Team. 2015 [Available from: nhs.uk/NHSEngland/keogh-review/Documents/UECR-ambulance-guidance-FV.PDF.
52. Michie S, West R, Campbell R, Brown J, Gainforth H. ABC of Behaviour Change Theories. UK: Silverback Publishing; 2014.
53. Fisher JD, Freeman K, Clarke A, Spurgeon P, Smyth M, Perkins GD, et al. Patient safety in ambulance services: a scoping review. 2015.
54. Turner J, Coster J, Chambers D, Phung V-H, Knowles E, Bradbury D, et al. What evidence is there on the effectiveness of different models of delivering urgent care? A rapid review. 2015.
55. Health & Social Care Information Centre. Ambulance Services, England 2014-15. 2015 [Available from: digital.nhs.uk/catalogue/PUB17722.
56. Frostholm L, Fink P, Christensen K, Toft T, Oernboel E, Olesen F, et al. The patients' illness perceptions and the use of primary health care. Psychosomatic Medicine 2005;67(6):997-1005.

For peer review only - http://bmjopen.bmj.com/site/about/guidelines.xhtml

57. Lowe R, Porter A, Snooks H, Button L, Evans B. The association between illness representation profiles and use of unscheduled urgent and emergency health care services. British Journal of Health Psychology. 2011;16(4):862-79.
58. Martin R, Leventhal H. Symptom perception and health care-seeking behavior. . In: Raczynski J, Leviton L, editors. Handbook of clinical health psychology: Vol 2 Disorders of behavior and health. Washington, DC, US: American Psychological Association.; 2004. p. 299-328.
59. Ross J, Stefan H, Schäuble B, Day R, Sander J. European survey of the level of satisfaction of patients and physicians in the management of epilepsy in general practice. Epilepsy & Behaviour. 2010;19(1):36-42.
60. Booker M, Purdy S, Shaw A. Seeking ambulance treatment for 'primary care' problems: a qualitative systematic review of patient, carer and professional perspectives. BMJ Open. 2017;7:e016832.
61. Whitehead K, Kandler R, Reuber M. Patients' and neurologists' perception of epilepsy and psychogenic nonepileptic seizures. Epilepsia. 2013;54(4):708–17.
62. Thompson R, Linehan C, Glynn M, Kerra MP. A qualitative study of carers' and professionals' views on the management of people with intellectual disability and epilepsy: A neglected population. Epilepsy & Behaviour. 2013;28(3):379-85.
63. Dickson JM, Rawlings GH, Grünewald RA, Miles K, Mack C, Heywood T, et al. An alternative care pathway for suspected seizures in pre-hospital care: a service evaluation. British Paramedic Journal. 2017;2(2):22-8.
64. Peterson CL, Walker C, Coleman H. 'I hate wasting the hospital's time': Experiences of emergency department admissions of Australian people with epilepsy. Epilepsy & Behavior. 2019;90:228-32.
65. de Bekker-Grob EW, Ryan M, Gerard K. Discrete choice experiments in health economics: a review of the literature. Health economics. 2012;21(2):145-72.
66. Clark MD, Determann D, Petrou S, Moro D, de Bekker-Grob EW. Discrete choice experiments in health economics: a review of the literature. Pharmacoeconomics. 2014;32(9):883-902.
67. Lancaster KJ. A new approach to consumer theory. Journal of political economy. 1966;74(2):132-57.
68. Greene JC, Caracelli VJ, Graham WF. Toward a conceptual framework for mixed-method evaluation designs. Educational Evaluation and Policy Analysis. 1989;11:255–74.
69. NHS England. D04 Neurosciences 2017 [Available from: https://www.england.nhs.uk/wp-content/uploads/2013/06/d04-neurosci-spec-neuro.pdf.
70. Association of British Neurologists. Acute Neurology services survey 2017. 2017 [Available from: https://www.theabn.org/media/Documents/Acute%20Neurology/ABN%20acute%20neurology%20survey%20final%2013%20March%202017.pdf
71. Brain and Spine Foundation. List of Neurocentres in the UK. [Available from: https://www.brainandspine.org.uk/wp-content/uploads/2018/03/BSF_List-of-Neurocentres-in-the-UK.pdf
72. Health and Social Care Information Centre. Summary Report 7, Hospital Accident and Emergency Activity, 2016-17 2017 [Available from: digital.nhs.uk/media/33252/Hospital-Accident-and-Emergency-Activity-2016-17-Tables/default/acci-emer-atte-eng-2016-17-data.
73. Coast J, Al-Janabi H, Sutton EJ, Horrocks SA, Vosper AJ, Swancutt DR, et al. Using qualitative methods for attribute development for discrete choice experiments: issues and recommendations. Health economics. 2012;21(6):730-41.
74. Vass C, Rigby D, Payne K. The role of qualitative research methods in discrete choice experiments: a systematic review and survey of authors. Medical Decision Making. 2017;37(3):298-313.
75. Mays N, Pope C. Rigour and qualitative research. British Medical Journal. 1995;311:109-12.

For peer review only - http://bmjopen.bmj.com/site/about/guidelines.xhtml

76. Tong A, Sainsbury P, Craig J. Consolidated criteria for reporting qualitative research (COREQ): A 32-item checklist for interviews and focus groups. International Journal for Quality in Health Care. 2007;19(6):349-57.
77. Powell G, Holmes EA, Plumpton CO, Ring A, Baker GA, Jacoby A, et al. Pharmacogenetic testing prior to carbamazepine treatment of epilepsy: patients' and physicians' preferences for testing and service delivery. British journal of clinical pharmacology. 2015;80(5):1149-59.
78. Kralj-Hans I, Goldstein LH, Noble AJ, Landau S, Magill N, McCrone P, et al. Self-Management education for adults with poorly controlled epILEpsy (SMILE (UK)): a randomised controlled trial protocol. BMC neurology. 2014;14(1):69.
79. Ritchie J, Spencer L, O'Connor W. Carrying out qualitative analysis In: Ritchie J, Lewis J, editors. Qualitative Research Practice. New York: Sage Publications; 2003. p. 219–62.
80. Spencer L, Ritchie J, O'Connor W, Morrell G, Ormston R. Analysis in Practice. . In: Ritchie J, Lewis J, McNaughton Nicholls C, Ormston R, editors. Qualitative Research Practice: A Guide for Social Science Students & Researchers. 2nd Edition ed. London: Sage; 2014.
81. Epilepsy Action. A Critical Time for epilepsy in England. A study of epilepsy service provision in England Leeds: British Epilepsy Association; 2013.
82. Bridges JF, Hauber AB, Marshall D, Lloyd A, Prosser LA, Regier DA, et al. Conjoint analysis applications in health—a checklist: a report of the ISPOR Good Research Practices for Conjoint Analysis Task Force. Value in health. 2011;14(4):403-13.
83. House of Commons Hansard. Mental Health and NHS Performance, 9 January 2017, 619. 2017 [Available from: hansard.parliament.uk/commons/2017-01-09/debates/A0C1CB51-3E77-4FD0-87D9AD36C2C11CE5/MentalHealthAndNHSPerformance.
84. Hahn GJ, Shapiro SS. A cataloging and computer program for the design and analysis of orthogonal symmetric and asymmetric fractional factorial experiments. Schenectady, NY: General Electric Research and Development Center; 1966.
85. Street DJ, Burgess L. The construction of optimal stated choice experiments: Theory and methods. London: John Wiley & Sons; 2001.
86. Ericsson KA, Simon HA. Protocol analysis: Verbal reports as data: the MIT Press; 1984.
87. Health and Social Care Information Centre. Quality and Outcomes Framework – Prevalence, Achievements and Exceptions Report, England, 2016-17. 2017 [Available from: https://files.digital.nhs.uk/publication/c/r/qof-1617-rep.pdf.
88. Lancsar E, Louviere J. Conducting discrete choice experiments to inform healthcare decision making: a user's guide. Pharmacoeconomics. 2008;26(8):661-77.
89. Orme B. Interpreting the results of conjoint analysis, getting started with conjoint analysis: strategies for product design and pricing research. Madison, Wis: Research Publishers LLC; 2010.
90. Watson V, Becker F, de Bekker-Grob E. Discrete choice experiment response rates: A meta-analysis. Health economics. 2017;26(6):810-7.
91. Hole AR. Modelling heterogeneity in patients' preferences for the attributes of a general practitioner appointment. Journal of Health Economics. 2008 27(4):1078-94.
92. James Lind Alliance. JLA Guidebook 2019 [Available from: http://www.jla.nihr.ac.uk/jla-guidebook/.
93. INVOLVE. Briefing notes for researchers: involving the public in NHS, public health and social care research Eastleigh: INVOLVE; 2012 [Available from: http://www.invo.org.uk/wp-content/uploads/2012/04/INVOLVEBriefingNotesApr2012.pdf
94. NIHR. TSC SSC Guidance 2016 [Available from: https://www.nihr.ac.uk/funding-and-support/documents/funding-for-research-studies/how-to-apply/TSC-SSC-Guidance-April-2016.pdf.
95. Craig P, Dieppe P, Macintyre S, Michie S, Nazareth I, Petticrew M. Developing and evaluating complex interventions: the new Medical Research Council guidance. Bmj. 2008;337:a1655.

For peer review only - http://bmjopen.bmj.com/site/about/guidelines.xhtml

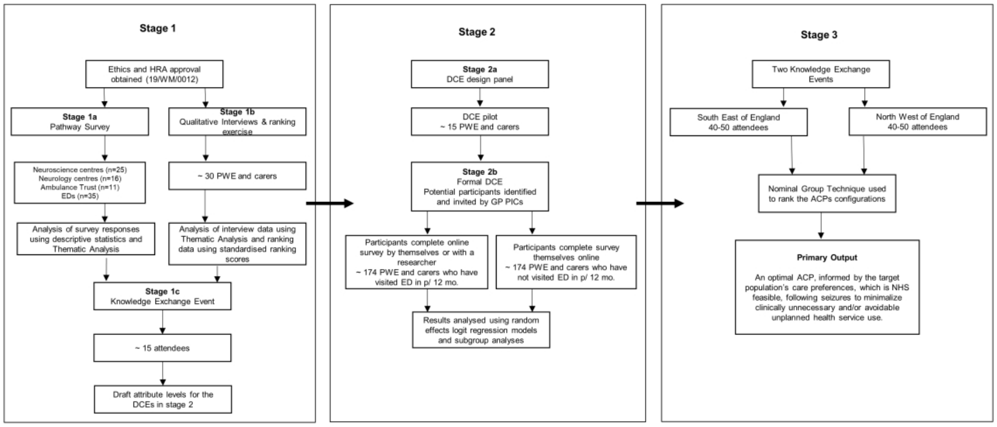

Caption : Figure 1 Research project process diagram Notes: HRA, Health Research Authority; EDs, Emergency Departments; PWE, People with Epilepsy; DCEs, Discrete Choice Experiments; ACP/s, Alternative Care pathway/s

338x190mm (300 x 300 DPI)

Supplementary File 1: Interview Topic Guide

Topics	Questions
**Introduction**	• Thank you very much for participating in this study. We are trying to find out more about how people with epilepsy would like to be cared for when they have a seizure and their views of the emergency services to contribute to the development of services. So I would very much like to hear about your own experiences.<br>• I wondered if you'd share with me your experiences with emergency services that may have led you to being interested in this study?
**Carers**	• Do you have a someone who helps with your epilepsy? (If yes, move on to support person questions. **If no, skip to next section**.)<br>◦ *(Prompts if needed: What is their relationship to you? i.e., family member, friend, paid carer? Do they live with you or somewhere else? How long have you known each other? How often would you usually see each other or have contact?)*<br>• How often have they been with you when you've had seizure in last 12 months?<br>• How confident do they feel in knowing what to do when a seizure happens?<br>◦ *Prompts if needed: Motives for contacting emergency services? Do you contact them every time after a seizure? What makes you feel more confident?*
**Seizures**	Perhaps first you could tell me about your seizures…<br>• What is your impression of how many seizures you have? *(Prompt: i.e., in the last year, month, year?)*<br>• How would you describe your seizures? *(Prompt: what kind do you usually have?)*<br>• How long have you been diagnosed with epilepsy?<br>• **<u>Medication for seizures:</u>** What do you take for your seizures? Are they helpful?<br>• How do you feel about managing your seizures? *(Prompt: do you have any strategies for managing your seizures?)*<br>• Are there any triggers or warnings before the onset of your seizures? *(Prompt: like an aura?)*<br>• How many times have emergency services been called in the past 12 months? How many of those times have you gone to A&E?
**Decision Making**	• I'm interested in your thoughts about the decisions that are made about your care following a seizure.<br>• Firstly, how often would you have seizures in **public** versus at **home**?<br>**Public place:**<br>◦ Can you think of a recent example and tell me where this was?<br>◦ Were you by yourself or with others (who?)

For peer review only

	• Was an ambulance called? What did they do? (i.e., take to A&E) Did you have any chance to discuss where you would like to go?<br>• Have you ever not been taken to A&E after seizure in a public place? *(Prompt: tell me more about what happened then. i.e., who was present, was this what you wanted?)*<br>• Do you have any emergency devices, or a medic alert jewellery?<br>**Home:**<br>• What do you do if a seizure happens when you are alone and no one else is around?<br>• Have **you** called an ambulance **for yourself** when you've had a seizure? If so, can you tell me a bit more about how you found this? How do you feel about the decision on whether or not to go to A&E? When **someone else** has called an ambulance after you've had a seizure, who typically makes the decision to call? Do you know if any alternatives to A&E were discussed? Who would typically make the decision for you to go to A&E?
**Emergency Services / A&E**	• Can you tell me more about what your general experiences of **Emergency Services** for your epilepsy?<br>• *(Prompts if needed: Where are you when the seizure(s) happen (situation and circumstances)? Do you go usually go to A&E? Who makes decision/organised E.g., carer/relative, other lay people, ambulance staff, police, etc. Is this usually what you would want?)*<br>• When was the last time you used emergency services and didn't use A&E? What happened?<br>• What kinds of things might change how you use emergency services? (i.e., such as location, seizure type)<br>• Can you tell me about the last time you went to **A&E** for your epilepsy?<br>• *Prompts if needed: Why did you go to A&E? Who made decision/organised E.g., carer/relative, other lay people, ambulance staff, police, etc.*<br>• Would you say this is typical for you?<br>• *Was this what you would have wanted?*
**Views of A&E**	If you had to use emergency services again, are there any changes that you would like to see in the way this service is provided? (Prompt if needed: and how about A&E?)<br>• What do you believe is most helpful about going to A&E? *E.g., Reassurance, confidence, treatments available, reduce burden on others. (write down)*<br>• Do you see there being anything negative about going to A&E? *E.g., Time, not necessary, unnecessary tests and examinations, disrupted rest, don't like hospitals/being patient, etc. (write down)*<br>• What has been your experience of the link-up/ communication between A&E and your usual care provider?<br>• Have your usual care providers been informed of A&E visits?<br>• Have you been contacted and offered any extra support because you had been to A&E?

Alternatives to A&E	
	• When you have had a seizure and contact has been made with emergency services (i.e., 999/paramedics/111), have you ever been offered an alternative to A&E? If so, what?<br>• If you weren't taken to A&E, where would you like to be taken once your seizure had finished? What health professional would you like to see at the time? What kind of follow-up would you like to have?<br><br>Are you familiar with something called an Urgent Treatment Centre?<br>*Extra Explanation, only if needed:*<br>*The term Urgent Treatment Centres is a relatively new one, but it has been suggested as a potential alternative to A&E. To give you a feel of what they are, the idea is that: -They will be open at least 12 hours a day, 7 days a week. Staffed by GPs and nurses. -Be able to issue prescriptions and have access to some common, but basic equipment, such as ECGs to test heart function and in some cases X-ray machines.*<br>• If yes, have you ever been to one before?<br>• If no, you might be more familiar with terms like 'walk-in centres' and 'minor injury units'. These are to be relabelled urgent treatment centres. *-How many there are located near you, and where they are, varies. Some are located next to GP practices, some are on hospital sites*<br>• What might be your expectations of such a centre be?<br>• Would you have any concerns about this? If so, what kind?<br>• Would anything change how you felt about this ACP? Ie., seizure type, recent changes in meds<br><br>Another option that is being considered instead of always taking people with epilepsy to A&E following a seizure, is for the person to be left at home or taken home if they were out. They would then be telephoned within say 24 hours by an epilepsy nurse specialist. The nurse would be phoning to see how the person was recovering and whether needed any additional support, such as a change in medication, advice or to be booked in to see a neurologist.<br>*Extra Explanation, only if needed/not seen an ENS before:*<br>*-An epilepsy nurse is a **registered nurse who typically has extra experience or qualifications in neurology.** Their role varies but they can provide information regarding epilepsy and its management, they can help monitor and change medication, order tests and act as a first point of contact for patients and GPs. They advise people in a variety of settings, including in outpatient clinics and over the phone.*<br>• What might be your expectations be over this sort of alternative to being taken to A&E?<br>• Would you have any concerns about this? If so, what kind?<br>• Would anything change how you felt about this ACP? Ie., seizure type, recent changes in meds

For peer review only

**Discrete Choice**	• Complete Discrete Choice / Ranking Exercise
**Characteristics**	Now I have some final short-answer questions to run through about you…<br>• What is your Date of birth?<br>• **<u>Highest level of completed education?</u>** (no formal qualification, 1-4GSCE, 5 or more GSCE, Apprenticeship, 2 or more A-levels, First of higher degree/professional qualification/equivalent higher education, vocational/work-related training).<br>• **<u>Ethnicity?</u>** (i.e., white, mixed, Asian/Asian British, Black or Black British, Chinese or other ethnic group).<br>• **<u>Household:</u>** What are your living arrangements? (living with others, living alone)<br>• **<u>Other conditions:</u>** Have you ever been diagnosed with a learning disability? Do you have any mobility difficulties?<br>**<u>Healthcare Professionals:</u>** Who would you contact if anything changes with your epilepsy? Have you seen them in the past 12 months?<br>• Have you seen any other people for help with your epilepsy within the last 12 months? E.g., neurologist, epilepsy nurse, GP with specialist interest in epilepsy, learning disability psychiatrist, paediatrician, neurosurgeon.
**Closing**	• Is there anything you'd like to mention about your experience of emergency services for seizure care that we haven't already discussed?<br>• Thank you very much for your time and for answering my questions. There will be a results report available at the end of the study – would you like to receive a copy?<br>• Do you have any final questions?

**Ranking exercise**	**We are keen to understand what type of care people would prefer when they have a seizure.**<br><br>**Here are some factors** that you have mentioned / or we think may be important …**<br><br>*Interviewer displays cards (Appendix 1) in a random order in front of the participant.*<br><br>Looking at these cards:<br><br>1. **Is there anything missing?** Are there any other factors what would affect your choice?<br><br>*Interviewer writes additional factors on blank cards and places them alongside the pre-defined cards in front of the participant.* ***Record any self-nominated factors on the interview record sheet (Appendix 2).***<br><br>2. **Which are the most important factors in your opinion?** Can you pick up all the cards that would most likely affect your decision?

For peer review only

	Explain that these factors can have been experienced, or not; and include any that were self-nominated in this exercise. Clear the remaining cards from the table.<br><br>**3. Now, can you rank these in order of most important to least important?**<br><br>*Check the order the interviewer places the cards is i.e. So, you think 'X' at the top is the most important? Encourage the participant to "think aloud" and explain their ranking. If participants find it hard to rank certain factors, or align them side by side, explore why.* ***Record the rank scores on the interview record sheet.***

For peer review only

Appendix 1: Show cards

**Where the ambulance takes you**<br>Where the ambulance takes you (e.g. A&E, Urgent treatment centre).
**Care provider**<br>The healthcare professional responsible for your care when you get there.
**Waiting time**<br>How long you have to wait before you see the healthcare professional.
**Tests**<br>The type of tests the health professional could carry out immediately if needed
**Follow-up**<br>Referral to see a health professional with specialist training in epilepsy

For peer review only

Appendix 2: Ranking exercise interview record sheet

Participant ID: _ _ /_ _ / _ _

Interviewer ID _ _

**Factor***	Rank Results (insert 1-5)<br>1 = most important<br>5 = least important<br>X = not selected
Anything Else? **Write here:**	
Anything Else? **Write here:**	
Anything Else? **Write here:**	
Anything Else? **Write here:**	
Anything Else? **Write here:**	
Anything Else? **Write here:**	
Where the ambulance takes you	
Care provider	
Waiting time	
Tests	
Follow-up	

For peer review only

Supplementary File 2 Illustration of a possible vignette and Discrete Choice Experiment choice that could be used

Question 1
Imagine you have a seizure in a public place. It lasted no more than usual and stopped by itself. You are tired and need to sleep. Someone called an ambulance and the paramedic is with you. Which of the following options would you prefer to happen next?

	Option A	Option B
**Where**<br><br>*Where the ambulance takes you*	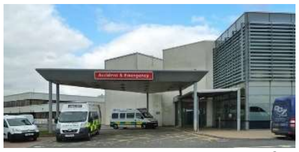<br>***Accident and Emergency (A&E)***	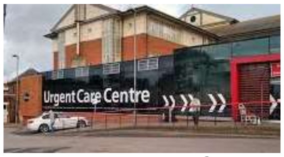<br>***Urgent Treatment Centre***
**Care provider**<br><br>*The health care professional responsible for your care when you get there*	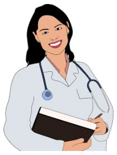<br>***Consultant doctor in emergency medicine***	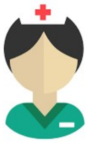<br>***Emergency nurse practitioner***
**Waiting time**<br><br>*How long you have to wait before you see the health care professional*	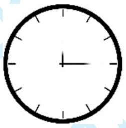<br>***4 hours***	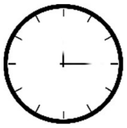<br>***1 hour***
**Tests**<br><br>*The type of tests the health professional could carry out immediately if needed*	***Advanced***<br>(examples...)	***Basic***<br>(examples...)
**Follow-up**<br><br>*The number of people referred to see a health professional with specialist training in epilepsy*	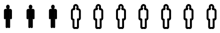<br>***3 in 10 people referred to specialist epilepsy services***	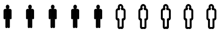<br>***5 in 10 people referred to specialist epilepsy services***
**Which service would you prefer?**	☐	☐

For peer review only

BMJ Open

Developing patient-centred, feasible alternative care for adult emergency department users with epilepsy: Protocol for the mixed-methods observational 'Collaborate' project.

Journal:	*BMJ Open*
Manuscript ID	bmjopen-2019-031696.R2
Article Type:	Protocol
Date Submitted by the Author:	17-Sep-2019
Complete List of Authors:	Noble, Adam; University of Liverpool, Department of Health Services Research<br>Mathieson, Amy; University of Liverpool, Department of Health Services Research<br>Ridsdale, Leone; King's College London, Department of Basic and Clinical Neuroscience, Institute of Psychiatry, Psychology & Neuroscience<br>Holmes, EA; Centre for Health Economics & Medicines Evaluation<br>Morgan, Myfanwy; King's College London, Institute of Pharmaceutical Science<br>McKinlay, Alison; King's College London, Basic & Clinical Neuroscience<br>Dickson, Jon; The University of Sheffield, Academic Unit of Primary Medical Care<br>Jackson, Mike; North West Ambulance Service NHS Trust<br>Hughes, Dyfrig; Bangor University, Centre for Health Economics and Medicines Evaluation<br>Goodacre, Steve; University of Sheffield, Medical Care Research Unit<br>Marson, Anthony; University of Liverpool, Department of Molecular and Clinical Pharmacology
**Primary Subject Heading**:	Neurology
Secondary Subject Heading:	Emergency medicine, Health economics, Health services research, Qualitative research
Keywords:	Epilepsy < NEUROLOGY, ACCIDENT & EMERGENCY MEDICINE, Organisation of health services < HEALTH SERVICES ADMINISTRATION & MANAGEMENT, HEALTH ECONOMICS, QUALITATIVE RESEARCH

SCHOLARONE™
Manuscripts

TITLE: Developing patient-centred, feasible alternative care for adult emergency department users with epilepsy: Protocol for the mixed-methods observational 'Collaborate' project.

AUTHORS: Adam J. Noble,[1] Amy Mathieson,[1] Leone Ridsdale,[2] Emily Holmes,[3] Myfanwy Morgan,[4] Alison McKinlay,[2] Jon Dickson,[5] Mike Jackson,[6] Dyfrig Hughes,[3] Steve Goodacre,[7] Anthony G. Marson.[8]

AFFILIATIONS:

[1] Department of Health Services Research, University of Liverpool, UK.

[2] Department of Basic and Clinical Neuroscience, King's College London, UK.

[3] Centre for Health Economics & Medicines Evaluation, Bangor University, UK.

[4] Institute of Pharmaceutical Science, King's College London, UK.

[5] Academic Unit of Primary Medical Care, University of Sheffield, UK.

[6] North West Ambulance Service NHS Trust, Bolton, UK.

[7] School of Health and Related Research, University of Sheffield, UK.

[8] Department of Molecular and Clinical Pharmacology, University of Liverpool. UK.

FOR CORRESPONDENCE: Name: Adam Noble PhD; Address: University of Liverpool, Department of Health Services Research, Waterhouse Building, Liverpool, L69 3GB, United Kingdom.; Telephone: +44 151 794 5993; E-mail: adam.noble@liverpool.ac.uk

ABSTRACT

Introduction

Emergency department (ED) visits for epilepsy are common, costly, often clinically unnecessary and typically lead to little benefit for epilepsy management. An 'Alternative Care Pathway' (ACP) for epilepsy, which diverts people with epilepsy (PWE) away from ED when '999' is called and leads to care elsewhere, might generate savings and facilitate improved ambulatory care. It is unknown though what features it should incorporate to make it acceptable to persons from this particularly vulnerable target population. It also needs to be National Health Service (NHS) feasible. This project seeks to identify the optimal ACP configuration.

Methods and analysis

Mixed-methods project comprising 3-linked stages. In Stage 1, NHS bodies will be surveyed on ACPs they are considering and semi-structured interviews with PWE and their carers will explore attributes of care important to them and their concerns and expectations regarding ACPs. In Stage 2, Discrete Choice Experiments (DCE) will be completed with PWE and carers

For peer review only - http://bmjopen.bmj.com/site/about/guidelines.xhtml

to identify the relative importance placed on different care attributes under common seizure scenarios and the trade-offs people are willing to make. The uptake of different ACP configurations will be estimated. In Stage 3, two Knowledge Exchange workshops using a nominal group technique will be run. NHS managers, health professionals, commissioners, and patient and carer representatives will discuss DCE results and form a consensus on which ACP configuration best meets users' needs and is NHS feasible.

Ethics and dissemination

Ethical approval: NRES Committee (19/WM/0012) and King's College London ethics Committee (LRS-18/19-10353). Primary output will be identification of optimal ACP configuration which should be prioritised for implementation and evaluation. A pro-active dissemination strategy will make those considering developing or supporting an epilepsy ACP aware of the project and opportunities to take part in it. It will also ensure they are informed of its findings .

Project registration: researchregistry4723.

Strengths and limitations of this study (5 bullet points; one sentence each, relating to the methods)

- Project seeks to identify the optimal configuration of an 'Alternative Care Pathway' for epilepsy which could minimise clinically unnecessary and/or avoidable unplanned health service use and facilitate improved outcomes.
- The novel co-production approach employed – which will involve stakeholders at each project stage and use Discrete Choice Experiments (DCE) – will allow service users to inform service design in a scientifically robust, non-tokenistic way and could provide a template for service design more generally.
- Whilst most of the work packages will involve stakeholders from across England, patient and carers for the DCE are being recruited from one region. This might limit generalisability.
- The ACP configuration identified will be developed for use in the NHS and so may not be immediately applicable to other health systems. It will also require evaluation via a subsequent project.
- The project's success will depend, in part, on service users from what is a particularly vulnerable population agreeing to participate and representative samples being generated.

For peer review only - http://bmjopen.bmj.com/site/about/guidelines.xhtml

Key words: Epilepsy; Accident & Emergency Medicine; Organisation of health services; Heath economics; Qualitative Research.

List of Key Abbreviations

ACP(s)	Alternative Care Pathway(s)
DCE(s)	Discrete Choice Experiment(s)
ED	Emergency Department
GP	General Practitioner
NASH	National Audit of Seizure Management in Hospitals
NHS	National Health Service
NICE	The National Institute for Health and Care Excellence
NIHR	National Institute for Health Research
PPI	Patient and Public Involvement
PWE	People with Epilepsy
SMILE	Self-management Education for Adults with Epilepsy (Trial)
SSC	Study Steering Committee
UK	United Kingdom

5,269 words.

For peer review only

For peer review only - http://bmjopen.bmj.com/site/about/guidelines.xhtml

INTRODUCTION

Context and drive for health service innovations

The National Health Service (NHS), like other health systems, is being asked to make cost savings, whilst improving care experience, outcomes, and reducing health inequalities.[1, 2] Emergency department (ED) visits and admissions for the ambulatory care sensitive condition epilepsy provide an opportunity where service innovations could help achieve such aims. We describe a mixed-methods project to maximise the likelihood of such innovations being beneficial to service users and society.

Epilepsy and unplanned service use

With a prevalence of ~1%,[3] epilepsy is the UK's second most common serious neurological disorder. Annually, ≤20% of people with epilepsy (PWE) visit hospital emergency departments (ED).[4-6] They report more anxiety, seizures and perceived epilepsy stigma than those in the wider epilepsy population and live in more deprived areas; [7-12] ~20% have an intellectual disability.[13, 14]

The annual cost to the NHS of these visits in England alone is ~£70-90M.[15-17] Costs are high because half of the visits result in hospital admission.[6, 18-21] An unusually high re-attendance rate also inflates cost; [22, 23] ≤60% of PWE re-attend ED within 12 months.[7]

Most PWE visiting ED do not attend with a life threatening or emergency presentation, (e.g., status epilepticus, first seizure). Rather, projects like our National Audits of Seizure Management in Hospitals (NASH) [13, 14] show that most have known, rather than new epilepsy, and present with states not requiring ED's full facilities. Leading presentations include someone who has experienced: i) an uncomplicated seizure in line with their usual presentation; ii) a seizure in public and cannot be 'left at scene'); and iii) a self-terminating seizure, different to their usual presentation.[20]

Unmet needs of PWE visiting ED

Whilst the acute episodes leading PWE to visit ED do not typically require emergency care, the visits can be expressions of the person having received suboptimal ambulatory care and unmet needs. NASH [24], for instance, found that most (~65%) PWE visiting ED are not known to specialist epilepsy services and many were via their usual care provider, seemingly receiving outdated care. For instance, despite focal epilepsy being the most common epilepsy type, most likely to be refractory and often not best treated with the medication sodium valproate, NASH found it to be the most prescribed medication amongst ED attendees.

Coping with life in the context of epilepsy also requires an individual to accept their diagnosis and learn and adopt a range of self-management behaviours to prevent seizures and manage consequences. PWE visiting ED though and their significant others appear to

For peer review only - http://bmjopen.bmj.com/site/about/guidelines.xhtml

have less knowledge about epilepsy and its management, including seizure first aid.[7, 25-27] They might therefore benefit from enhanced self-management support, like that provided by epilepsy nurses. The reason that knowledge might be low in ED attendees is because there remains no routine course that all PWE can go on to learn about epilepsy (as there are for some conditions). People who have lower education levels appear to fare worst from this situation with them having been found to have the least epilepsy knowledge.[28, 29]

That some PWE in the UK are receiving suboptimal care is well known, with there being longstanding challenges in ensuring that the PWE most in need of specialist care receive it. Whilst trial evidence indicates ~70% of PWE can become seizure free, only ~50% of PWE in the UK currently are,[30] with PWE in socially deprived areas faring the worst.[31] Factors contributing to the challenge are that the UK has a comparatively small specialist workforce [32] and that there is no national, incentivised system to identify those within the epilepsy population that might need stepped-up care. To further complicate matters, GPs, who care for most PWE, have expressed low confidence in managing the condition.

ED visits by PWE can be considered opportunities to intervene. Unfortunately, under current arrangements, going to ED does not typically lead to PWE receiving ambulatory care improvements. Bodies, such as NICE[2], recommend that when seizures are not controlled, a patient should be referred to specialist services (within 4 weeks) since this may improve outcomes, including rendering some seizure free.[33, 34] Most (80%) PWE visiting ED are though not seen by a specialist during their attendance, their usual care providers may not be informed of the attendance [13] and most (60%) are not referred to a specialist for follow-up.

PWE living in the most deprived areas, as well as the elderly, are amongst the least likely to be referred on from ED.[35] The low referral rate may be due to some clinicians holding an incorrect nihilistic view that intractability is inevitable if seizure control is not obtained within a few years of therapy onset. Assumptions about the willingness of certain patient groups to attend clinics may also be being made.[36, 37]

An ‘Alternative Care Pathway’

NHS policies [1, 2, 38] and publicity given to NASH’s findings [15, 39] created momentum to reduce visits for seizures and enhance patient outcomes. NICE [40] found no evidence on how to do this. However, one idea gaining traction is for ambulance services to assume a greater role.[41] Most (~90%) people visiting ED for seizures have been transported there by an emergency ambulance.[13, 14, 21]

Data from some regional ambulance services on conveyance rates for seizures has been published. It indicates ambulance staff are recommending conveyance of nearly every person they attend for a suspected seizure to ED,[42-44] despite most not demonstrating a clinical need (e.g., seizures have self-terminated before ambulance arrival in ~90% of

For peer review only - http://bmjopen.bmj.com/site/about/guidelines.xhtml

cases),[43] One reason for this is paramedics lack access to alternatives.[45-47] There is a vision therefore of what could help: ambulance service access to some form of 'Alternative Care Pathway' (ACP) whereby those seeking help for an epileptic seizures judged not to require ED are cared for within less costly, alternative environments.

The exact nature of the ACP is not clear. Different regions and services are considering different configurations and doing so in an uncoordinated way. ACPs being considered include paramedics transporting patients' home or to an urgent treatment centre rather than ED. Others involve paramedics leaving patients at scene with the offer of a telephone call from a nurse or general practitioner with a specialist interest in epilepsy within 24, 48 or 72 hours. A possible benefit of this latter configuration is health inequalities could be reduced by introducing a mechanism by which all PWE 'in need' are brought to specialists' attention.

ACPs are not new [48] and paramedics have not been obliged to transport patients to ED since ~1997. The ambitions of ACPs have though increased and there is significant support within the UK policy realm to expand the number of conditions covered by them as a means of managing demands on hospital services (e.g. [49-51]).

ACPs within the ambulance have largely come about due to the 'ISLAGIATT' ('it seemed like a good idea at the time') principle,[52] rather than with reference to a behaviour change theory. Evidence on the utility of ACPs is though generally positive.[48, 53-55] In their review of potential revisions to the urgent and emergency care system, the Nuffield Trust identified greater ambulance/paramedic triage in the community as having the most the positive evidence of effectiveness.[41] Paramedics appear willing and safely able to use ACPs when trained and for some ACPs there is evidence of cost-savings and greater patient satisfaction.[53, 54]

In the case of epilepsy, qualitative research [45-47] provides the beginnings of a theoretical basis for the use of an ACP in epilepsy with the mechanisms by which it could make a difference being that it may: increase awareness and likelihood that paramedics will consider non-conveyance and referral pathways as an option in appropriate cases; increase paramedics' clinical knowledge of how to make appropriate non-conveyance decisions; increase paramedics' knowledge of alternative care providers that are acceptable to service users; and increase paramedics' confidence about making a non-conveyance decision and reducing anxiety about risk.

Developing an ACP for epilepsy

An ACP for epilepsy holds potential. As a team of health care professionals, researchers and service user representatives with expertise in epilepsy and urgent care, our ultimate goal is therefore to evaluate the most promising ACP and use the evidence to transform service organization nationally. However, we cannot currently do this because the way in which ACPs

For peer review only - http://bmjopen.bmj.com/site/about/guidelines.xhtml

are being developed means it is not known which of the many ACPs has the potential to be most effective and could be justified for selection.

Specifically, ACPs are being developed largely ‘top-down’, with the patient voice being absent. The nature and content of ACPs may not therefore align with what patients/carers would consider to meet their needs. Evidence suggests decisions to access healthcare services can be informed by how a patient/carer perceives their situation [56-58] and there can be a mismatch between patients/carers and health professionals’ views, including of what constitutes an ‘emergency’.[59-62] The upshot is the acceptability – a fundamental criterion an intervention needs to satisfy to be positioned to achieve its intended outcome [52] – of the different ACPs to patients and carers is unknown.

To date, only one epilepsy ACP has been reported on.[63] Despite the evaluation revealing positive outcomes, less than 10% of eligible PWE attended to by paramedics were put onto the ACP. The reasons for this low uptake rate were not explored. A possible explanation is that the ACP was not acceptable to PWE.

It has been assumed the target population *does not* want to be conveyed to ED and will readily accept an ACP. It may not be that straightforward. Some PWE certainly express dissatisfaction with being taken to ED.[64] Others though express a need for immediate access to urgent care; with some PWE and their family and friends (to whom care decisions are often delegated to when the patient is unconscious or lacks capacity) being fearful of seizures, including the possibility of brain damage.[26, 27]

CURRENT PROJECT

This 28-month project seeks identify the ACP that should be prioritized for testing/ implementation. It seeks to shape the change that results from the identified momentum so the likelihood of patients and the NHS benefiting are maximised and finite health resources used in an informed, rational way. It will bring patients/carers from the target population, health care professionals and commissioners together to identify which ACP configuration best encapsulates the features important to patients and carers *and* is feasible within the NHS context. The focus will be on the care of adults with epilepsy, rather than children/young people, since discussion to date relating to the use of ACPs and ED care has largely focused on the former (e.g., [24, 63]). As per NICE guidelines for epilepsy,[40] adults are defined as those aged 18 years and older.

The project has 3 linked stages and will use survey techniques, qualitative methods, consensus meetings and Discrete Choice Experiments (DCEs) to achieve its aims (Figure 1).[65, 66] DCEs are an attribute-based survey methodology, underpinned by the theory that any ‘good’ (including a health service) can be described by its constituent characteristics (attributes), and that the extent to which an individual prefers a ‘good’ depends on the levels

For peer review only - http://bmjopen.bmj.com/site/about/guidelines.xhtml

these attributes take.[67] We considered DCEs to provide an efficient, scientifically defensible, and non-tokenistic way of bringing the patient voice into ACP design since they allow a person's stated preferences and priorities to be elicited by presenting them with a hypothetical scenario (e.g., having a seizure at home) and asking them to choose which of two (or more) care options described by a series of attributes they prefer. The process is then repeated with alternative care choices being presented. By varying attribute levels and observing how participants change responses, the importance of attributes and the extent to which they drive preference can be inferred.

Aims

a) Systematically identify ACPs being considered by the NHS for epilepsy and describe extent to which service users have been involved in their design (Stage 1a).
b) Understand patient and carers' decision-making processes for seeking or not seeking ED care, and their concerns and expectations regarding ACPs (Stage 1b).
c) Identify the attributes of post-seizure emergency care that PWE and their carers consider important and identify if this differs by the context in which the seizure occurs (Stage 1b).
d) Determine patient and carer preferences for post-seizure emergency care using DCEs and identify any subgroup differences (Stage 2b).
e) Estimate uptake of different ACPs configurations by patients and subgroup differences (Stage 2b).
f) Hold expert workshops to develop an optimal ACP following seizures, to be prioritised for implementation and evaluation via a subsequent project (Stage 3).

METHODS AND ANALYSIS

This project will use qualitative and quantitative methods (Figure 1). According to Greene et al.'s [68] conceptual framework, the purpose of using a mixed methods approach is 'development' (seeking to use the results from one method to help develop or inform the other method, where development is broadly construed to include sampling and implementation, as well as measurement decisions; Stages 1-2b) and 'expansion' (seeking to extend the breadth and range of inquiry by using different methods for different inquiry components; Stages 2b and 3).

Data collection and analysis for Stages 1a and 1b will be completed concurrently and independent from one another. From Stage 1c onwards, the design will be sequential and dependent. During Stage 2a, the findings from the earlier stages will be integrated to develop the DCE. Following completion of analysis for Stage 3, findings from all the stages will be

For peer review only - http://bmjopen.bmj.com/site/about/guidelines.xhtml

integrated in a narrative form when interpreting and reporting on the project's findings. Particular attention will be given to how the findings from Stage 3 converge and diverge from the qualitative and quantitative findings from the earlier stages.

Stage 1

Stage 1a: Pathway survey

Purpose: Systematically identify ACPs being considered. Will allow ACPs presented within DCEs to include main ones being considered.

Design: Cross-sectional survey of NHS service providers asking respondents whether their service has considered (or implemented) any changes to the management of those with epilepsy/suspected seizures to minimise clinically unnecessary/avoidable ED visits. Respondents will report how change is anticipated to minimise visits and whether service users informed change.

The survey will take ~10 minutes, be piloted and completed online. Survey respondents will be able to indicate if they want to be informed of subsequent project parts.

Recruitment: Invitations to participate, followed by two reminders, will be sent to clinical leads/ directors of England's regional ambulance trusts (n=11), regional neuroscience (~n=25) [69] and neurology centres (~n=16),[70, 71] and a random sample of 25% of its 'Type 1' EDs [72] (n=35), stratified by geographical area and size (attendances).

Analysis: Characteristics of responding and non-responding organisations will be compared. To determine the types of ACPs being considered and proportion of organisations considering each, a subset of surveys will be selected. A qualitative researcher, supported by the wider team, will read these to identify recurrent ACP types and collate them into a thematic coding framework. The framework will be applied to the full data set and modified to ensure all ACPs types are captured.

Stage 1b: Qualitative interviews

Purpose: Understand patient and carer decision-making, the important attributes of post-seizure care, and concerns and expectations regarding ACPs. Will permit attribute development to adhere to good practice.[73, 74]

Design: Face-to-face semi-structured interviews will be conducted with PWE, and where possible an informal carer, and shaped by a piloted topic guide (Supplementary File 1).

For peer review only - http://bmjopen.bmj.com/site/about/guidelines.xhtml

Participants will be asked for contextual information about their epilepsy and ED visits. The researcher will explore patients and carers confidence in managing seizures and the decision to call the ambulance services. The influences on ambulance use and ED use when seizures occur in public places will also be examined. Evaluative information will then be sought regarding positive and negative perceptions of the ambulance service and casualty as well as any ACPs experienced. Potential attributes will be identified by describing two known ACPs (i.e., urgent care centres and being taken home) and asking participants for their expectations and concerns.

To help reduce the number of care attributes and improve face validity, participants will rank what they consider to be the five most important care attributes. Interviewer will help recall those mentioned and write them onto show cards.

To promote consistency, interviews will be conducted by one experienced qualitative researcher [AMK]. AMK (PhD), is a university-based qualitative researcher with an interest in health services research but no specialist knowledge of, or involvement in, the ambulance or emergency medical services. Before agreeing to take part, participants will be given a participant information sheet detailing the research. Written informed consent will be obtained [by AMK] before the interviews. No non-participants will be present during the interviews. Interviews will take place at a time and place convenient to the participant. It is anticipated they will last 60-90 minutes.

To promote transparency, meticulous records of the interviews will be kept, interviews audio-taped, transcribed verbatim [75] and reported according to COREQ.[76]

Recruitment: Aim is to capture a comprehensive range of perspectives, and sample size will be determined by data saturation. We anticipate [73, 77] recruiting a purposive sample of ~30 PWE, with or without an informal carer. Table 1 lists the inclusion and exclusion criteria.

Eligible PWE will be identified by two means. Firstly, persons with uncontrolled epilepsy who participated in a recent NIHR funded 'Self-Management Education for adults with Epilepsy' (SMILE) trial in the South-East of England and expressed interest in future projects will sent notification of the study.[78] Secondly, adverts will be circulated to persons affiliated with epilepsy user groups, including Epilepsy Action.. Persons interested in taking part will be asked to contact the research team. To identify carer participants, patients will be asked to choose a significant other/informal carer to take part. Participants will receive a £20 shopping voucher.

Analysis: Analysis will proceed alongside data collection and be based on a Framework approach.[79] This approach ensures each case is fully accounted for and is regarded as particularly helpful for addressing policy questions. The primary aim of analysis will be to

For peer review only - http://bmjopen.bmj.com/site/about/guidelines.xhtml

generate an exhaustive list and description of the salient attributes of care (and levels) identified by participants.

The analytic approach will involve the identification of initial themes that are then grouped into main themes and subthemes. Transcripts will be read and re-read, and coded. At least two members of the research team (AMK, MM, LR) will participate in all data analysis to reduce bias in the identification and interpretation of themes.

A thematic 'chart' will be created to summarise information for each theme to allow cross-case and within-case analysis through a process of constant comparison, with particular attention being paid to deviant cases. Analysis will pay attention to the similarity and differences in salient attributes according to where the seizure occurred (home, public), who was there, who made the 999 call, whether the patient had seen a specialist in the prior 12 months, and whether they self-reported an intellectual impairment. Participant quotations (with minor editing where necessary to preserve anonymity) will presented to illustrate themes and help verify interpretation. QSR International's NVivo 11 qualitative data analysis software [(80)] will be used as a management tool throughout the process.

The attributes nominated during the ranking exercise will be categorised according to the thematic framework, then analysed quantitatively [by EH] using standardised rank scores.

Stage 1c: Knowledge exchange event

Purpose: It is important the DCE elicits views on ACPs that could plausibly be delivered within the NHS. This event will therefore, early in the project, determine feasibility of the attributes and associated levels that have been identified by service users in Stage 1b.

Design: One-day event at which health care professional representatives will be shown draft attributes, levels, and choice sets and asked to deliberate each choice. For instance, should Stage 1b indicate service users consistently say they prefer to be transported home by the ambulance service regardless of distance, then representatives would be asked to what constitutes a feasible distance. Our team has previously used this approach.[(81)]

Recruitment: Around n=15 ambulance staff and neurology service representatives. Persons will be recruited from organisations participating in Stage 1a with the help of a sampling framework to ensure representatives come from geographically diverse areas. Representatives will be reimbursed for travel. Those not able to attend will be able to provide feedback remotely.

Analysis: With attendees' consent, the session will be audio-recorded and field notes taken. Feedback will be used to optimise the attribute levels within the DCEs.

For peer review only - http://bmjopen.bmj.com/site/about/guidelines.xhtml

Stage 2

Stage 2a: Developing the DCE

Purpose: Generate DCE questionnaire to elicit patient and carer preferences for post-seizure care and which meets best practice guidance.[73, 82]

Design: A multidisciplinary panel, chaired by DH, will compose a draft DCE questionnaire using Stage 1 evidence and refine it on the basis of a pilot. The panel will include expertise in DCEs [EH], emergency medicine [SG], neurology [AGM], paramedical science [MJ], general practice [JD], and psychology [AN], as well as having service user representation.

The panel will form a consensus on which restricted number of ACPs best represent the range being considered and are to be valued using the DCE. Using the list of candidate attributes generated from Stage 1b the panel will select 4-6 attributes by which to describe the packages.

In selecting attributes, the panel will favour those prioritised by service users, which are modifiable, capable of being traded and best describe the ACPs.[73] Attributes deemed to be too close to the latent construct of the utility derived from the ACP (e.g., 'satisfaction') will not be selected.

DCEs will be generated for the three most common presentations to ED by PWE. For each, participants will be presented with a seizure scenario vignette and via standardised instructions complete a series of forced, pairwise comparisons to indicate which care packages they would prefer. Vignettes will be developed by the panel based on clinical and lived experience. Supplementary File 2 provides an illustration of what a DCE *might* look like.

Plausible attribute levels will be specified on the basis of findings from Stage 1, expert opinion and relevant literature. A strength of DCEs is the possibility of varying attribute levels to also model preferences within future contexts. Using its expertise and knowledge, the panel will seek to assign levels to attributes to account for major anticipated changes – such as longer ED waiting times.[83]

A fractional factorial design will identify a purposeful subset of options for each DCE, to allow an estimate of the main effect of each attribute independently and selected two-factor interactions, while minimizing the number of paired comparisons participants are asked to make. The profiles will be obtained from a published design catalogue.[84] Binary choices will then be created using the fold-over method, which replaces each attribute level systematically.[85]

Once drafted, the DCE will undergo iterative individual pilot testing with ~15 PWEs and carers from the project's Patient and Public Involvement (PPI) group to check clarity and duration. Pilotees will 'think aloud' [86] when making choices, and asked to consider their

For peer review only - http://bmjopen.bmj.com/site/about/guidelines.xhtml

preferred presentation of attributes (e.g. text, pictograms). Sessions will be audio recorded, notes made and refinements made.

Stage 2b: Formal DCE

Purpose: Determine patient and carer preferences for post-seizure emergency care, estimate uptake of different ACPs configurations, and subgroup differences.

Design: A representative sample of PWE and informal carers who have visited ED in the last 12 months, as well as those at risk, will be sought to complete the finalised DCE questionnaire. PWE who have uncontrolled epilepsy, but who have not visited ED in the last 12 months will be asked to visit an online survey page and complete the DCE questionnaire using their own internet enabled devices. Due to potentially greater social disadvantage, patients who *have* visited ED in the prior 12 months will have the additional option of completing the DCE during a face-to-face appointment with a researcher who will provide an internet enabled device.

To limit burden, participants will complete DCEs relating to just two seizure scenarios (allocated at random). As well as the DCEs, respondents will report on their demographics, epilepsy, knowledge and fear of seizures, and service use. Each participant will receive a £20 shopping voucher.

Recruitment: General Practices in North-West England will identify potential patient participants by searching their epilepsy registers.[(87)] Practices will be recruited with the help of the NIHR Clinical Research Network North West Coast. Practices will send ostensibly eligible patients invitation packs via 'Docmail' and a repeat invite 2 weeks later to those not responding. Patients not wishing to participate will be encouraged to notify the team and detail their reasons. Eligibility criteria are in Table 1.

A definitive sample size calculation depends on the finalized design of the DCE.(88) To permit decisions regarding recruitment to be made in the meantime we anticipated the design and calculated the required sample size by using Orme's[(89)] formula. Full details are provided in Table 2. This indicated 348 PWE (or associated informal carers) would be required, comprising data 174 patients (or associated carers) who have visited ED in the prior 12 months and 174 patients (or associated carers) who have not.

Based on responses to prior health DCEs [(90)] and studies with PWE,[(16, 25)] we anticipate 30-60% of those invited will agree to participate. An average English GP practice [155] will have 24 PWE aged ≥18 on their register who have experienced ≥1 seizure in the prior 12 months,[9] with 10 having visited an ED during that period.[12-14] Governed by the need to recruit a sub-group of 174 PWE who have visited ED in the prior 12 months, 29-58 general practices will be required.

For peer review only - http://bmjopen.bmj.com/site/about/guidelines.xhtml

Analysis: Data for the different seizure vignettes will be analysed separately using an error components logit model that allows for a panel of responses from the same respondent. This will determine the importance of the different care attributes for preference and direction of effect (as indicated by the significance of the coefficients and their size). Comparison between vignettes will depend on attribute and level specification of each experiment. Analyses will be completed with and without respondents failing a 'rationality' test.

Marginal rates of substitution (the rate at which respondents are willing to give up a unit change in one attribute in exchange for a unit change in another while maintaining the same level of utility) will be calculated using each quantitative attribute as the value attribute with bootstrapped confidence intervals (1000 reps).

Preference heterogeneity will be assessed by estimating a mixed logit model, which will include random parameters for the attributes, and interactions between attributes and respondent characteristics.(91) A-priori characteristics of interest are: (1) emergency care for epilepsy in the past 12-months (paramedic and/or ED visit), (2) social deprivation (with participants grouped according to how socially deprived the area is within which they live); (3) whether or not they have seen an epilepsy specialist within the prior 12 months (which they should have according to NICE guidelines [89] given they will all have uncontrolled epilepsy); and (4) whether they or the person they know in the case of carers, self-reports an intellectual disability.

'Utility scores' for different ACP configurations and significantly different populations will be calculated and ranked accordingly. Their uptake under different seizure scenarios will be estimated.

All analyses will be conducted in STATA 11 (StataCorp, College Station, TX, USA).

Stage 3: *Knowledge Exchange workshops*

Purpose: Disseminate Stage 1 and 2 findings to those positioned to develop, fund, support and run ACPs and utilise their expertise to identify which ACP/s favoured by patients and carers is most NHS feasible and should be prioritised for implementation/ evaluation.

Design: One Knowledge Exchange event in South England, one in the North. They will start with presentation of project's findings, including ACP configurations ranked according to service user preference. A Nominal Group Technique, described by the James Lind Alliance, (92) will then be used to hear delegates' views of the ACPs and rank them according to the extent to which they meet users' preferences and are NHS feasible. Facilitators will encourage delegates to consider barriers to change, supply constraints, acceptability of the service to staff, possible cost, and potential to redress health care inequalities.

For peer review only - http://bmjopen.bmj.com/site/about/guidelines.xhtml

Voting will be completed using electronic devices. With attendees' consent, sessions will be audio-recorded, and a qualitative researcher will observe and record field notes.

Recruitment: Aim to have 40-50 health service managers, commissioners, healthcare professionals, and service user representatives at each event. Persons will be recruited mainly from the institutions participating in Stage 1a. Additional attendees will come from commissioning groups and the epilepsy patient groups. The project's PPI group will also be invited. A sampling matrix will be formed to help ensure broadly equal representation of persons from health professional, commissioning and managerial roles.

Analysis: The rank order of the ACPs that results from delegates voting will be described. Depending on findings from the previous stages, the ACPs might have been presented to delegates according to seizure scenario. If so, rank orders for the ACPs for the different scenarios will be determined and compared. As a secondary objective, rank orders for the different representative subgroups will be determined. Transcripts from the events will be thematically analysed to capture delegates' views and justifications for their preferences.

DISCUSSION

Patient and public involvement

This research was driven by a need to consider the views of PWE when designing an ACP for epilepsy. Epilepsy Action, the largest user organisation in the UK, contributed to the design of the research, and facilitated a PPI event with 23 service users to discuss project design and conduct. A PPI group will be established, including PWE and carers. They will be supported by Epilepsy Action who have an active PPI scheme and reimbursed for travel and time according to INVOLVE.[93] The group will contribute as research peers by: providing feedback on research materials; interpretation of findings; piloting the DCE; and being delegates in workshops. The group will be encouraged to help with dissemination, including presenting the findings at the Stage 3 events and teaching.

Ethics and dissemination

Monitoring by an independent Study Steering Committee (SSC) will help to ensure the rights, safety and well-being of participants are the most important considerations. Only persons providing informed consent will participate. The SSC will be composed according to our funder guidelines.[94] Compliance with the principles of Good Clinical Practice and scientific integrity will be managed by the study management team through regular and ad hoc meetings.

For peer review only - http://bmjopen.bmj.com/site/about/guidelines.xhtml

The project will identify what, if any, ACP configuration/s is most acceptable to service users and has the backing of those expected to deliver and support it. To ensure maximum impact those considering an ACP need to be aware of the project, have confidence in its outputs and use its findings. To help ensure this: 1) Representatives from all of the relevant service providers in the UK will be informed of the project (including notifying the leads of England's Sustainability and Transformation Partnerships and Urgent and Emergency Care Vanguards); 2) professional bodies will announce its funding (Acknowledgements); 3) the research team includes appropriate expertise and opinion leaders; and 4) representatives from relevant NHS services will be invited to take part in Stage 1a and 3a.

The project's findings will be disseminated via international conferences and at least four journal manuscripts. The manuscripts will be published open access and non-technical reports will be circulated via NHS network newsletters and Epilepsy Action. Participants will be provided with a copy of the results.

Having identified the ACP, funding will stop but the team would, via a subsequent project seek to implement and evaluate the chosen ACP. The nature of that evaluation will be informed by the state of the NHS closer to the time. The Medical Research Council [95] highlight intervention evaluation takes place in a wide range of settings and many factors can constrain choice of evaluation methods. One trade-off that may need consideration is time until results become available/ demand versus methodological rigour.

For peer review only - http://bmjopen.bmj.com/site/about/guidelines.xhtml

Acknowledgements: The authors would like to thank Epilepsy Action (Angie Pullen, Amanda Stoneman) for the role that they will have in this project. They would like to express appreciation for the contributions from people with epilepsy and their family members and friends who will participate in this study and thank members of the Study Steering Committee (Tom Quinn [chair], Verity Watson, Trevor Baldwin, Nigel Rees, Khalid Hamandi, Daniel Horner, Juliet Bransgrove, and Jayne and Sam Burton). We also acknowledge the International League Against Epilepsy (British Branch), Royal College of Emergency Medicine and the Epilepsy Nurses Association for informing members of the project. The study sponsor is the University of Liverpool (reference: UoL4258; sponsor@liv.ac.uk).

Author Contributions: AJN conceived of the study and designed it together with EH, DH, LR, MM, MJ, JD, SG and AGM. EH planned the statistical analysis for the DCE. AJN and AM wrote the manuscript, with revisions being made by EH, AM, DH, LR, MM, MJ, JD, SG and AGM. All authors read and approved the final manuscript.

Funding statement: This project is funded by the National Institute for Health Research's Health Services and Delivery Research Programme (HS&DR Programme) (project number 17/05/62). The views and opinions expressed herein are those of the authors and do not necessarily reflect those of the University of Liverpool, the HS&DR programme, the NIHR, the NHS, or the Department of Health and Social Care.

Competing interests: None declared.

Patient consent for publication: Not required.

Data statement: This is a protocol. There is no data associated with it.

Ethics approval: This study has been received Health Research Authority approval and favourable ethical opinions from NRES Committee West Midlands - Solihull (19/WM/0012) and King's College London's Psychiatry, Nursing and Midwifery Ethics Committee (LRS-18/19-10353).

For peer review only

Table 1 Participant inclusion and exclusion criteria

Study part	Inclusion criteria	Exclusion criteria
**Stage 1b – Qualitative interviews**		
	▪ Established diagnosis of epilepsy, or an informal carer for someone with epilepsy<br>▪ Age ≥18 years (no upper age limit)<br>▪ Have visited ED in the past 12 months for epilepsy (as reported by the patient)*<br>▪ Able to provide informed consent and communicate in English	▪ Severe current psychiatric disorders (e.g. acute psychosis)<br>▪ Life-threatening medical illness
**Stage 2 – Discrete Choice Experiments**		
**Group 1**		
**Patients**	▪ Clinically confirmed diagnosis of epilepsy (for any duration)<br>▪ Any epilepsy syndrome and any types of focal or generalised seizures<br>▪ Currently being prescribed antiepileptic medication<br>▪ Age ≥18 years (no upper age limit)<br>▪ Have visited ED in the past 12 months for epilepsy<br>▪ Able to provide informed consent and independently complete a questionnaire in English<br>▪ Lives in the North West of England	▪ Severe current psychiatric disorders (e.g. acute psychosis)<br>▪ Life-threatening medical illness<br>▪ Resides within a care or nursing home or of no fixed abode
**Carers**	▪ A significant other to a person with epilepsy (e.g. family member,	▪ Severe current psychiatric disorders (e.g. acute psychosis)

For peer review only - http://bmjopen.bmj.com/site/about/guidelines.xhtml

	friend) who the patient identifies as providing informal support or self-identifies themselves if the patient has a substantial intellectual disability<br>▪ The person with epilepsy they care for has visited ED in the past 12 months<br>▪ Age ≥16 years (no upper age limit)<br>▪ Able to provide informed consent and independently complete a questionnaire in English<br>▪ Lives in the North West of England	▪ Life-threatening medical illness
**Group 2**		
**Patients**	▪ Clinically confirmed diagnosis of epilepsy (for any duration)<br>▪ Any epilepsy syndrome and any types of focal or generalised seizures<br>▪ Currently being prescribed antiepileptic medication<br>▪ Age ≥18 years (no upper age limit)<br>▪ Has *not* visited ED in the past 12 months, but has had a seizure with loss of awareness in this period<br>▪ Able to provide informed consent and independently complete a questionnaire in English<br>▪ Lives in the North West of England	▪ Severe current psychiatric disorders (e.g. acute psychosis)<br>▪ Life-threatening medical illness<br>▪ Resides within a care or nursing home or has no fixed abode
**Carers**	▪ A significant other to a person with epilepsy (e.g. family member, friend) who the patient identifies as providing informal support or self-identifies themselves if the patient	▪ Severe current psychiatric disorders (e.g. acute psychosis<br>▪ Life-threatening medical illness

For peer review only - http://bmjopen.bmj.com/site/about/guidelines.xhtml

For peer review only

has a substantial intellectual disability

- The person with epilepsy they care for has *not* visited ED in the past 12 months, but had a seizure with loss of awareness in this period
- Age ≥16 years (no upper age limit)
- Able to provide informed consent and independently complete a questionnaire in English
- Lives in the North West of England

Notes: ED, emergency department; *Should recruitment prove slower than anticipated, the recruitment criteria will be relaxed to allow people who have had ED or ambulance contact within the last 2 years.

For peer review only

For peer review only - http://bmjopen.bmj.com/site/about/guidelines.xhtml

Table 2 Sample size calculation for Discrete Choice Experiments

**Formula used and factors considered in estimating likely sample size required**
• Orme's formulae: (*n***t***a*)/ *c* must be ≥500, where *n* is number of respondents, *t* number of tasks, *a* number of alternatives per task, and *c* number of analysis cells (for main effects, the largest number of levels for any one attribute).<br>• Anticipated Discrete Choice Experiments (DCEs) to involve participants being asked to make (*t*) 9 separate choices between (*a*) 2 care packages and (*c*) 3 would be maximum attribute levels. Thus, Orme's formula indicated complete data from n=84 needed.<br>• Intend to complete DCEs for three different seizure scenarios, but participants would only complete two, therefore 126 participants required.<br>• To allow for variation in the finalized number of choice tasks and levels we increased this to 150 and further inflated it to 174 to accommodate for ~15% of missing data experienced by prior epilepsy DCEs.<br>• As an objective was to describe and compare care preferences of two subgroups (patients (and associated carers) who have visited an ED in the prior 12 months vs. patients (and associated carers) who have had a seizure in the prior 12 months, but have not visited an ED), 174 patients (and associated carers) who have visited ED in the prior 12 months and 174 patients (and associated carers) who have not, will need to be recruited.<br>• Total sample size, thus n=348.

For peer review only

For peer review only - http://bmjopen.bmj.com/site/about/guidelines.xhtml

Figure Legend

Figure 1: Research project process diagram.

Notes to Figure 1: HRA, Health Research Authority; EDs, Emergency Departments; PWE, People with Epilepsy; DCEs, Discrete Choice Experiments; ACP/s, Alternative Care pathway/s

For peer review only

REFERENCES

1. NHS England. Five Year Forward View 2014 [Available from: https://www.england.nhs.uk/wp-content/uploads/2014/10/5yfv-web.pdf.
2. NHS England. Next Step on the NHS Five Year Forward View 2017 [Available from: england.nhs.uk/wp-content/uploads/2017/03/NEXT-STEPS-ON-THE-NHS-FIVE-YEARFORWARD-VIEW.pdf.
3. Ridsdale L, Charlton J, Ashworth M, Richardson MP, Gulliford MC. Epilepsy mortality and risk factors for death in epilepsy: a population-based study. Br J Gen Pract. 2011;61(586):e271-e8.
4. Hart YM, Shorvon SD. The nature of epilepsy in the general population. II. Medical care. Epilepsy research. 1995;21(1):51-8.
5. Jacoby A, Buck D, Baker G, McNamee P, Graham-Jones S, Chadwick D. Uptake and costs of care for epilepsy: findings from a UK regional study. Epilepsia. 1998;39(7):776-86.
6. Kitson A, Shorvon S. Services for patients with epilepsy: Report of a CSAG committee. Department of Health; 2000.
7. Noble AJ, Goldstein LH, Seed P, Glucksman E, Ridsdale L. Characteristics of people with epilepsy who attend emergency departments: Prospective study of metropolitan hospital attendees. Epilepsia. 2012;53(10):1820–8.
8. Bautista RED, Glen ET, Wludyka PS, Shetty NK. Factors associated with utilization of healthcare resources among epilepsy patients. Epilepsy research. 2008;79(2-3):120-9.
9. Allard J, Shankar R, Henley W, Brown A, McLean B, Jadav M, et al. Frequency and factors associated with emergency department attendance for people with epilepsy in a rural UK population. Epilepsy & Behavior. 2017;68:192-5.
10. Public Health England. The NHS Atlas of Variation in Healthcare: Reducing unwarranted variation to increase value and improve quality. 2016 [Available from: fingertips.phe.org.uk/documents/Atlas_2015%20Compendium.pdf.
11. QIPP/Right Care. The NHS atlas of variation in healthcare 2010 [Available from: fingertips.phe.org.uk/documents/Atlas_2010%20Compendium.pdf.
12. Right Care. The NHS Atlas of Variation in Healthcare: Reducing unwarranted variation to increase value and improve quality. 2011.
13. Pearson M, Marson A, Dixon P, Scott K. National Audit of Seizure Management in Hospitals: St. Elsewhere's Hospital Clinical Report 2014 [Available from: http://www.nashstudy.org.uk/Newsletters/St%20Elsewhere's%20Clinical%20Report%20NASH%202.pdf.
14. Pearson M, Marson A, Dixon P, Billington K. National Audit of Seizure Management in Hospitals St Elsewhere's Clinical Report 2012 [Available from: http://nashstudy.org.uk/Newsletters/St%20Elsewhere%27s%20NASH%20Report.pdf.
15. House of Commons Committee of Public Accounts. Services to people with neurological conditions: progress review London House of Commons; 2015 [Available from: https://publications.parliament.uk/pa/cm201516/cmselect/cmpubacc/502/502.pdf.
16. Ridsdale L, McCrone P, Morgan M, Goldstein L, Seed P, Noble A. Can an epilepsy nurse specialist-led self-management intervention reduce attendance at emergency departments and promote well-being for people with severe epilepsy? A non-randomised trial with a nested qualitative phase. Health Services and Delivery Research 2013;1(9).
17. Dickson JM, Jacques R, Reuber M, Hick J, Campbell MJ, Morley R, et al. Emergency hospital care for adults with suspected seizures in the NHS in England 2007–2013: a cross-sectional study. BMJ open. 2018;8(10):e023352.
18. Bardsley M, Blunt I, Davies S, Dixon J. Is secondary preventive care improving? Observational study of 10-year trends in emergency admissions for conditions amenable to ambulatory care. BMJ open. 2013;3(1):e002007.

For peer review only - http://bmjopen.bmj.com/site/about/guidelines.xhtml

19. Peter A Dixon, Jamie J Kirkham, Anthony G Marson, Pearson MG. National Audit of Seizure management in Hospitals (NASH): results of the national audit of adult epilepsy in the UK BMJ Open 2015;5(3):e007325.
20. Reuber M, Hattingh L, Goulding PJ. Epileptological emergencies in Accident and Emergency: a survey at St James's University Hospital, Leeds. Seizure. 2000;9(3):216-20.
21. Ryan J, Nash S, Lyndon J. Epilepsy in the accident and emergency department--developing a code of safe practice for adult patients. South East and South West Thames Accident and Emergency Specialty Sub-committees. . Emergency Medicine Journal 1998;15.
22. Whiston S, Coyle B, Chappel D. Health needs assessment for long term neurological conditions in North East England. Stockton on Tees: North East Public Health Observatory; 2009.
23. NICE. Services for the diagnosis and management of the epilepsies in adults, children and young people: commissioning guide 2013 [Available from: nice.org.uk/guidance/qs26/resources/services-for-thediagnosis-and-management-of-the-epilepsies-in-adults-children-and-young-people-commissioning-guide-317820637/chapter/3-Assessingservice-levels-for-the-diagnosis-and-management-of-the-epilepsies#ftn.footnote_19.
24. Dixon PA, Kirkham JJ, Marson AG, Pearson MG. National Audit of Seizure management in Hospitals (NASH): results of the national audit of adult epilepsy in the UK. BMJ Open. 2015;5:e007325.
25. Noble AJ, Marson AG, Tudur-Smith C, Morgan M, Hughes DA, Goodacre S, et al. 'Seizure First Aid Training' for people with epilepsy who attend emergency departments, and their family and friends: study protocol for intervention development and a pilot randomised controlled trial. BMJ Open. 2015;5:e009040.
26. Noble AJ, Morgan M, Virdi C, Ridsdale L. A nurse-led self-management intervention for people who attend emergency departments with epilepsy: the patients' view. Journal of Neurology. 2013;260(4):1022–30.
27. Ridsdale L, Virdi C, Noble AJ, Morgan M. Explanations given by people with epilepsy for using emergency medical services: a qualitative study. Epilepsy Behaviour. 2012;25:529–33.
28. Ridsdale L, Kwan I, Cryer C. The effect of a special nurse on patients' knowledge of epilepsy and their emotional state. Epilepsy Evaluation Care Group. Br J Gen Pract. 1999;49(441):285-9.
29. Ridsdale L, Kwan I, Cryer C, Group ECE. Newly diagnosed epilepsy: Can nurse specialists help? A randomized controlled trial. Epilepsia. 2000;41(8):1014-9.
30. Moran N, Poole K, Bell G, Solomon J, Kendall S, McCarthy M, et al. Epilepsy in the United Kingdom: seizure frequency and severity, anti-epileptic drug utilization and impact on life in 1652 people with epilepsy. Seizure. 2004;13(6):425-33.
31. Ashworth M, Seed P, Armstrong D, Durbaba S, Jones R. The relationship between social deprivation and the quality of primary care: a national survey using indicators from the UK Quality and Outcomes Framework. Br J Gen Pract. 2007;57(539):441-8.
32. Morrish PK. Inadequate neurology services undermine patient care in the UK. Bmj. 2015;350:h3284.
33. Thornton S. Beyond rhetoric: we need a strategy for patient involvement in the health service. Bmj. 2014;348:g4072.
34. House of Commons Committee of Public Accounts. Financial sustainability of the NHS: 43 2016–17, HC 887 2017 2017 [Available from: publications.parliament.uk/pa/cm201617/cmselect/cmpubacc/887/887.pdf.
35. Grainger R, Pearson M, Dixon P, Devonport E, Timoney M, Bodger K, et al. Referral patterns after a seizure admission in an English region: an opportunity for effective intervention? An observational study of routine hospital data. BMJ open. 2016;6(1):e010100.
36. Blank L, Baxter S, Baird W, Reuber M. Understanding referral patterns to an epilepsy clinic: professional perceptions of factors influencing the referral of older adults. Seizure. 2013 22(9):698-702.

For peer review only

For peer review only - http://bmjopen.bmj.com/site/about/guidelines.xhtml

37. Ziso B, Dixon PA, Marson AG. Epilepsy management in older people: Lessons from National Audit of Seizure management in Hospitals (NASH). Seizure. 2017 50:33-7.
38. Alderwick H, Dunn P, McKenna H, Walsh N, Ham C. Sustainability and transformation plans in the NHS: How are they being developed in practice? : The King's Fund; 2016 [Available from: https://www.kingsfund.org.uk/sites/default/files/field/field_publication_file/STPs_in_NHS_Kings_Fund_Nov_2016.pdf.
39. NICE. Epilepsy in adults: Briefing Paper: NICE; 2012 [Available from: nice.org.uk/guidance/qs26/documents/epilepsy-in-adults-briefing-paper2.
40. National Clinical Guideline Centre. Pharmacological Update of Clinical Guideline 20. The Epilepsies: The Diagnosis and Management of the Epilepsies in Adults and Children in Primary and Secondary Care London: National Clinical Guideline Centre (UK); 2012 [Available from: ncbi.nlm.nih.gov/pubmedhealth/PMH0068980.
41. Imison C, Curry N, Holder H, Castle-Clarke S, Nimmons D, Appleby J, et al. Shifting the balance of care: great expectations. Research Report: Nuffield Trust; 2017 [Available from: https://www.nuffieldtrust.org.uk/files/2017-02/shifting-the-balance-of-care-report-web-final.pdf.
42. Day J. Care Given to Patients Treated for Epileptic Seizure by the London Ambulance Service. London: London Ambulance Service Clinical Audit and Research Unit; 2011.
43. Dickson JM, Taylor LH, Shewan J, Baldwin T, Grünewald R, Reuber M. Cross-sectional study of the prehospital management of adult patients with a suspected seizure (EPIC1). BMJ Open. 2016;6:e010573.
44. Marks P, Daniel T, Afolabi O, Spiers G, Nguyen-Van-Tam J. Emergency (999) calls to the ambulance service that do not result in the patient being transported to hospital: an epidemiological study. Emergency Medicine Journal. 2002;19:449–52.
45. Burrell L, Noble A, Ridsdale L. Decision-making by ambulance clinicians in London when managing patients with epilepsy: a qualitative study. Emergency Medicine Journal. 2013;30:236–40.
46. Noble A, Snape D, Goodacre S, Jackson M, Sherratt F, Pearson M, et al. Qualitative study of paramedics' experiences of managing seizures: a national perspective from England. BMJ Open. 2016;6:e014022.
47. Sherratt F, Snape D, Goodacre S, Jackson M, Pearson M, Marson A, et al. Paramedics' views on their seizure management learning needs: a qualitative study in England. BMJ Open. 2017;7:e014024.
48. Snooks H, Kearsley N, Dale J, Halter M, Redhead J, Cheung W. Towards primary care for non-serious 999 callers: results of a controlled study of "Treat and Refer" protocols for ambulance crews. Quality and Safefy in Health Care. 2004;13:435–43.
49. House of Commons Health Committee. Winter pressure in accident and emergency departments, 2016 HC 277 2016 [Available from: publications.parliament.uk/pa/cm201617/cmselect/cmhealth/277/277.pdf.
50. NHS. The NHS Long Term Plan 2019 [Available from: https://www.longtermplan.nhs.uk/.
51. NHS England. Transforming urgent and emergency care services in England: Clinical models for ambulance services. Urgent and Emergency Care Review Programme Team. 2015 [Available from: nhs.uk/NHSEngland/keogh-review/Documents/UECR-ambulance-guidance-FV.PDF.
52. Michie S, West R, Campbell R, Brown J, Gainforth H. ABC of Behaviour Change Theories. UK: Silverback Publishing; 2014.
53. Fisher JD, Freeman K, Clarke A, Spurgeon P, Smyth M, Perkins GD, et al. Patient safety in ambulance services: a scoping review. 2015.
54. Turner J, Coster J, Chambers D, Phung V-H, Knowles E, Bradbury D, et al. What evidence is there on the effectiveness of different models of delivering urgent care? A rapid review. 2015.
55. Health & Social Care Information Centre. Ambulance Services, England 2014-15. 2015 [Available from: digital.nhs.uk/catalogue/PUB17722.
56. Frostholm L, Fink P, Christensen K, Toft T, Oernboel E, Olesen F, et al. The patients' illness perceptions and the use of primary health care. Psychosomatic Medicine 2005;67(6):997-1005.

For peer review only - http://bmjopen.bmj.com/site/about/guidelines.xhtml

57. Lowe R, Porter A, Snooks H, Button L, Evans B. The association between illness representation profiles and use of unscheduled urgent and emergency health care services. British Journal of Health Psychology. 2011;16(4):862-79.
58. Martin R, Leventhal H. Symptom perception and health care-seeking behavior. . In: Raczynski J, Leviton L, editors. Handbook of clinical health psychology: Vol 2 Disorders of behavior and health. Washington, DC, US: American Psychological Association.; 2004. p. 299-328.
59. Ross J, Stefan H, Schäuble B, Day R, Sander J. European survey of the level of satisfaction of patients and physicians in the management of epilepsy in general practice. Epilepsy & Behaviour. 2010;19(1):36-42.
60. Booker M, Purdy S, Shaw A. Seeking ambulance treatment for ‘primary care’ problems: a qualitative systematic review of patient, carer and professional perspectives. BMJ Open. 2017;7:e016832.
61. Whitehead K, Kandler R, Reuber M. Patients’ and neurologists’ perception of epilepsy and psychogenic nonepileptic seizures. Epilepsia. 2013;54(4):708–17.
62. Thompson R, Linehan C, Glynn M, Kerra MP. A qualitative study of carers' and professionals' views on the management of people with intellectual disability and epilepsy: A neglected population. Epilepsy & Behaviour. 2013;28(3):379-85.
63. Dickson JM, Rawlings GH, Grünewald RA, Miles K, Mack C, Heywood T, et al. An alternative care pathway for suspected seizures in pre-hospital care: a service evaluation. British Paramedic Journal. 2017;2(2):22-8.
64. Peterson CL, Walker C, Coleman H. ‘I hate wasting the hospital's time’: Experiences of emergency department admissions of Australian people with epilepsy. Epilepsy & Behavior. 2019;90:228-32.
65. de Bekker-Grob EW, Ryan M, Gerard K. Discrete choice experiments in health economics: a review of the literature. Health economics. 2012;21(2):145-72.
66. Clark MD, Determann D, Petrou S, Moro D, de Bekker-Grob EW. Discrete choice experiments in health economics: a review of the literature. Pharmacoeconomics. 2014;32(9):883-902.
67. Lancaster KJ. A new approach to consumer theory. Journal of political economy. 1966;74(2):132-57.
68. Greene JC, Caracelli VJ, Graham WF. Toward a conceptual framework for mixed-method evaluation designs. Educational Evaluation and Policy Analysis. 1989;11:255–74.
69. NHS England. D04 Neurosciences 2017 [Available from: https://www.england.nhs.uk/wp-content/uploads/2013/06/d04-neurosci-spec-neuro.pdf.
70. Association of British Neurologists. Acute Neurology services survey 2017. 2017 [Available from: https://www.theabn.org/media/Documents/Acute%20Neurology/ABN%20acute%20neurology%20survey%20final%2013%20March%202017.pdf
71. Brain and Spine Foundation. List of Neurocentres in the UK. [Available from: https://www.brainandspine.org.uk/wp-content/uploads/2018/03/BSF_List-of-Neurocentres-in-the-UK.pdf
72. Health and Social Care Information Centre. Summary Report 7, Hospital Accident and Emergency Activity, 2016-17 2017 [Available from: digital.nhs.uk/media/33252/Hospital-Accident-and-Emergency-Activity-2016-17-Tables/default/acci-emer-atte-eng-2016-17-data.
73. Coast J, Al-Janabi H, Sutton EJ, Horrocks SA, Vosper AJ, Swancutt DR, et al. Using qualitative methods for attribute development for discrete choice experiments: issues and recommendations. Health economics. 2012;21(6):730-41.
74. Vass C, Rigby D, Payne K. The role of qualitative research methods in discrete choice experiments: a systematic review and survey of authors. Medical Decision Making. 2017;37(3):298-313.
75. Mays N, Pope C. Rigour and qualitative research. British Medical Journal. 1995;311:109-12.

For peer review only - http://bmjopen.bmj.com/site/about/guidelines.xhtml

76. Tong A, Sainsbury P, Craig J. Consolidated criteria for reporting qualitative research (COREQ): A 32-item checklist for interviews and focus groups. International Journal for Quality in Health Care. 2007;19(6):349-57.
77. Powell G, Holmes EA, Plumpton CO, Ring A, Baker GA, Jacoby A, et al. Pharmacogenetic testing prior to carbamazepine treatment of epilepsy: patients' and physicians' preferences for testing and service delivery. British journal of clinical pharmacology. 2015;80(5):1149-59.
78. Kralj-Hans I, Goldstein LH, Noble AJ, Landau S, Magill N, McCrone P, et al. Self-Management education for adults with poorly controlled epILEpsy (SMILE (UK)): a randomised controlled trial protocol. BMC neurology. 2014;14(1):69.
79. Ritchie J, Spencer L, O'Connor W. Carrying out qualitative analysis In: Ritchie J, Lewis J, editors. Qualitative Research Practice. New York: Sage Publications; 2003. p. 219–62.
80. Spencer L, Ritchie J, O'Connor W, Morrell G, Ormston R. Analysis in Practice. . In: Ritchie J, Lewis J, McNaughton Nicholls C, Ormston R, editors. Qualitative Research Practice: A Guide for Social Science Students & Researchers. 2nd Edition ed. London: Sage; 2014.
81. Epilepsy Action. A Critical Time for epilepsy in England. A study of epilepsy service provision in England Leeds: British Epilepsy Association; 2013.
82. Bridges JF, Hauber AB, Marshall D, Lloyd A, Prosser LA, Regier DA, et al. Conjoint analysis applications in health—a checklist: a report of the ISPOR Good Research Practices for Conjoint Analysis Task Force. Value in health. 2011;14(4):403-13.
83. House of Commons Hansard. Mental Health and NHS Performance, 9 January 2017, 619. 2017 [Available from: hansard.parliament.uk/commons/2017-01-09/debates/A0C1CB51-3E77-4FD0-87D9AD36C2C11CE5/MentalHealthAndNHSPerformance.
84. Hahn GJ, Shapiro SS. A cataloging and computer program for the design and analysis of orthogonal symmetric and asymmetric fractional factorial experiments. Schenectady, NY: General Electric Research and Development Center; 1966.
85. Street DJ, Burgess L. The construction of optimal stated choice experiments: Theory and methods. London: John Wiley & Sons; 2001.
86. Ericsson KA, Simon HA. Protocol analysis: Verbal reports as data: the MIT Press; 1984.
87. Health and Social Care Information Centre. Quality and Outcomes Framework – Prevalence, Achievements and Exceptions Report, England, 2016-17. 2017 [Available from: https://files.digital.nhs.uk/publication/c/r/qof-1617-rep.pdf.
88. Lancsar E, Louviere J. Conducting discrete choice experiments to inform healthcare decision making: a user's guide. Pharmacoeconomics. 2008;26(8):661-77.
89. Orme B. Interpreting the results of conjoint analysis, getting started with conjoint analysis: strategies for product design and pricing research. Madison, Wis: Research Publishers LLC; 2010.
90. Watson V, Becker F, de Bekker-Grob E. Discrete choice experiment response rates: A meta-analysis. Health economics. 2017;26(6):810-7.
91. Hole AR. Modelling heterogeneity in patients' preferences for the attributes of a general practitioner appointment. Journal of Health Economics. 2008 27(4):1078-94.
92. James Lind Alliance. JLA Guidebook 2019 [Available from: http://www.jla.nihr.ac.uk/jla-guidebook/.
93. INVOLVE. Briefing notes for researchers: involving the public in NHS, public health and social care research Eastleigh: INVOLVE; 2012 [Available from: http://www.invo.org.uk/wp-content/uploads/2012/04/INVOLVEBriefingNotesApr2012.pdf
94. NIHR. TSC SSC Guidance 2016 [Available from: https://www.nihr.ac.uk/funding-and-support/documents/funding-for-research-studies/how-to-apply/TSC-SSC-Guidance-April-2016.pdf.
95. Craig P, Dieppe P, Macintyre S, Michie S, Nazareth I, Petticrew M. Developing and evaluating complex interventions: the new Medical Research Council guidance. Bmj. 2008;337:a1655.

For peer review only - http://bmjopen.bmj.com/site/about/guidelines.xhtml

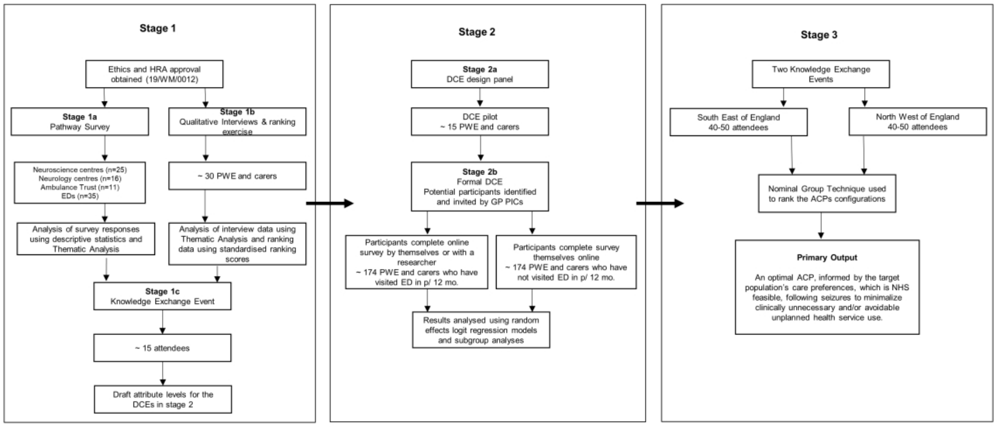

Caption : Figure 1 Research project process diagram Notes: HRA, Health Research Authority; EDs, Emergency Departments; PWE, People with Epilepsy; DCEs, Discrete Choice Experiments; ACP/s, Alternative Care pathway/s

338x190mm (300 x 300 DPI)

Supplementary File 1: Interview Topic Guide

Topics	Questions
Introduction	• Thank you very much for participating in this study. We are trying to find out more about how people with epilepsy would like to be cared for when they have a seizure and their views of the emergency services to contribute to the development of services. So I would very much like to hear about your own experiences.<br>• I wondered if you'd share with me your experiences with emergency services that may have led you to being interested in this study?
Carers	• Do you have a someone who helps with your epilepsy? (If yes, move on to support person questions. **If no, skip to next section**.)<br>◦ *(Prompts if needed: What is their relationship to you? i.e., family member, friend, paid carer? Do they live with you or somewhere else? How long have you known each other? How often would you usually see each other or have contact?)*<br>• How often have they been with you when you've had seizure in last 12 months?<br>• How confident do they feel in knowing what to do when a seizure happens?<br>◦ *Prompts if needed: Motives for contacting emergency services? Do you contact them every time after a seizure? What makes you feel more confident?*
Seizures	Perhaps first you could tell me about your seizures…<br>• What is your impression of how many seizures you have? *(Prompt: i.e., in the last year, month, year?)*<br>• How would you describe your seizures? *(Prompt: what kind do you usually have?)*<br>• How long have you been diagnosed with epilepsy?<br>• **Medication for seizures:** What do you take for your seizures? Are they helpful?<br>• How do you feel about managing your seizures? *(Prompt: do you have any strategies for managing your seizures?)*<br>• Are there any triggers or warnings before the onset of your seizures? *(Prompt: like an aura?)*<br>• How many times have emergency services been called in the past 12 months? How many of those times have you gone to A&E?
Decision Making	• I'm interested in your thoughts about the decisions that are made about your care following a seizure.<br>• Firstly, how often would you have seizures in **public** versus at **home**?<br>**Public place:**<br>◦ Can you think of a recent example and tell me where this was?<br>◦ Were you by yourself or with others (who?)

For peer review only

	• Was an ambulance called? What did they do? (i.e., take to A&E) Did you have any chance to discuss where you would like to go?<br>• Have you ever not been taken to A&E after seizure in a public place? *(Prompt: tell me more about what happened then. i.e., who was present, was this what you wanted?)*<br>• Do you have any emergency devices, or a medic alert jewellery?<br>**Home:**<br>• What do you do if a seizure happens when you are alone and no one else is around?<br>• Have **you** called an ambulance **for yourself** when you've had a seizure? If so, can you tell me a bit more about how you found this? How do you feel about the decision on whether or not to go to A&E? When **someone else** has called an ambulance after you've had a seizure, who typically makes the decision to call? Do you know if any alternatives to A&E were discussed? Who would typically make the decision for you to go to A&E?
**Emergency Services / A&E**	• Can you tell me more about what your general experiences of **Emergency Services** for your epilepsy?<br>◦ *(Prompts if needed: Where are you when the seizure(s) happen (situation and circumstances)? Do you go usually go to A&E? Who makes decision/organised E.g., carer/relative, other lay people, ambulance staff, police, etc. Is this usually what you would want?)*<br>• When was the last time you used emergency services and didn't use A&E? What happened?<br>◦ What kinds of things might change how you use emergency services? (i.e., such as location, seizure type)<br>• Can you tell me about the last time you went to **A&E** for your epilepsy?<br>◦ *Prompts if needed: Why did you go to A&E? Who made decision/organised E.g., carer/relative, other lay people, ambulance staff, police, etc.*<br>• Would you say this is typical for you?<br>• *Was this what you would have wanted?*
**Views of A&E**	If you had to use emergency services again, are there any changes that you would like to see in the way this service is provided? (Prompt if needed: and how about A&E?)<br>• What do you believe is most helpful about going to A&E? *E.g., Reassurance, confidence, treatments available, reduce burden on others. (write down)*<br>• Do you see there being anything negative about going to A&E? *E.g., Time, not necessary, unnecessary tests and examinations, disrupted rest, don't like hospitals/being patient, etc. (write down)*<br>• What has been your experience of the link-up/ communication between A&E and your usual care provider?<br>• Have your usual care providers been informed of A&E visits?<br>• Have you been contacted and offered any extra support because you had been to A&E?

For peer review only

**Alternatives to A&E**	• When you have had a seizure and contact has been made with emergency services (i.e., 999/paramedics/111), have you ever been offered an alternative to A&E? If so, what?<br>• If you weren't taken to A&E, where would you like to be taken once your seizure had finished? What health professional would you like to see at the time? What kind of follow-up would you like to have?<br><br>Are you familiar with something called an Urgent Treatment Centre?<br>*Extra Explanation, only if needed:*<br>*The term Urgent Treatment Centres is a relatively new one, but it has been suggested as a potential alternative to A&E. To give you a feel of what they are, the idea is that: -They will be open at least 12 hours a day, 7 days a week. Staffed by GPs and nurses. -Be able to issue prescriptions and have access to some common, but basic equipment, such as ECGs to test heart function and in some cases X-ray machines.*<br>• If yes, have you ever been to one before?<br>• If no, you might be more familiar with terms like 'walk-in centres' and 'minor injury units'. These are to be relabelled urgent treatment centres. *-How many there are located near you, and where they are, varies. Some are located next to GP practices, some are on hospital sites*<br>• What might be your expectations of such a centre be?<br>• Would you have any concerns about this? If so, what kind?<br>• Would anything change how you felt about this ACP? Ie., seizure type, recent changes in meds<br><br>Another option that is being considered instead of always taking people with epilepsy to A&E following a seizure, is for the person to be left at home or taken home if they were out. They would then be telephoned within say 24 hours by an epilepsy nurse specialist. The nurse would be phoning to see how the person was recovering and whether needed any additional support, such as a change in medication, advice or to be booked in to see a neurologist.<br>*Extra Explanation, only if needed/not seen an ENS before:*<br>*-An epilepsy nurse is a **registered nurse who typically has extra experience or qualifications in neurology.** Their role varies but they can provide information regarding epilepsy and its management, they can help monitor and change medication, order tests and act as a first point of contact for patients and GPs. They advise people in a variety of settings, including in outpatient clinics and over the phone.*<br>• What might be your expectations be over this sort of alternative to being taken to A&E?<br>• Would you have any concerns about this? If so, what kind?<br>• Would anything change how you felt about this ACP? Ie., seizure type, recent changes in meds

For peer review only - http://bmjopen.bmj.com/site/about/guidelines.xhtml

**Discrete Choice**	• Complete Discrete Choice / Ranking Exercise
**Characteristics**	Now I have some final short-answer questions to run through about you…<br>• What is your Date of birth?<br>• **Highest level of completed education?** (no formal qualification, 1-4GSCE, 5 or more GSCE, Apprenticeship, 2 or more A-levels, First of higher degree/professional qualification/equivalent higher education, vocational/work-related training).<br>• **Ethnicity?** (i.e., white, mixed, Asian/Asian British, Black or Black British, Chinese or other ethnic group).<br>• **Household:** What are your living arrangements? (living with others, living alone)<br>• **Other conditions:** Have you ever been diagnosed with a learning disability? Do you have any mobility difficulties?<br>**Healthcare Professionals:** Who would you contact if anything changes with your epilepsy? Have you seen them in the past 12 months?<br>• Have you seen any other people for help with your epilepsy within the last 12 months? E.g., neurologist, epilepsy nurse, GP with specialist interest in epilepsy, learning disability psychiatrist, paediatrician, neurosurgeon.
**Closing**	• Is there anything you'd like to mention about your experience of emergency services for seizure care that we haven't already discussed?<br>• Thank you very much for your time and for answering my questions. There will be a results report available at the end of the study – would you like to receive a copy?<br>• Do you have any final questions?

**Ranking exercise**	**We are keen to understand what type of care people would prefer when they have a seizure.**<br><br>**Here are some factors** that you have mentioned / or we think may be important …**<br><br>*Interviewer displays cards (Appendix 1) in a random order in front of the participant.*<br><br>Looking at these cards:<br><br>1. **Is there anything missing?** Are there any other factors what would affect your choice?<br><br>*Interviewer writes additional factors on blank cards and places them alongside the pre-defined cards in front of the participant.* ***Record any self-nominated factors on the interview record sheet (Appendix 2).***<br><br>2. **Which are the most important factors in your opinion?** Can you pick up all the cards that would most likely affect your decision?

For peer review only

	Explain that these factors can have been experienced, or not; and include any that were self-nominated in this exercise. Clear the remaining cards from the table.<br><br>**3. Now, can you rank these in order of most important to least important?**<br><br>*Check the order the interviewer places the cards is i.e. So, you think 'X' at the top is the most important? Encourage the participant to "think aloud" and explain their ranking. If participants find it hard to rank certain factors, or align them side by side, explore why.* ***Record the rank scores on the interview record sheet.***

For peer review only

Appendix 1: Show cards

Where the ambulance takes you

Where the ambulance takes you (e.g. A&E, Urgent treatment centre).

Care provider

The healthcare professional responsible for your care when you get there.

Waiting time

How long you have to wait before you see the healthcare professional.

Tests

The type of tests the health professional could carry out immediately if needed

Follow-up

Referral to see a health professional with specialist training in epilepsy

For peer review only

Appendix 2: Ranking exercise interview record sheet

Participant ID: _ _ /_ _ / _ _

Interviewer ID _ _

**Factor***	Rank Results (insert 1-5)<br>1 = most important<br>5 = least important<br>X = not selected
Anything Else? **Write here:**	
Anything Else? **Write here:**	
Anything Else? **Write here:**	
Anything Else? **Write here:**	
Anything Else? **Write here:**	
Anything Else? **Write here:**	
Where the ambulance takes you	
Care provider	
Waiting time	
Tests	
Follow-up	

For peer review only

Supplementary File 2 Illustration of a possible vignette and Discrete Choice Experiment choice that could be used

**Question 1**<br>Imagine you have a seizure in a public place. It lasted no more than usual and stopped by itself. You are tired and need to sleep. Someone called an ambulance and the paramedic is with you. Which of the following options would you prefer to happen next?	**Option A**	**Option B**
**Where**<br>*Where the ambulance takes you*	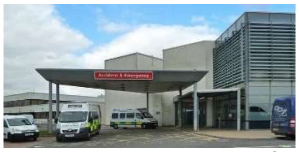<br>***Accident and Emergency (A&E)***	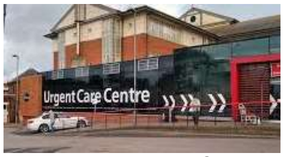<br>***Urgent Treatment Centre***
**Care provider**<br>*The health care professional responsible for your care when you get there*	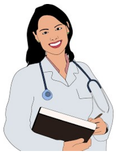<br>***Consultant doctor in emergency medicine***	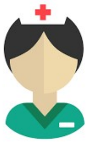<br>***Emergency nurse practitioner***
**Waiting time**<br>*How long you have to wait before you see the health care professional*	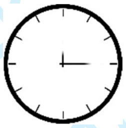<br>***4 hours***	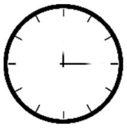<br>***1 hour***
**Tests**<br>*The type of tests the health professional could carry out immediately if needed*	***Advanced***<br>(examples...)	***Basic***<br>(examples...)
**Follow-up**<br>*The number of people referred to see a health professional with specialist training in epilepsy*	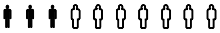<br>***3 in 10 people referred to specialist epilepsy services***	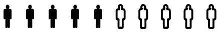<br>***5 in 10 people referred to specialist epilepsy services***
**Which service would you prefer?**	☐	☐

For peer review only
